# Inactivation of *Exosc10* in the oocyte impairs oocyte development and maturation, leading to a depletion of the ovarian reserve in mice

**DOI:** 10.7150/ijbs.72889

**Published:** 2023-01-31

**Authors:** Leïla Demini, Christine Kervarrec, Laëtitia Guillot, Emmanuelle Com, Régis Lavigne, Pierre-Yves Kernanec, Michael Primig, Charles Pineau, Fabrice G. Petit, Soazik P. Jamin

**Affiliations:** 1 Univ Rennes, Inserm, EHESP, Irset (Institut de recherche en santé, environnement et travail) - UMR_S 1085, F-35000 Rennes, France.; 2 Univ Rennes, CNRS, Inserm, Biosit UAR 3480 US 018, Protim core facility, F-35000 Rennes, France.

**Keywords:** *Exosc10*, oogenesis, follicular development, ovary

## Abstract

EXOSC10 is a catalytic subunit of the nuclear RNA exosome, and possesses a 3'-5' exoribonuclease activity. The enzyme processes and degrades different classes of RNAs. To delineate the role of EXOSC10 during oocyte growth, specific *Exosc10* inactivation was performed in oocytes from the primordial follicle stage onward using the *Gdf9*-iCre; *Exosc10*^f/-^ mouse model (*Exosc10*^cKO(Gdf9)^). *Exosc10*^cKO(Gdf9)^ female mice are infertile. The onset of puberty and the estrus cycle in mutants are initially normal and ovaries contain all follicle classes. By the age of eight weeks, vaginal smears reveal irregular estrus cycles and mutant ovaries are completely depleted of follicles. Mutant oocytes retrieved from the oviduct are degenerated, and occasionally show an enlarged polar body, which may reflect a defective first meiotic division. Under fertilization conditions, the mutant oocytes do not enter into an embryonic development process. Furthermore, we conducted a comparative proteome analysis of wild type and *Exosc10* knockout mouse ovaries, and identified EXOSC10-dependent proteins involved in many biological processes, such as meiotic cell cycle progression and oocyte maturation. Our results unambiguously demonstrate an essential role for EXOSC10 in oogenesis and may serve as a model for primary ovarian insufficiency in humans. Data are available via ProteomeXchange with identifier PXD039417.

## Introduction

Oogenesis is a complex process that ultimately gives rise to a competent female gamete, the oocyte. At the time of ovulation, the oocyte quality is essential for the proper embryonic development. In the mouse fetal ovary, oogonia (oocyte precursors) are enclosed in cysts and undergo several rounds of mitotic divisions before entering meiosis and giving rise to the oocyte 1. Before birth, the cyst breakdown leads to massive germ cell death 2. The remaining oocytes, arrested in meiotic prophase I, are associated with surrounding granulosa cells and form the primordial follicle pool. After birth, folliculogenesis is initiated and a constant dialogue is established between oocytes and granulosa cells 3. During folliculogenesis, the oocyte enters in a transcriptional quiescence phase and acquires both meiotic and developmental competences 4. The oocyte synthesizes and accumulates maternal mRNAs and proteins that are critical for the first embryonic divisions and subsequent development 5. After fertilization, the zygote will rely on this pool of maternal mRNAs and proteins to continue its journey until zygotic genome activation, which occurs around the two-cell stage in mice 6. Therefore, any disorders during oocyte maturation will affect the embryo quality and subsequently the ovarian reserve and/or the early embryonic development.

Like in any other cell type, mRNAs and rRNAs in oocytes are processed by the RNA exosome. The RNA exosome is a highly conserved multiprotein complex. It is composed of a core of nine subunits associated with two subunits possessing ribonucleolytic activities: EXOSC10 is localized in the nucleus and nucleolus, while DIS3 is localized in the nucleus and the cytoplasm. EXOSC10 is important for the 3' maturation of the 5.8s rRNA in yeast 7 and in pre-rRNA precursors processing in human 8. Recent work using a mouse *Exosc10* gene deletion and targeted inactivation via the Cre-lox system revealed an important role in spermatogonia 9, and a developmental arrest at the eight-cell/morula transition; we note that EXOSC10 was found to accumulate at the periphery of nucleolus precursor bodies (NPBs), which suggests an important role in embryonic rRNA processing and protein synthesis 10.

Recently, Wu and Dean employed a *Zp3-*Cre mouse line to specifically inactivate* Exosc10* in oocytes at the primary follicular stage 11. *Exosc10*^f/-^; *Zp3-*Cre females are subfertile and generate a large fraction of incompetent oocytes *i.e.* oocytes with impaired endomembrane components, and delayed germinal vesicle breakdown. This delay is due to CDK1 activation failure, which in turn blocks disassembly of the lamina. Single oocyte RNA profiling revealed dysregulation of multiple types of RNA, and showed that EXOSC10 promotes the transition from oocyte growth to maturation by sculpting the transcriptome in order to support the maturation phase of oogenesis 11.

To investigate the role of EXOSC10 during oogenesis, we have generated *Gdf9-*iCre*; Exosc10*^f/-^ (or *Exosc10*^cKO(Gdf9)^) mice that lack functional *Exosc10* specifically in oocytes from the primordial stage onward. We find, contrary to earlier work, that the female mutant mice are infertile 11. While there are no visible differences between control and *Exosc10*^cKO(Gdf9)^ ovaries in young females, we observed a complete depletion of oocytes in mutant ovaries after eight weeks of age. *Exosc10*^cKO(Gdf9)^ mice show a normal onset of puberty and a regular estrus cycle at the beginning of their reproductive life that, however, quickly becomes irregular since the estrus is absent at around eight weeks post-partum. Proteomic profiling reveals that EXOSC10 depletion in oocytes disrupts molecular pathways governing the cell cycle, nuclear transport, formation of the nuclear envelope, RNA processing and surveillance, oocyte meiosis, DNA replication and DNA repair. Our work establishes an essential role for *Exosc10* in oocyte growth and maturation and may serve as a model for primary ovarian insufficiency in humans.

## Materials and methods

### Ethics Statement

All animal experiments (housing, care, method of euthanasia), were performed in agreement with the recommendations of the French Accreditation of Laboratory Animal Care. The French Ministry of Research approved the project (APAFIS#25237-2020042715505401 v2) and the French Ministry of Agriculture has delivered the license for the animal facility (D35-238-19). Superovulation and vasectomy procedures were performed by F. Petit who is licensed for animal experimentation and surgery by the French Ministry of Agriculture.

### Mice

To specifically inactivate *Exosc10* in the ovarian germ line, we used the STOCK Tg(*Gdf9-*iCre)5092Coo/J (*Gdf9-*iCre, The Jackson Laboratory, SN011062) mouse line 12, and the *Exosc10*^+/-^ and *Exosc10*^f/f^ mouse lines 9,10. *Gdf9-*iCre mice were crossed with *Exosc10*^+/-^ mice to generate *Exosc10*^+/-^; *Gdf9-*iCre mice. For the final cross, *Exosc10*^+/-^; *Gdf9*-iCre male mice were crossed with *Exosc10*^f/f^ female mice to generate the mutant *Exosc10*^f/-^; *Gdf9*-iCre mice (also named *Exosc10*^cKO(Gdf9)^). *Exosc10*^+/f^ mice were used as controls. Mouse genotyping was performed at weaning and after euthanasia. Genomic DNA was extracted from tail tips and subjected to a standard PCR assay as described in Petit et al. 10. The set of primers used to discriminate the different *Exosc10* alleles (wild type or +, knock-out or -, floxed or f, and conditional knock-out or cKO) and to detect the *Gdf9*-iCre transgene are listed in Supplementary Fig. S1. C57BL/6NRj mice used in this study were purchased from Janvier Labs (Le Genest-Saint-Isle, France).

### Puberty, estrus cycle and fertility assays

All reproductive performance assays described in this section were performed on *Exosc10*^+/f^ and *Exosc10*^cKO(Gdf9)^ female mice. To assess the onset of puberty, mice were monitored daily for vaginal opening from three weeks of age. Estrus cycles of 7-week-old female mice were monitored for five weeks by daily microscopic examination of vaginal smears. A vaginal lavage is performed with PBS and the collected vaginal fluid is stained with 0.1% methylene blue (Sigma, Saint-Louis, MO, USA). The different phases of the estrus cycle were characterized as follows: the estrus stage contains anucleate cornified cells; the metestrus stage is composed primarily of clustered cornified cells then of cornified epithelial cells, leukocytes and mucus; the diestrus stage is characterized by predominant leukocytes; the proestrus stage is determined by the presence of predominant nucleated epithelial cells 13. Fertility tests were performed by mating 8-week-old female mice with a stud male during six months. Mice were checked daily for vaginal plugs and pups were counted in each litter.

### Ovaries collection for follicle count and immunohistochemistry

For juvenile female mice (three to eight weeks of age), ovaries were collected randomly for both *Exosc10*^+/f^ and *Exosc10*^cKO(Gdf9)^ mice. Eight- to ten-week-old* Exosc10*^+/f^ or *Exosc10*^cKO(Gdf9)^ female mice were mated with vasectomized C57BL/6NRj males. After a vaginal plug was observed (pseudogestational day 1 or PGD1), ovaries were collected. With* Exosc10*^cKO(Gdf9)^ female mice, an absence of vaginal copulatory plug was frequently observed from 8 weeks of age onwards, prompting us to collect randomly the ovaries from 10 weeks old. All collected ovaries were fixed either in Bouin's solution overnight at room temperature for hematoxylin/eosin (Leica Biosystems, Wetzlar, Germany) staining or in 4% paraformaldehyde overnight at 4 °C for immunohistochemistry assay. Then samples were dehydrated and embedded in paraffin (Histowax, Guthenburg, Sweden).

### Follicle count

Ovaries were serially sectioned at 5 μm using a microtome (Shandon, Pittsburgh, PA, USA). One out of 5 sections were mounted on glass slides to avoid counting the same follicle several times, dried, rehydrated and stained with hematoxylin and eosin. A minimum of 3 ovaries have been counted for each group and age. Follicle stage was determined using the Pedersen's classification 14. A follicle was counted only if a healthy oocyte nucleus was visible in order to avoid recording atretic oocytes. Follicles were counted on at least 10 sections per ovary and the percentage of each follicular stage regarding the total number of follicles was represented. Histological sections were analyzed using a NanoZoomer 2.0-RS (Hamamatsu Photonics, Hamamatsu, Japan) and the NDP.view2 viewing software (Hamamatsu Photonics).

### Immunohistochemistry analysis

Ovary sections (5 μm) were subjected to epitope retrieval and peroxidase blocking as previously described with citrate buffer (Neobiotech, Nanterre, France) and Peroxidase Blocking Reagent (Ready-to-use 3% H_2_O_2_, Neobiotech), respectively 9. Immunostainings for VASA (rabbit ab13840, 1:1000; Abcam, Cambridge, UK) and αSMA (mouse clone 1A4, 1:2000; Sigma) were performed according to the manufactory instructions of the NeoStain ABC kit, HRP, Mouse and Rabbit (NeoBiotech). For AMH (goat SC-6886, 1:500; Santa Cruz Biotechnology, Santa Cruz, CA, USA), immunostainings were performed as previously described except that the biotinylated secondary antibody was diluted in DAKO antibody diluent (DAKO, Glostrup, Denmark). All immunostainings were revealed with DAB Quanto (Thermo Scientific, Kalamazoo, USA), counterstained with Hematoxylin and dehydrated before mounted using Eukitt (Dutscher, Brumath, France).

EXOSC10 (rabbit ab50558, 1:800; Abcam) immunostainings were carried out on the Discovery Automated IHC stainer using the Ventana DABMap detection kit (#760-124, Ventana Medical Systems, Tucson, Ariz) at the H2P2 core facility (Biosit, Rennes, France). Applications of the Ventana High Temperature Liquid Coverslip (LCS, #650010, Ventana) occurred throughout the automated protocol as appropriate. Likewise, the slides were rinsed between steps with Ventana Tris-based Reaction buffer (#950-300, Ventana). Following deparaffination with Ventana EZ Prep solution (#950-100, Ventana) at 75 °C for 8 min, antigen retrieval was performed using Ventana proprietary, Tris-based buffer solution CC1 for antibody at 100 °C for 60 min. Endogen peroxidase was blocked with Inhibitor-D 3% H_2_O_2_ (Ventana) for 4 min at 37 °C. Slides were incubated at 37 °C for 60 min with EXOSC10 primary antibodies. Biotinylated goat anti rabbit immunoglobin IgG(H+L) secondary antibody (BA-1000 Vector laboratory, Burlingame, CA, USA) was applied and incubated 32 min at 37°C. Signal enhancement was performed using the Ventana DABMap Kit. Slides were counterstained with hematoxylin and coverslipped. Histological sections were analyzed using an AxioImager microscope equipped with an AxioCam ICc1 camera and ZEN v.2.3 (Blue edition, Zeiss, Oberkochen, Germany) or using a NanoZoomer 2.0-RS and the NDP.view2 viewing software (Hamamatsu Photonics).

### Embryo culture

Six- to eight-week-old *Exosc10*^+/f^ and *Exosc10*^cKO(Gdf9)^ mice were mated with stud C57BL/6NRj males until a vaginal plug was observed (gestational day 1 or GD1). Female mice were euthanized in the morning of GD1. The oviducts were collected and flushed with EmbryoMAX Advanced KSOM medium (aKSOM, MR-101-D; Millipore, Temecula, CA, USA) containing 1 mg/ml of bovine hyaluronidase type I-S (H3506; Sigma) to recover the putative zygotes. After 5-10 min of incubation, the cumulus cells were dissociated and all putative embryos were then washed 3 times in fresh aKSOM medium. Eggs were cultured individually in 100 µL aKSOM medium in a 96-well plate for 5 days in a humidified incubator with 5% CO2 at 37 °C. Day one of culture corresponds to the day of embryo recovery. Brightfield images were taken every 24 hours (between 10:30 a.m. and 12:30 p.m.) using a CELENA S Digital Imaging System (Logos Biosystems, Villeneuve d'Ascq, France).

### Superovulation and oocyte analysis

Oocytes were obtained from 3-4 week-old *Exosc10*^+/f^ and *Exosc10*^cKO(Gdf9)^ mice after superovulation. After an initial intraperitoneal injection of 10 units of pregnant mare serum gonadotropin (PMSG, Chrono Gest PMSG-500, Intervet) followed 48 h later by 10 units of human chorionic gonadotropin (hCG, Chorulon 1500, Intervet), the cumulus-oocyte complexes were recovered from the ampulla of the oviduct in aKSOM medium containing 1 mg/ml of bovine hyaluronidase type I-S. After 5-10 minutes of incubation, oocytes were collected, washed 3 times in fresh aKSOM medium before being transferred to 100 µL aKSOM medium in a 96-well plate. DNA content can be visualized by incubating the oocytes in 10 μM of fluorescent Vybrant DyeCycle Green Stain (Invitrogen, Karlsbad, CA, USA) for 30 min. Brightfield and fluorescent images of the oocytes were taken using a CELENA S Digital Imaging System (Logos Biosystems, Villeneuve d'Ascq, France).

### Protein extraction

Ovaries were collected from 8- to 10-week-old *Exosc10*^+/f^ (at PGD1, n=5) and *Exosc10*^cKO(Gdf9)^ (randomly, n=4) mice and conserved at -80 °C until protein extractions. Ovaries were ground to fine powder in liquid nitrogen and lysed in 30 mM Tris buffer pH 7.4 containing 8 M urea, 4% CHAPS, protease inhibitors (1 mM EDTA, 0.5 mM dithiothreitol (DTT), 1 mM 4-(2-Aminoethyl)benzenesulfonyl fluoride hydrochloride (AEBSF), and 10 μM l-trans-Epoxysuccinyl-leucylamido(4-guanidino)butane (E64)), using an ultrasonic processor (Bioblock Scientific, Illkirch, France) 6 times for 10 sec, with a 30 sec stop between each step, with a microtip setting power level at 40% pulse duration. After centrifugation (15,000 × g for 15 min at 4 °C) to remove cellular debris and ultracentrifugation at 105,000 × g for 1 h at 4 °C to remove subcellular particles, cytosoluble proteins were stored at -80 °C until further analysis. Total protein concentration in each sample was determined using the Bradford Protein Assay Kit according to the manufacturer's instructions (Bio-Rad, Marnes-la-Coquette, France).

### Protein digestion, Mass spectrometry acquisition and Protein identification and differential quantification

Proteins (2 µg) were in-gel digested with 12.5 ng/μL sequencing grade modified trypsin (Promega, Charbonnières-les-Bains, France) after concentration on one band using a 4-12% Bis-Tris precast gel (NuPAGE^™^) as previously described 15. The resulting peptide mixtures were injected onto a TimsTOF Pro instrument (Bruker Daltonik GmbH, Bremen, Germany) coupled to a NanoElute HPLC System equipped with on a 75 µm × 250 mm IonOpticks Aurora 2 C18 column (Ion Opticks Pty Ltd, Fitzroy, Australia) and LC-MS/MS data were acquired using the PASEF method as previously described 16. MS data were processed with the Data Analysis 5.1 software and peptide and protein identification were performed using the Mascot (Mascot server v2.5.01; http://www.matrixscience.com) database search engine and its automatic decoy database search to calculate a false discovery rate (FDR). MS/MS spectra were queried against the *Mus musculus* UniProt KB proteome database (release UP000000589; 21959 sequences) and a common contaminant database such as trypsin, keratins, BSA, from the Max Planck Institute of Biochemistry, available in Mascot (247 sequences). Mass tolerance for MS and MS/MS was set at 15 ppm and 0.05 Da. The enzyme selectivity was set to full trypsin with one miscleavage allowed. Protein modifications were fixed carbamidomethylation of cysteines, variable oxidation of methionine. Identification results from Mascot (.dat files) were imported into the Proline Studio software 17. This software was then used to validate protein identification with a peptide rank = 1, a peptide score > 30 and a 1% FDR at the peptide spectrum matches level. Proline Studio software was also used to the spectral count comparison of the identified proteins in each samples as previously described 18. The mass spectrometry proteomics data have been deposited to the ProteomeXchange Consortium via the PRIDE 19 partner repository with the dataset identifier PXD039417 and 10.6019/PXD039417.

### Proteomic data analysis

Differentially detected protein list was imported into Cytoscape 20 3.8.2 by using Stringapp 1.7 for visualizing molecular interaction networks and biological pathways with expression data. According to label-free data, fill color of nodes was set by a continuous mapping : log Ratio (FC) < 0 in blue and log Ratio (FC) > 0 in red. Proteins with a log2 ratio > 1 are considered as up-regulated in *Exosc10*^cKO(Gdf9)^ and with a log2 ratio < -1 are considered as down-regulated in *Exosc10*^cKO(Gdf9)^ ovaries. Transcriptomics data downloaded from https://www.ncbi.nlm.nih.gov/geo/query/acc.cgi?acc=GSE141190; (Wu and Dean, 2020) (ARN polyA only, and stage GV; 7 controls and 15 *Exosc10* oocyte-specific knockout) were submitted to the R package DESeq2 21. Data were added to be visualized by a data table import into Cystoscape. According to transcriptomics data, border paint of nodes was set by a continuous mapping: Log2(FC) *Exosc10*^+/f^ vs *Exosc10*^cKO(Gdf9)^ < 0 in blue and Log2(FC) *Exosc10*^+/f^ vs *Exosc10*^cKO(Gdf9)^ > 0 in red. Gene Ontology (GO) terms enrichment was performed using ClusterProfiler 22 via Galaxy (https://usegalaxy.fr/). GO terms with a *p-*value<0.05 were considered significantly enriched.

### Quantitative PCR analysis

RNA extractions were performed using the RNeasy Plus Micro Kit (Qiagen, Hamburg, Germany) and integrity of the different RNA (9 < RIN < 9,6) was tested with 2100 Bioanalyzer instrument (Agilent, Santa Clara, CA, USA). Reverse transcription was performed using iScript cDNA synthesis Kit (Bio-Rad) and the quantitative PCR using the iTaq Universal SYBR Green Supermix (Bio-Rad). The normalized relative expression for each target gene (ΔΔCq) and statistical analyses were performed with CFX Maestro software (Bio-Rad v 2.2.). Gene expression was measured between *Exosc10*^+/f^ and *Exosc10*^cKO(Gdf9)^ biological groups, each containing six biological replicates and two technical replicates. *Rplp0* and *Rpl13a* are used as reference genes. Primer sequences are listed in Table S1.

### Statistical analysis

Statistical analyses were performed with data from at least 3 animals per group using Graph Pad Prism 6. Follicular distribution was statistically tested using an unpaired two-way ANOVA. Other results were tested using an unpaired parametric Student's test. Before using parametric tests, the mean and the median were checked for equivalence verifying the symmetry of the data. The results were presented as mean ± s.e.m. but statistics were calculated using the s.d. A difference between two groups is considered significant when the *p*-value was < 0,05. *p<0.05, **p<0.01, ***p<0.001, ****p<0.0001.

## Results

### Targeted inactivation of *Exosc10* in oocytes impairs the estrus cycle and causes infertility

**Figure 1:**
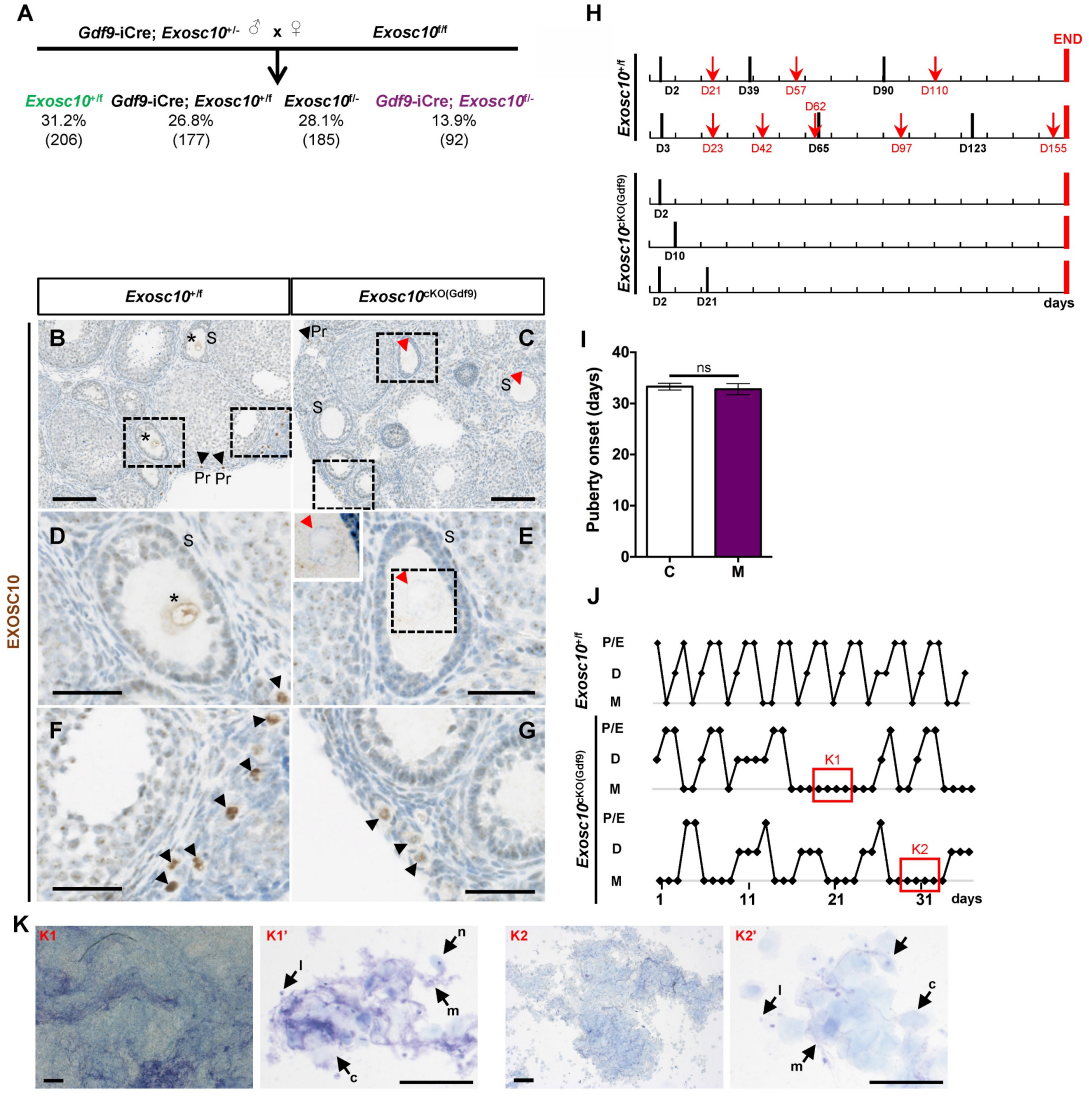
** Oocyte specific inactivation of *Exosc10* leads to an impairment of the estrus cycle in *Exosc10*^cKO(Gdf9)^ mice.** (**A**) Percentages obtained for each genotype from the cross between a male *Exosc10*^+/-^; *Gdf9*-iCre and a female *Exosc10*^f/f^. Control mice (*Exosc10*^+/f^) and conditional mutant mice (*Gdf9-*iCre*; Exosc10*^f/-^ or *Exosc10*^cKO(Gdf9)^) are represented in color. Number of mice is given in parentheses. (**B-G**) Immunohistochemistry analysis of EXOSC10 in ovaries of 4-week-old control (B,D,F) and *Exosc10*^cKO(Gdf9)^ (C,E,G) mice. (D-G) High magnifications of the respective marked area in B and C. EXOSC10 is expressed in the nucleus of secondary follicles in control (B,D, asterisks) but is absent in mutant (C,E, red arrowheads). The insert in E outlines the absence of Exosc10 labelling. Primordial follicles (black arrowheads) from control (F) and mutant (G) express EXOSC10. Scale bars: 100 µm (B,C), 40 µm (D-G and insert in E). (**H**) Monitoring of vaginal plugs (black bars) and litters (red arrows) for two *Exosc10*^+/f^ and three *Exosc10*^cKO(Gdf9)^ mice during 160 days (red bars mark the end of monitoring). (**I**) Puberty onset after a daily monitoring of vaginal opening. *Exosc10*^+/f^ (C), n=14; *Exosc10*^cKO(Gdf9)^ (M), n=10. Two-tailed Student's test. ns, not significant. (**J**) Histograms representing the estrus cycle for one control and two *Exosc10*^cKO(Gdf9)^ 7-week-old mice. P/E, Proestrus/Estrus; D, Diestrus; M, Metestrus. (**K**) Representative images of abnormal estrus cycle stages observed in two different fields of vaginal smears from *Exosc10*^cKO(Gdf9)^ mice. Vaginal smears contain abundant mucus (m), leukocytes (l), cornified epithelial cells (c) and nucleated epithelial cells (n). Scale bars: 200 µm (K1,K2), 100 µm (K1',K2').

To investigate the role of *Exosc10* during oogenesis, we used a Cre/*loxP* system to generate a conditional mutant mouse line that lacks functional *Exosc10* in oocytes. We initially crossed the mice harboring the *Exosc10*-null allele (previously described 9,10 and Fig. S1A) with the *Gdf9-*iCre mouse line to express the Cre recombinase in oocytes from the primordial follicle stage onwards 12. The resulting *Gdf9-*iCre*; Exosc10*^+/-^ mice are then crossed with the floxed *Exosc10* mouse line to generate oocyte-specific *Gdf9-*iCre*; Exosc10*^f/-^ (*Exosc10*^cKO(Gdf9)^) mutant mice; *Exosc10*^+/f^ littermates were used as controls 9. As shown in Fig. 1A, the conditional mutant mice *Exosc10*^cKO(Gdf9)^ and the control *Exosc10*^+/f^ mice were not obtained at the expected Mendelian frequency (13.9% and 31.2%, respectively). We used specific sets of primers to distinguish the floxed allele (f) from the conditional knock-out allele (cKO), the latter being generated after processing of the floxed allele by the Cre recombinase (Fig. S1A,B). Among all genotyped *Gdf9-*iCre*; Exosc10*^+/f^ mice, approximately 23% show global recombination of the floxed allele (Fig. S1C, lane 4, 324 bp product). These results confirmed that the *Gdf9-*iCre mouse line presented an ectopic expression of the Cre recombinase 12 leading to the embryonic lethality of the affected *Exosc10*^cKO(Gdf9)^ mice.

To confirm the specific inactivation of *Exosc10* in oocytes, we analyzed the expression of EXOSC10 using immunohistochemistry on paraffin-embedded sections from 4-week-old *Exosc10*^cKO(Gdf9*)*^ovaries (Fig. 1B-G). As expected, EXOSC10 was present in the nucleus of oocytes of primordial follicles in both control and mutant mice (Fig. 1B,C,F,G). However, as oocyte development continues, EXOSC10 is still expressed in the nucleus of oocytes of growing follicles in control ovaries but is lacking in *Exosc10*^cKO(Gdf9*)*^ovaries (Fig. 1D,E). This confirms that our targeted gene inactivation approach is effective.

To assess the reproductive performances of *Exosc10*^cKO(Gdf9*)*^ mice, six 8-week-old control and *Exosc10*^cKO(Gdf9*)*^ female mice were mated with stud C57BL/6NRj males during a period of six months, and vaginal plugs and neonates were monitored daily. While control mice had regular plugs and gave birth to 194 cumulative pups in 25 litters, *Exosc10*^cKO(Gdf9)^ mice had a few plugs before the age of 12 weeks and no pups over the six month period (Table 1 and Fig. 1H). Next, we analyzed more precisely the reproductive behavior and functions of the mutant mice. The onset of puberty as assessed by the time of the vaginal opening did not present any significative differences (Fig. 1I, 32.8 ± 3.4 days; n = 10 for mutants versus 33.3 ± 2.5 days; n = 14 for controls). We performed daily vaginal smears and cytological analyses from 7-week-old control and *Exosc10*^cKO(Gdf9*)*^ mice over a period of five weeks (Fig. 1J). While control mice show regular estrus cycles, we found that *Exosc10*^cKO(Gdf9*)*^ mice exhibit impaired estrus cycles (Fig. 1J). Until eight to ten weeks of age, vaginal smears of *Exosc10*^cKO(Gdf9*)*^ appeared to be normal, and afterwards the cycle was irregular with prolonged metestrus-like phases containing abundant clustered cornified cells, leukocytes and mucus (Fig. 1K). Taken together, these results suggest that *Exosc10*^cKO(Gdf9*)*^ mice undergo normal onset of puberty but then lose their sexual receptivity and cyclicity between eight and ten weeks of age before they become infertile.

### *Exosc10* is essential for oocyte survival and maintenance of the ovarian reserve

**Figure 2:**
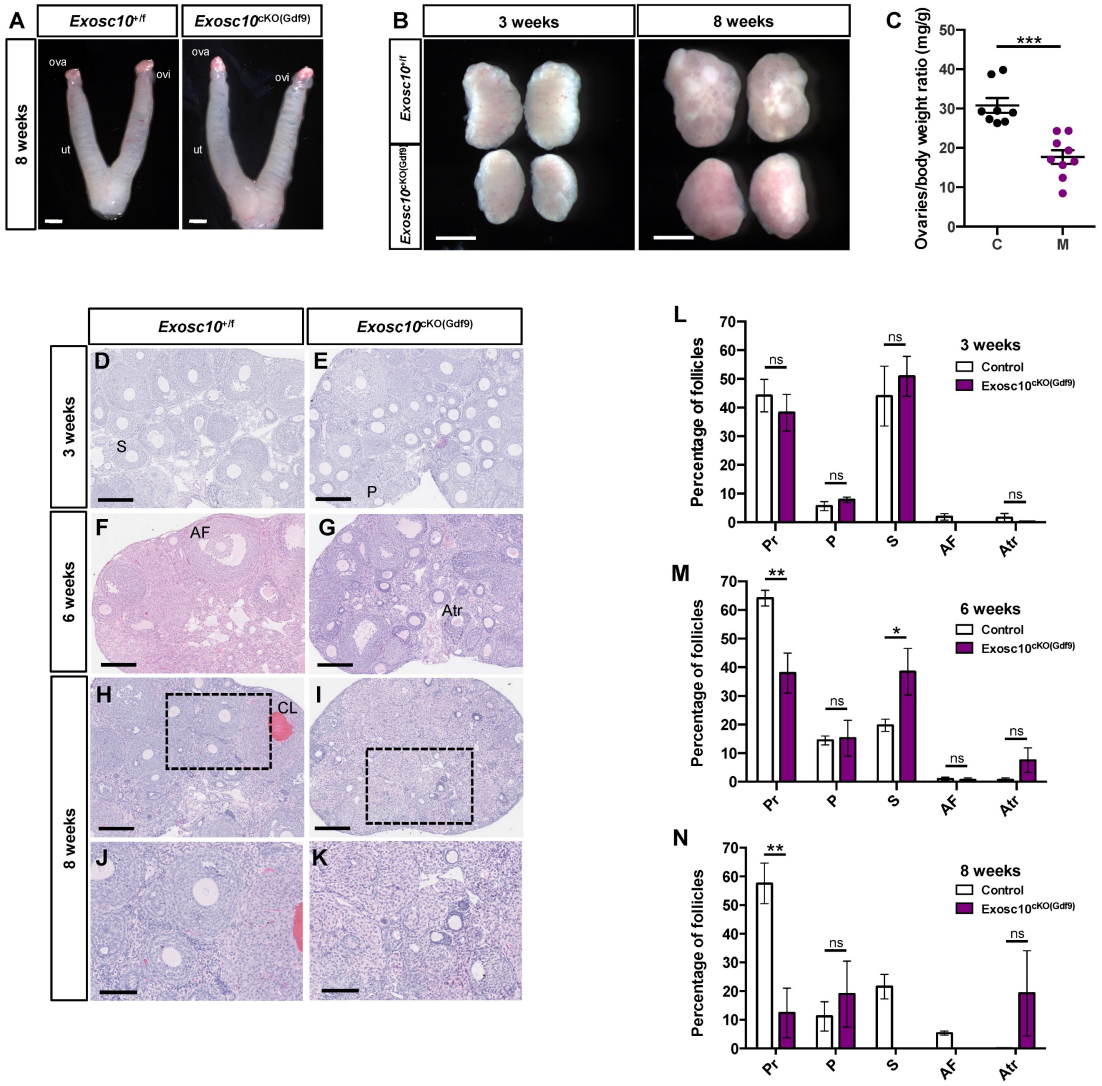
***Exosc10* inactivation in primordial follicles leads to a rapid depletion of oocytes in ovaries.** (**A**) Gross morphology of 8-week-old *Exosc10*^+/f^ and *Exosc10*^cKO(Gdf9)^ female genital tracts collected at pseudo-gestational day 1 (PGD1). Ova, ovary; ovi, oviduct; ut, uterus. Scale bars: 2 mm. (**B**) Ovaries from 3- and 8-week-old *Exosc10*^cKO(Gdf9)^ female mice appear smaller than those of control mice. Scale bars: 1 mm. (**C**) Scatter plot representing the weight ratio ovaries/body weight at eight weeks old. *Exosc10*^+/f^ (C), n=8; *Exosc10*^cKO(Gdf9)^ (M), n=9. Two-tailed Student's test. ***p<0.001. (**D-K**) Paraffin embedded sections of ovaries stained with hematoxylin/eosin at three weeks (D,E), six weeks (F,G) and eight weeks (H,I,J,K). Scale bars: 200 µm. P, Primary follicle; S, Secondary follicle; Atr, Atretic follicle; AF, Antral Follicle; CL, Corpus Luteum. J, K are high magnifications of H and I respectively. Scale bars: 100 µm. (**L-N**) Percentage for each follicular development stage in 3-, 6- and 8-week-old *Exosc10*^+/f^ and *Exosc10*^cKO(Gdf9)^ ovaries. Three weeks : *Exosc10*^+/f^, n=3, *Exosc10*^cKO(Gdf9)^, n=4; six weeks : *Exosc10*^+/f^, n=3, *Exosc10*^cKO(Gdf9)^, n=3; eight weeks : *Exosc10*^+/f^, n=4, *Exosc10*^cKO(Gdf9)^, n=4. Pr, Primordial follicle; P, Primary follicle; S, Secondary follicle; AF, Antral Follicle; Atr, Atretic follicle. Two-way ANOVA. ns, not significant; *p<0.05, **p<0.01.

To better understand what causes the reproductive dysfunctions observed in *Exosc10*^cKO(Gdf9*)*^mice, we collected 8-week-old control and *Exosc10*^cKO(Gdf9*)*^ reproductive tracts at pseudo gestational day 1 (PGD1). We observed that both control and *Exosc10*^cKO(Gdf9*)*^mice displayed swollen uteri, which indicates that the uterine glands function normally (Fig. 2A). However, due to the deregulation of the estrus cycle in most 8-week-old *Exosc10*^cKO(Gdf9*)*^ mice, vaginal plugs were not observed, and therefore reproductive tracts were collected randomly. Anatomical analyses of ovaries from 3- and 8-week-old control and *Exosc10*^cKO(Gdf9*)*^mice showed that mutant ovaries were significantly smaller than those from controls (ovary/body weight of 30.8 ± 5.4 mg/g and n=8 for the controls versus 17.7 ± 5.3 mg/g for the mutants and n=9) (Fig. 2B and C). Apart from their size, there were no macroscopic differences between controls and mutants at three weeks of age (Fig. 2B). By eight weeks, control ovaries had visible corpora lutea whereas *Exosc10*^cKO(Gdf9*)*^ovaries kept a smooth and juvenile appearance (Fig. 2B). Paraffin-embedded sections of ovaries stained with hematoxylin/eosin showed no histological differences between 3-week-old control and *Exosc10*^cKO(Gdf9*)*^ ovaries (Fig. 2D, E). A follicular count confirmed that the distribution of the follicles was not significantly different in immature mice (Fig. 2L). At the age of six weeks, the *Exosc10*^cKO(Gdf9)^ ovary sections displayed slight differences with control ovaries such as the presence of atretic oocytes (Fig. 2F, G). The follicular count established that 6-week-old *Exosc10*^cKO(Gdf9)^ ovaries had significantly fewer primordial and more secondary follicles than control ovaries (Fig. 2M). In addition, the mutant ovaries contained more atretic follicles, however, this observation was not statistically significant. At eight weeks, *Exosc10*^cKO(Gdf9)^ ovaries were completely devoid of oocytes and corpora lutea; these ovaries contained oocyte-depleted follicles and vacuoles containing degenerated oocytes (Fig. 2H-K). The corresponding follicular count confirmed that 8-week-old* Exosc10*^cKO(Gdf9)^ ovaries are made up of only few primordial and primary follicles. At this stage, no secondary and antral follicles were observed (Fig. 2J, K, N).

**Figure 3:**
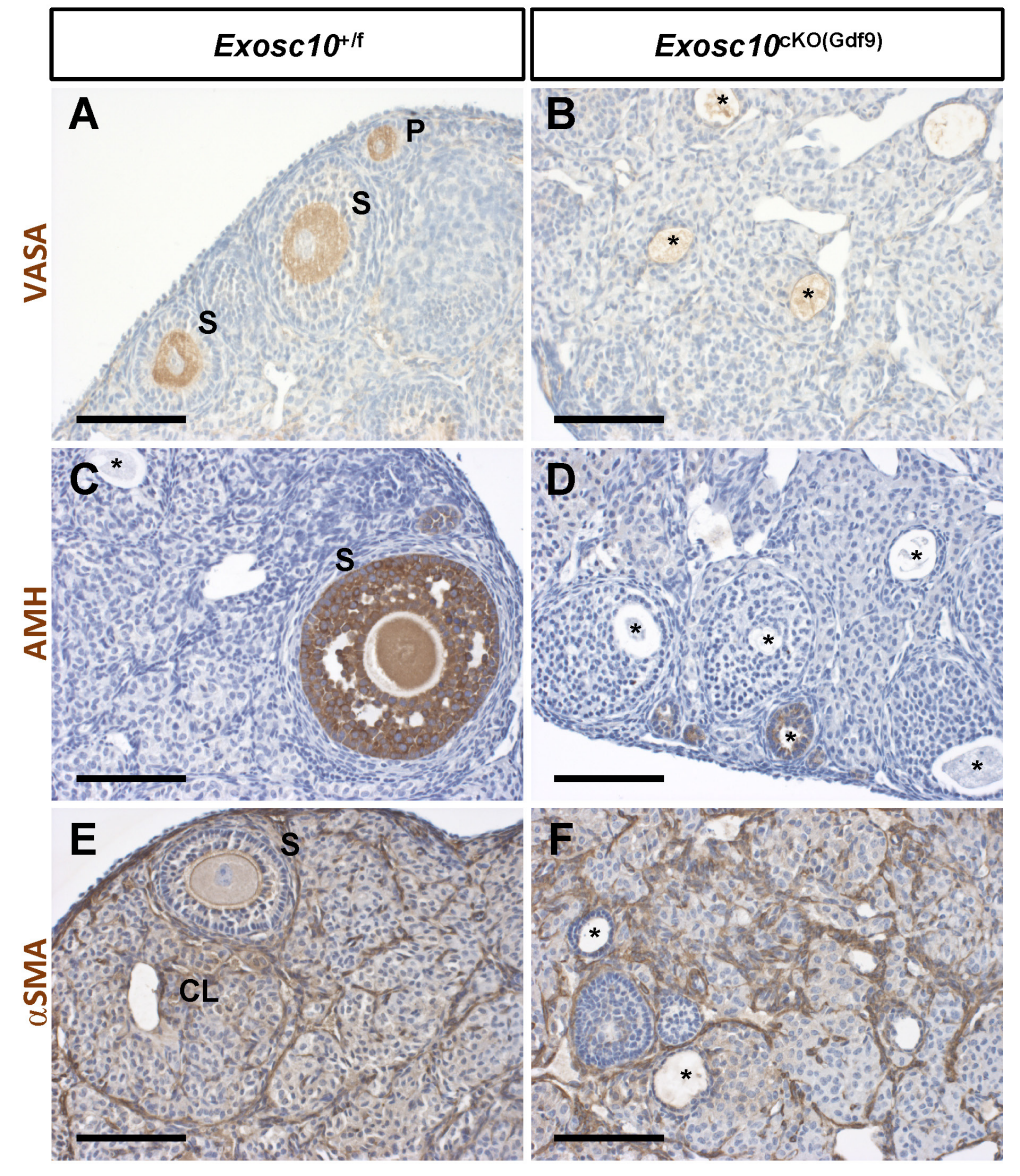
** Inactivation of *Exosc10* in oocytes leads to a disorganization of the ovarian tissue.** Immunohistochemistry on ovary sections from 8-week-old female mice using markers for germ cells (VASA in **A** and **B**), granulosa cells (AMH in **C** and **D**) and smooth muscle cells of the theca layer (αSMA in **E** and **F**). Degenerated oocytes are marked with an asterisk. P, Primary follicle; S, Secondary follicle; CL, Corpus Luteum. Scale bars: 100 µm.

Since folliculogenesis and the overall organization of the ovary were disrupted in *Exosc10*^cKO(Gdf9)^ mice, the identity of germ cells and somatic cells in 8-week-old ovaries was examined by immunohistochemistry using different ovarian cell-type markers (Fig. 3). VASA (DDX4), a cytoplasmic marker of germ cells, was expressed in germ cells from different classes of follicles in control ovaries (Fig. 3A). However, in *Exosc10*^cKO(Gdf9)^ ovaries, VASA staining was observed in degenerated oocytes located in undefined structures (Fig. 3B). AMH, a granulosa cell marker of growing follicles, was expressed in primary, secondary and small antral follicles in controls (Fig. 3C). In mutant ovaries, AMH expression was detectable exclusively up to the secondary follicle stage, indicating that folliculogenesis is perturbed or arrested at the secondary stage (Fig. 3D). The external theca cell marker, αSMA, revealed the outline of the growing follicles and the infiltration of the theca cells into the corpora lutea in control ovaries (Fig. 3E). In *Exosc10*^cKO(Gdf9)^ ovaries, the use of αSMA revealed disorganized ovarian tissue due to the absence of oocytes and corpora lutea (Fig. 3F). At six months, *Exosc10*^cKO(Gdf9)^ ovaries were significantly smaller than those of controls (ovary/body weight ratio of 43.0 ± 4.6 mg/g and n=6 for the controls versus 15.0 ± 4.3 mg/g and n=5 for the mutants; Fig. S2A). Histological sections of these ovaries revealed that they contained cyst-like structures and that the stroma compartment was composed of an undetermined somatic cell type (Fig. S2C-D). Collectively, our data show that loss of EXOSC10 in oocytes leads to a rapid depletion of the ovarian reserve and a disorganization of the ovarian tissue.

### Impaired oocytes development and maturation in* Exosc10*^cKO(Gdf9)^ female mice results in embryonic development arrest

**Figure 4:**
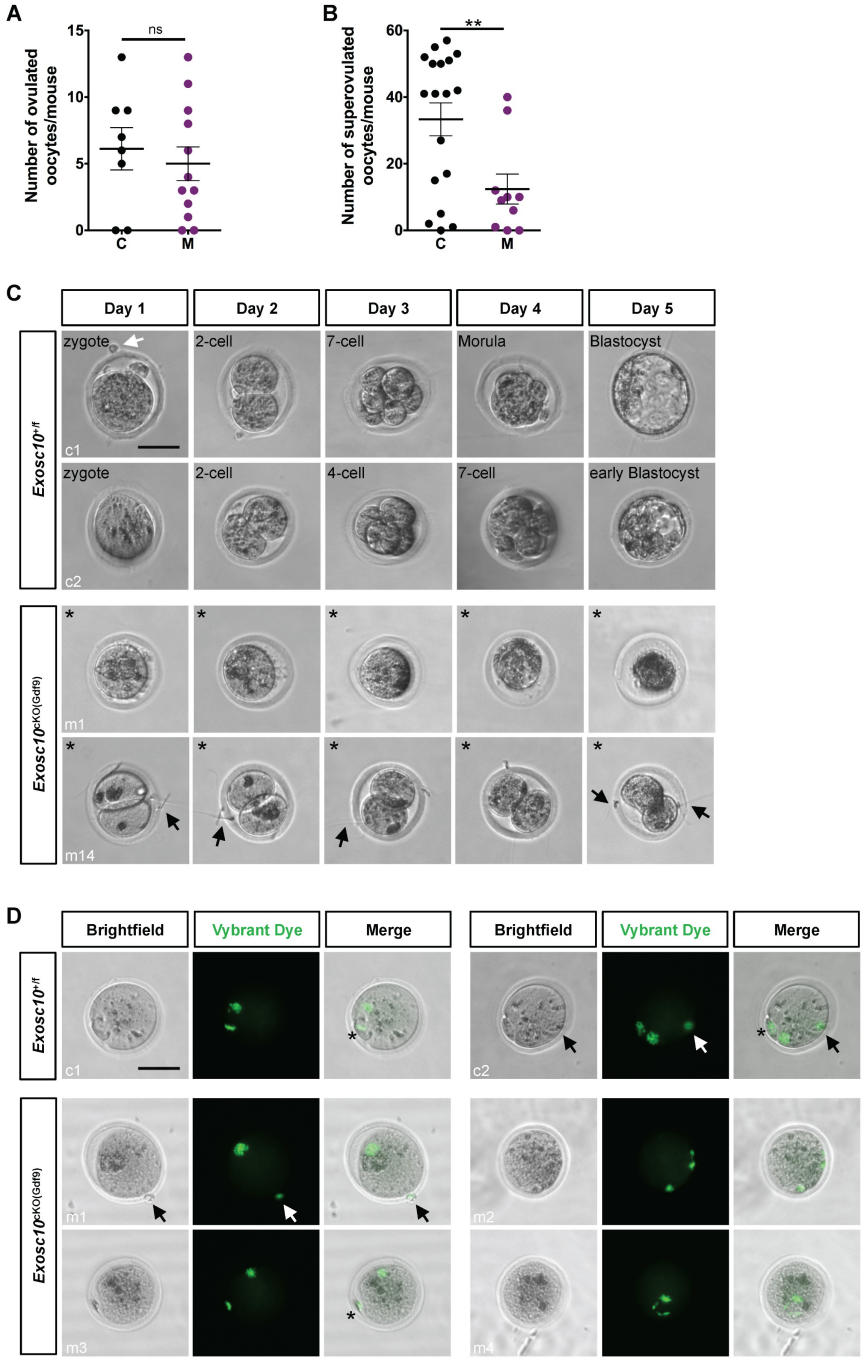
***Exosc10*^cKO(Gdf9)^ mice are sterile and ovulate abnormal oocytes.** (**A**) Ovulated oocytes collected from 6 to 8 week-old control and *Exosc10*^cKO(Gdf9)^ mice at PGD1. (**B**) Oocytes collected from 3 to 4 week-old control and *Exosc10*^cKO(Gdf9)^ mice after superovulation. (**C**) 6 to 8 week-old control and *Exosc10*^cKO(Gdf9)^ mice were mated with stud wild type males and oviducts were collected and flushed at gestation day 1 (= day 1 of culture). After treatment with hyaluronidase, expected zygotes were cultured in EmbryoMax Advanced KSOM medium for five days. Two representative control embryos (c1 and c2) showed different embryonic developmental stages as indicated on the brightfield images. Two representative non viable mutant oocytes are shown (m1 and m14). For m14, the oocyte presents an enlarged polar body. Black asterisks denote degenerated mutant oocytes. Black and white arrows point to spermatozoa and remaining follicular cells, respectively. Scale bar: 50 µm. (**D**) Oocytes were collected from superovulated females and stained for DNA content with Vybrant Dye. The green fluorescent signal highlights both nucleus and polar body from the oocyte. Black and white arrows point to remaining follicular cells. Polar bodies are represented by black asterisks. Scale bars: 50 µm.

Since folliculogenesis and oogenesis were severely impaired in 8-week-old *Exosc10*^cKO(Gdf9)^ female mice, we asked whether ovulation and embryonic development occur in younger mutant female mice. Indeed, in 6-week-old mutant ovaries, folliculogenesis appeared to be less affected than in older mice (Fig. 2F, G). Therefore, we mated 6-8-week-old control and *Exosc10*^cKO(Gdf9)^ female mice with stud C57BL/6NRj males and collected oviducts at gestational day 1 (GD1). Putative zygotes were flushed from the oviduct and cultured in EmbryoMAX Advanced KSOM medium for 5 days. In controls and mutants, 6.1 ± 1.6 (SEM, n=8) and 5.5 ± 1.3 (n=12) eggs were collected respectively (Fig. 4A). This result suggests that ovulation occurred in *Exosc10*^cKO(Gdf9)^ mice under natural conditions. Interestingly, after superovulation, the number of collected oocytes from mutants reached only one third of the collected oocytes from controls (Fig. 4B). This result reflects the depletion of follicles in mutant ovaries. Although control zygotes developed to the blastocyst stage after five days of culture, mutant eggs were not viable and degenerated (Fig. 4C and Fig. S3). We observed that 35% of the mutants exhibited an enlarged polar body, which could be the consequence of an abnormal first meiotic division (see m5,6,7,13,14; Fig. 4C and Fig. S3). Oocytes obtained after superovulation were analyzed for their DNA content in both nucleus and polar body using Vybrant Dye (Fig. 4D). While all controls displayed a normal first polar body extrusion, in mutants we observed that polar bodies were lacking (Fig. 4D, m1,2,4) or degenerated (Fig. 4D, m3). Moreover, the genomic DNA appeared to be fragmented (Fig. 4D, m2 and m4). Taken together, these results suggest that 6-week-old *Exosc10*^cKO(Gdf9)^ female mice can ovulate but inactivation of *Exosc10* impairs oocyte quality and subsequently proper embryo development.

### *Exosc10* oocyte specific inactivation affects essential cellular and reproductive processes in *Exosc10*^cKO(Gdf9)^ ovaries

**Figure 5:**
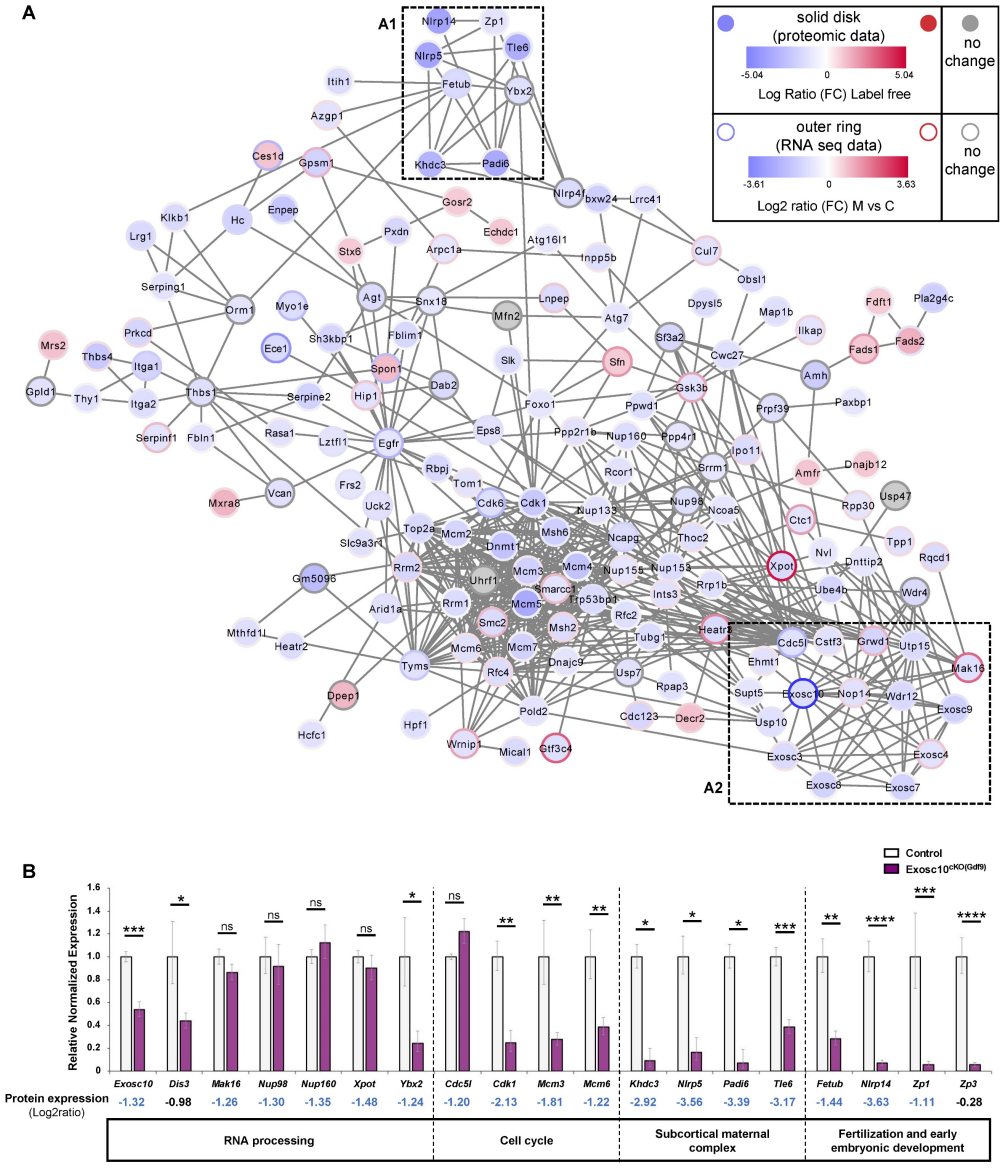
(**A**) **Cytoscape representation of a STRING network interaction (functional associations or physical interactions, protein-chemical pairs, and PubMed text mining) from the list of label free differentially detected proteins.** Solid disks correspond to proteomic data and the outer rings stand for the imported RNA seq data. The color intensity is related to the expression level (down in blue, up in red and no change in grey). A *reproduction sub-network* (box A1) clusters down-regulated proteins very important for the reproductive process. An *exosome sub-network* containing EXOSC10 and its partners is represented in the box A2. Transcriptomic data used were published by Wu and Dean, 2020. (**B**) Quantitative PCR analysis. Most of the down-regulated proteins in *Exosc10*^cKO(Gdf9)^ ovaries are also down-regulated at the RNA level. Expression values are represented as relative to the control group. Comparison with proteomic analysis; Log2 ratio values (mutant (M) vs control (C)) are indicated. *Exosc10*^+/f^ *and Exosc10*^cKO(Gdf9)^, n= 6. One-way ANOVA, ns : not significant, *p<0.05, **p<0.01, ***p<0.001, ****p<0.0001.

As part of the RNA exosome, EXOSC10 is known to be a key factor in the processing, modification and degradation of RNAs 23,24. It is also involved in rRNA processing 8,25. Recently, the analysis of our *Exosc10* KO mice suggested that EXOSC10, which is expressed at the periphery of NPBs, might play an important role in the rRNA maturation and hence in the ribosome and protein production 10. Therefore, to better understand the mechanism of follicular depletion in *Exosc10*^cKO(Gdf9)^ ovaries, we carried out a protein profiling analysis. Proteins were extracted from 8 to 10-week-old control and *Exosc10*^cKO(Gdf9)^ ovaries and further analyzed by mass spectrometry. Proteomic analysis revealed that in a total of 4732 proteins identified, 249 proteins were differentially detected (218 down- and 31 up-regulated) (Table S2 and S3, respectively) between control and mutant ovaries whereas the expression of 4483 proteins did not show any significant variation (Table S4). We first confirmed that, as expected, EXOSC10 expression is decreased in mutant ovaries (Table S2). Using Cytoscape software, we displayed exclusively the proteins included in a network (Fig. 5A). To establish a link between RNA and protein expression, the single cell RNA profiling data from Wu and Dean 11 was integrated in the graphic display presented in Fig. 5A. Among these entities, 40 % show consistent data in proteomic and transcriptomic analyses. Forty five percent show opposite RNA and protein expression profiles. This discrepancy could be explained by a difference in the design of our experiments (whole ovary for the proteomic experiment versus isolated oocytes for the RNA-Seq data). We identify sub-networks and pathways impaired by *Exosc10* invalidation in the oocytes that are comprised of proteins known to have essential roles in oocyte maturation and early embryogenesis. A *reproduction sub-network* (Fig. 5A1 black box and Table S5) shows decreased expression of several members of the subcortical maternal complex (SCMC) including NLRP5/MATER, PADI6, TLE6 and KHDC3/FILIA proteins (dark blue). This complex is essential for zygotes to progress beyond the first embryonic cell divisions and it is important for the symmetric divisions of zygotes by regulating the formation and positioning of the mitotic spindle 26,27. The other proteins clustered with the SCMC complex have essential roles in oocyte function: we observe decreased expression of FETUB (a protease inhibitor required for egg fertilization) 28; YBX2, (a major component of the messenger ribonucleoprotein particles (mRNP) whose genetic deletion leads to female infertility) 29; ZP1 (which ensures the structural integrity of the zona pellucida) 30 and NLRP14 (a pivotal regulator in primordial germ cell-like cell differentiation) 31 (Fig. 5A1 and Table S5).

Next, we confirmed by qPCR that expression of mRNAs corresponding to *Khdc3*, *Nlrp5*, *Padi6*, *Tle6* (from the SCMC) as well as *Fetub*, *Nlrp14*, and *Zp1* that are essential for the reproductive process was also downregulated in *Exosc10*^cKO(Gdf9)^ ovaries (Fig. 5B and Table S5). In addition, we analyzed the expression level of *Zp3*, another member of the Zp family and a key component of the zona pellucida. Although this protein was not considered as differentially regulated by mass spectrometry, expression of its mRNA was significantly decreased in mutant ovaries (Fig. 5B and Table S4).

Furthermore, we identified an *RNA exosome sub-network* (Fig. 5A2 red box and Table S6). As we could anticipate, core subunits of the RNA exosome complex (EXOSC3, 4, 7, 8, 9) together with EXOSC10 as well as interactors involved in biological processes regulated by EXOSC10 are linked and less expressed in *Exosc10*^cKO(Gdf9)^ ovaries (Fig. 5A2 and Table S2, S6). Using a qPCR assay, we showed that *Dis3* is down-regulated in *Exosc10*^cKO(Gdf9)^ ovaries; we note that protein profiling data showed a moderate decrease of the protein (Fig. 5B and Table S4).

Additional proteins important for ribosome biogenesis that interact with EXOSC10 were found to be expressed at lower levels in *Exosc10*^cKO(Gdf9)^ ovaries (Fig. 5A and Table S6). This group includes UTP15 (part of the small subunit processome involved in the nucleolar processing of the pre-18S rRNA) 32, WDR12 (part of the PeBoW complex is required for the maturation of the 60S subunit) 33, and NOP14 (involved in the nuclear export of the 40S pre-ribosomal subunit in the cytoplasm) 32. The nucleoporins NUP98 and NUP160 involved in mRNA export 34 were found to be downregulated at the protein level but not at the mRNA level in *Exosc10*^cKO(Gdf9)^ ovaries (Fig. 5A,B and Table S2). EXOSC10 interacts with CDC5L (involved in the cell cycle and oocyte meiosis) 35 and GRWD1 (a ribosomal protein involved in oncogenesis) 36 whose expression is decreased in mutant ovaries. Expression of *Cdc5l* was unchanged in *Exosc10*^cKO(Gdf9)^ ovaries, but other downregulated proteins involved in the cell cycle, like CDK1, and all the components of the MCM complex, were significantly downregulated at the mRNA level (Fig. 5A,B).

We note that three proteins (MAK16, GRWD1 and EXOSC4) of the *RNA exosome sub-network* (Fig. 5A2 and Table S6) as well as XPOT, an exportin involved in the nuclear export of tRNAs 37 were expressed at lower levels in mutant ovaries while their corresponding genes were up-regulated in *Exosc10*^f/f^; *Zp3-*Cre oocytes (Fig. 5A, blue disk with a red circle, 11). We therefore analyzed by qPCR the RNA expression level of *Mak16* and *Xpot* and did not find any significative differences between control and mutant ovaries (Fig. 5B). This discrepancy could be explained by the use of different cKO mice for *Exosc10* and/or different samples (whole ovaries in our study versus oocytes in 11 study). It is also possible that a compensatory effect increases the quantity of proteins by activating genes that encode them.

Finally, we performed a Gene Ontology (GO) term enrichment and found that *Exosc10* mutant ovaries express lower levels of proteins involved in well-known processes regulated by EXOSC10 (*i.e.,* DNA replication and repair process, the rRNA and mRNA maturation and surveillance, nucleocytoplasmic transport, rRNA and snoRNA 3' end processing) (Table S7).

## Discussion

The present study demonstrates the essential role of *Exosc10* in early oogenesis. Until six weeks of age, *Exosc10*^cKO(Gdf9*)*^ mice displayed a normal onset of puberty with unperturbed estrus cycles. The ovaries and follicular distribution are identical between mutant and control mice. Between six and eight weeks, *Exosc10*^cKO(Gdf9)^ mice start having irregular estrus cycles and by eight weeks the oocytes from *Exosc10*^cKO(Gdf9)^ mice are depleted. The collection of putative fertilized oocytes from 6-week-old *Exosc10*^cKO(Gdf9)^ mouse oviducts at GD1 revealed that all of them exhibited a poor morphology leading to an absence of embryo development in culture experiments. Moreover, some oocytes contain a large first polar body, which could be a consequence of a defective first meiotic division.

During folliculogenesis, the oocyte accumulates maternal RNAs and proteins, which play a key role in the oocyte-to-embryo transition. After fertilization, the amount of RNAs and proteins in the oocyte decreases rapidly until the zygotic genome activation occurs at the 2-cell stage in mouse; then, the survival of the embryo beyond the 2-cell stage depends on the balance between stability and degradation of maternal RNA and rRNA production (for review, see 38-40). In *Exosc10* knockout mouse embryos generated from *Exosc10*^+/-^ intercrosses, maternal accumulation of wild type *Exosc10* mRNA allows the embryos to develop beyond the 2-cell stage and the developmental arrest observed at the 8-cell/morula transition is likely related to a disruption of the nucleolar rRNA processing 10. In our *Exosc10*^cKO(Gdf9)^ mice, the poor quality and morphology of the ovulated oocytes as well as the inability of supposedly fertilized oocytes to develop into an embryo, suggest that *Exosc10* is essential for the maturation of the oocyte and that accumulation of *Exosc10* maternal mRNA and protein is a prerequisite for the first divisions of the embryo.

Interestingly, in a recent study using the *Zp3-*Cre mice to inactivate *Exosc10* in oocytes 11, the *Zp3-*Cre; *Exosc10*^f/-^ mice displayed a less severe phenotype since they were subfertile 11. This difference could be explained by the presence of functional oocytes in which *Exosc10* was not deleted due to incomplete Cre activity. The *Exosc10*^cKO(Gdf9)^ mouse model shows greater penetrance of the phenotype since it causes severe infertility, which could be explained by earlier inactivation of *Exosc10* by the Cre recombinase during oogenesis. As observed by Lan et al., the Cre recombinase is expressed earlier in *Gdf9-*iCre mice than in *Zp3-*Cre mice during mouse development (*Gdf9*, postnatal day 3 versus *Zp3*, postnatal day 5), and therefore it is active earlier during folliculogenesis (*Gdf9*, primordial stage versus *Zp3*, primary stage) 12. Given that accumulation of maternal RNAs and proteins starts at the primordial follicular stage 41, it is conceivable that in *Zp3-*Cre mice a large proportion of oocytes contain maternal RNAs/proteins, which enables the embryo to develop beyond the 2-cell stage until birth. Nevertheless, in agreement with our results, Wu and Dean reported for *Zp3-*Cre; *Exosc10*^f/-^ oocytes a decrease of activated CDK1 protein blocking the nucleosome disassembly, altered endomembrane components and an impaired meiotic cell cycle 11. The observation of enlarged polar body in about 30 % of *Exosc10*^cKO(Gdf9)^ oocytes collected at GD1 suggest that the first meiotic division is altered. During meiosis, after spindle formation in the center of the oocyte, the spindle should migrate towards the cortex where the extrusion of the first polar body occurs 42. This asymmetric cell division is crucial for the oocyte, as it allows it to retain the maximum amount of maternal RNA/protein material. In the mouse, any disruption of the MOS/MAPK pathway alters spindle migration, which perturbs asymmetric division and therefore the formation of a large polar body 43. In human, similar abnormalities of the polar body extrusion have been found in patients bearing mutations in TUBB8, PATL2 and MOS genes 44-46.

Previous transcriptomic data analyses indicated that *Exosc10* transcripts were very abundant in mouse oocytes and in early embryos 47,48. We observed that EXOSC10 is highly expressed in the nucleus of oocytes at every follicle stage (Fig. 1) as well as in oocytes from 14.5 days post-conception onwards. Recently, we showed that EXOSC10 accumulates at the periphery of the NPBs during early embryogenesis 10. Since EXOSC10 processes rRNA, we investigated to what level protein expression was affected in *Exosc10*^cKO(Gdf9)^ mice. Our proteomic analyses performed on 8-week-old ovaries revealed that proteins involved in different phases of the cell cycle were deregulated, which explains the meiotic phenotype. The increase of SFN (14-3-3-sigma) expression associated with a decrease of CDK1 expression might amplify meiotic arrest. Indeed, SFN dimers are known to sequester the complex cyclin B1 (CCNB1)/ CDK1 (Cdc2) in the cytosol, thus preventing the latter from phosphorylating the nuclear factors essential for the G2/M transition 49.

We also identified several proteins with essential roles in oocyte maturation and four components of the subcortical maternal complex (SCMC; MATER/NLRP5, TLE6, FILIA/KHDC3 and PADI6) that were expressed at very low levels in mutant ovaries as compared to controls. The SCMC is involved in the formation of meiotic and mitotic spindle, and symmetric cell divisions. The reduced expression of these proteins in *Exosc10*^cKO(Gdf9)^ mice could explain the alteration of the polar body extrusion phase, which then leads to the formation of a large polar body.

Our results show that *Exosc10* is essential for the maturation of the oocyte and for proper completion of meiosis. Interestingly, the phenotype of the *Exosc10*^cKO(Gdf9)^ female mice demonstrates that deletion of *Exosc10* in oocytes leads to a condition similar to primary ovarian insufficiency (POI) since lacking EXOSC10 activity leads to rapid depletion of the oocytes and to irregular estrus cycles. POI affects approximately 1% of women under 40 years and leads to female infertility 50. POI is characterized by premature depletion of ovarian follicles leading clinically to amenorrhea and elevated gonadotropins plasma levels. Infertility, defined by the inability for a couple to establish a clinical pregnancy after 12 months of regular and unprotected intercourse with an healthy partner, is estimated to affect between 8 and 12% of reproductive-aged couples worldwide 51. Female factors represent 35% of infertility causes which can affect all steps of reproductive female aspects like ovarian development, hormone signaling, oocyte maturation, fertilization competence, preimplantation development, implantation, fetal growth and ovarian reserve maintenance. It would be interesting to know whether *Exosc10* heterozygous or homozygous mutations exist in the human germ line and to further analyze if affected women suffer from a POI or present defects in reproductive capacities.

## Supplementary Material

Supplementary figures and tables.

## Figures and Tables

**Table 1:**
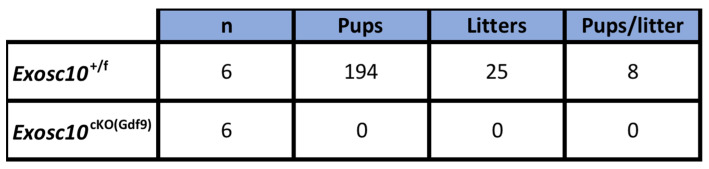
Reproductive performance of *Exosc10*^+/f^ and *Exosc10*^cKO(Gdf9)^ mice

Eight-week-old female mice were mated with a stud male for a period of six months. Litters and pups were counted.
