# Supplementary material for: Inactivation of Exosc10 in the oocyte impairs oocyte development and maturation, leading to a depletion of the ovarian reserve in mice
Source: Int J Biol Sci. 2023 Jan 31;19(4):1080–93. doi: 10.7150/ijbs.72889 (PMC10008699; doi:10.7150/ijbs.72889)
Supplement: Supplementary file 1 — Supplementary figures and tables. [file ijbsv19p1080s1.pdf]

Inactivation of *Exosc10* in the oocyte impairs oocyte development and maturation, leading to a depletion of the ovarian reserve in mice.

Leïla Demini[1], Christine Kervarrec[1,*], Laëtitia Guillot[1,2,*], Emmanuelle Com[1,2], Régis Lavigne[1,2], Pierre-Yves Kernanec[1], Michael Primig[1], Charles Pineau[1,2], Fabrice G. Petit[1,§,‡], Soazik P. Jamin[1,§,‡].

1 Univ Rennes, Inserm, EHESP, Irset (Institut de recherche en santé, environnement et travail) – UMR_S 1085, F-35000 Rennes, France

2 Univ Rennes, CNRS, Inserm, Biosit UAR 3480 US 018, Protim core facility, F-35000 Rennes, France

* These authors contributed equally

§ These authors contributed equally

‡ Corresponding authors : soazik.jamin@inserm.fr ; fabrice.petit@inserm.fr

Keywords: *Exosc10*, oogenesis, follicular development, ovary

Supplementary information

Supplementary Figures S1-S3
Supplementary Tables S1-S7

Figure S1

A

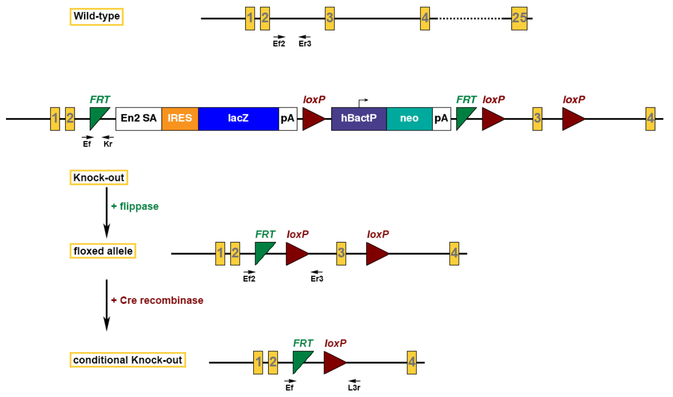

C

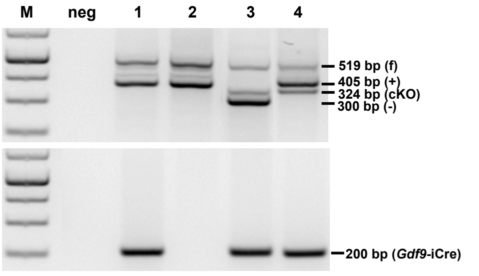

B

Primer name	DNA Sequence
Ef	5'-GAT GGA GCG AGC AAG CTT CTG-3'
Kr	5'-CCA ACA GCT TCC CCA CAA CGG-3'
Ef2	5'-TCT AAG CCT GAC AGC ATG AAT TGA ACC-3'
Er3	5'-GTG GTG TAG ACT TGT GAT ACT GC-3'
L3r	5'-GCA GGT CTC CAC TGA CAC TG-3'
Gdf9CRE-F	5'-GGC ATG CTT GAG GTC TGA TTA C-3'
Gdf9CRE-R	5'-CAG GTT TTG GTG CAC AGT CA-3'
VasaCRE-F	5'-CAC GTG CAG CCG TTT AAG CCG CGT-3'
VasaCRE-R	5'-TTC CCA TTC TAA ACA ACA CCC TGA-3'

Figure S1. (**A**) Schematic representing the wild type *Exosc10* allele composed of 25 exons (yellow squares) and knock-out, floxed and conditional knock-out alleles used in this study (see www.komp.orf/alleles.php#nonconditional-promoter-csd). The IRES-lacZ trapping cassette containing the En2 splice acceptor (En2 SA) allows for the inactivation of *Exosc10* gene function (knock-out). From the knock-out allele, a functional allele (flowed allele) is generated using the *flippase* (recognition sites. FRT, green triangles). The exon 3, flanked by two loxP sites (red triangles) can be deleted in the presence of a Cre recombinase. A frame shift mutation (conditional knock-out) leads to an inactivation of *Exosc10* gene. Annotated black arrows correspond to the primer sets used for genotyping. (**B**) Sequences of primers used for genotyping. (**C**) A representative genotyping PCR assay showing the results for a negative control (lane neg), and for samples from *Gdf9*-iCre*; Exosc10*$^{+/f}$ (lane 1), *Exosc10*$^{+/f}$ (lane 2), *Gdf9*-iCre*; Exosc10*$^{f/-}$ (lane 3, *Exosc10*$^{cKO(Gdf9)}$) and *Gdf9*-iCre*; Exosc10*$^{+/f}$ with an ectopic expression of Cre is shown (lane 4). The PCR amplicon is 519 base pair (bp) for the floxed allele (f), 405 bp for the wild type allele (+), 324 bp for the conditional knock-out allele (cKO) and 300 bp for the knock-out allele (-). M, DNA ladder.

Figure S2

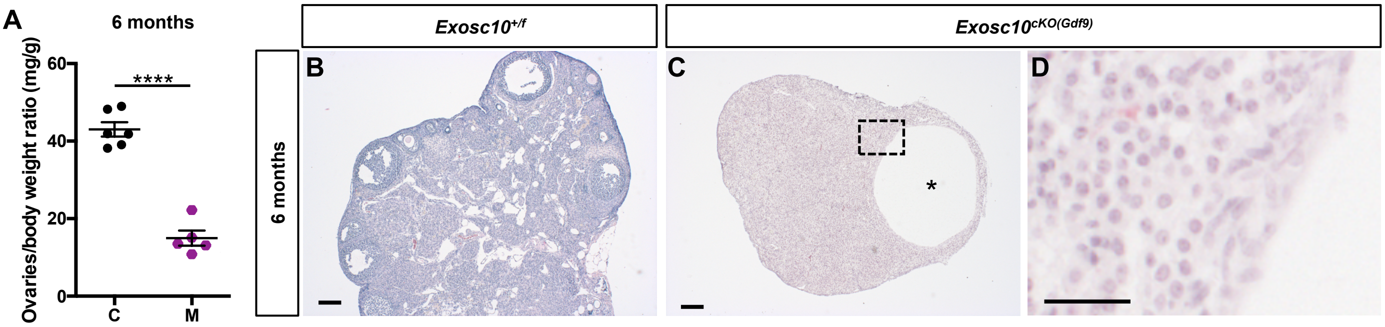

Figure S2. (**A**) Graph showing the ovaries/body weight ratio of 6-month-old mice. Control (C, n=6) and *Exosc10*$^{cKO(Gdf9)}$ (M, n=5). Two-tailed Student's test, ****p<0,0001. (**B-D**) Histological sections of 6-month-old ovaries stained by hematoxylin and eosin. Cyst-like structures are shown in *Exosc10*$^{cKO(Gdf9)}$ ovaries (asterisk in C). Scale bars: 200 µm. High magnifications (D) of the marked area in C. Scale bar: 50 µm.

Figure S3

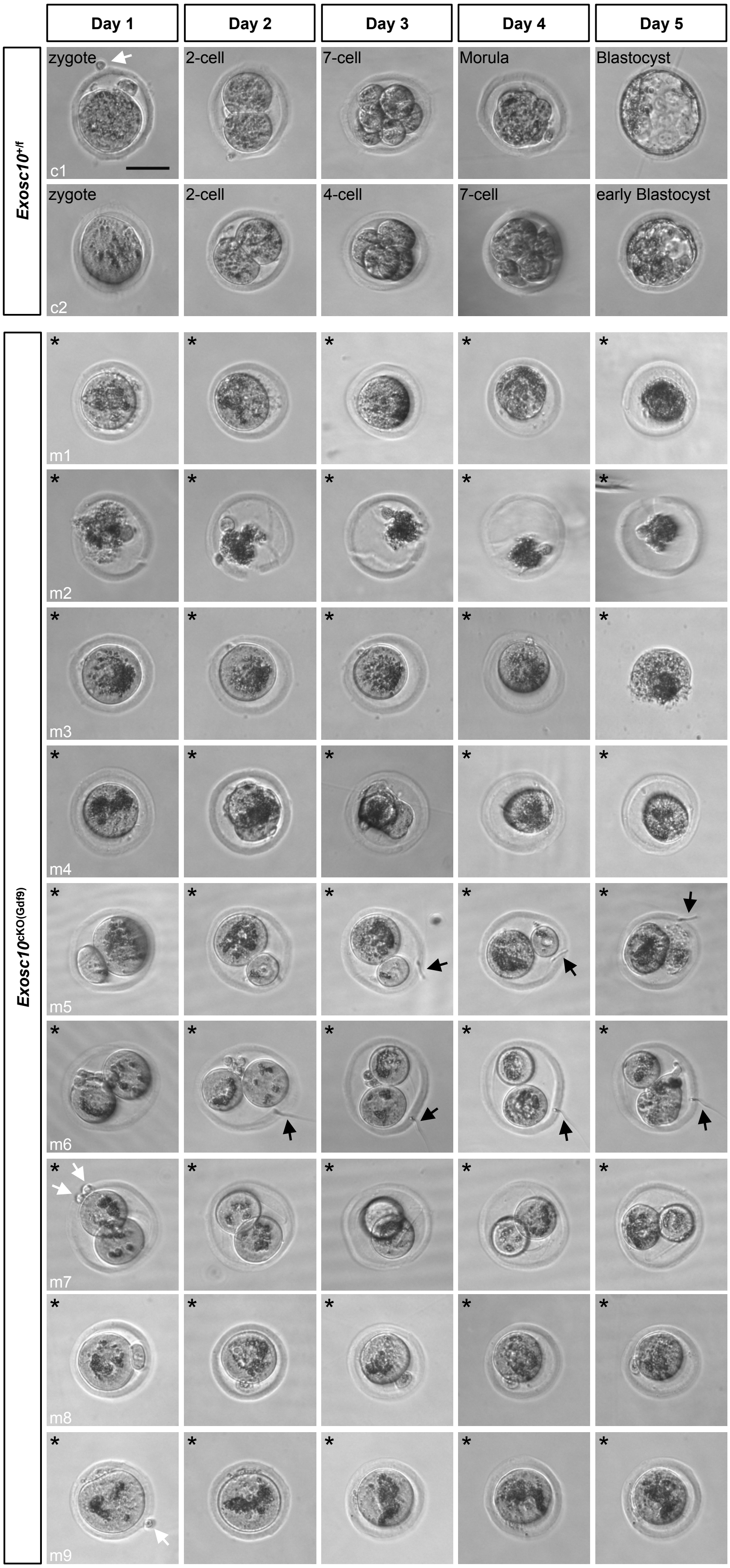

Figure S3. *Exosc10*$^{cKO(Gdf9)}$ mice are sterile and ovulate abnormal oocytes.
See complete figure legend on next page.

Figure S3

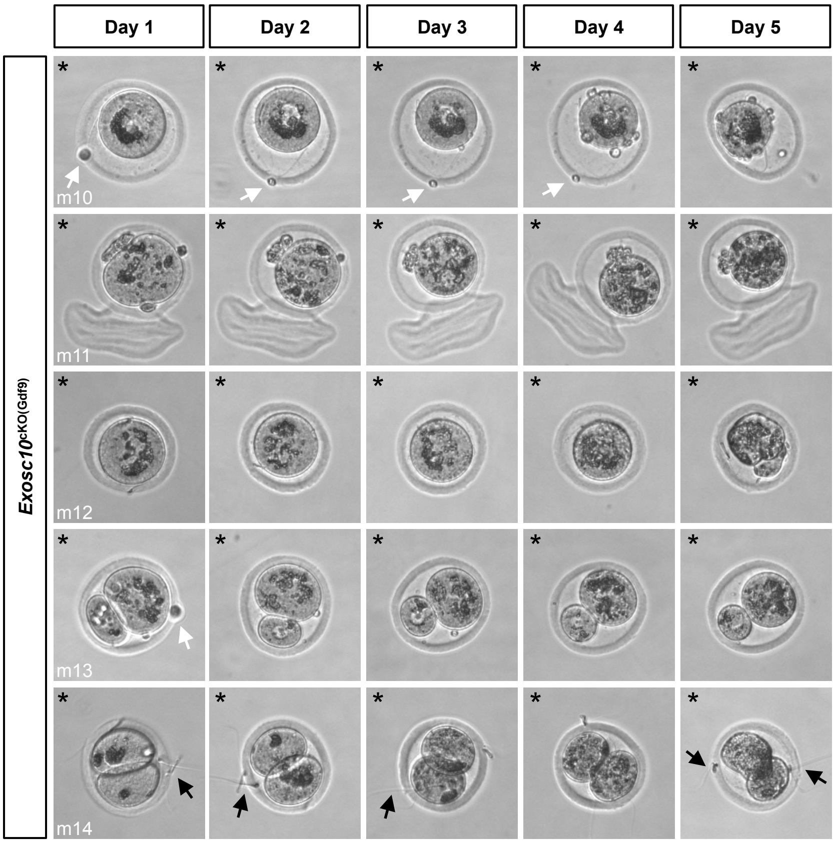

Figure S3. *Exosc10*$^{cKO(Gdf9)}$ mice are sterile and ovulate abnormal oocytes. 6 to 8 week-old control and *Exosc10*$^{cKO(Gdf9)}$ mice were mated with stud wild type males and oviducts were collected and flushed at gestation day 1 (= day 1 for the culture). After treatment with hyaluronidase, expected zygotes were cultured in EmbryoMax Advanced KSOM medium for 5 days. Photographs were taken every 24 hours (between 10:30am and 12:30pm). Two representative control embryos (c1 and c2) showed the different embryonic developmental stages as indicated on the brightfield images. Fourteen non viable mutant oocytes are shown (m1 to m14). Black asterisks denote degenerated mutant oocytes. Black and white arrows point to spermatozoa and remaining follicular cells, respectively. Scale bar: 50 µm.

Supplementary tables

Table S1

Gene	Gene name	NCBI referenc	Forward 5' - 3'	Reverse 5' - 3'	Size (bp
Cdc5l	*Cell division cycle 5-like protein*	NM_152810.2	GCAAGTTGTGCAGACTCCAA	TCTACCTGGGGTGGCATTAG	127
Cdk1	*Cyclin-dependent kinase 1*	NM_007659.4	ACAAAGGAACAATCAAACTGGC	AGCAACACTTCTGGAGATCG	115
Dis3	*Exosome complex exonuclease RRP44 isoform 1*	NM_028315.2	GCGATGTTGGAGAGAAGGAG	GGTCCACACTGCAAACACAC	177
Exosc10	*Exosome component 10*	NM_016699.3	CAGGGAGGATGAGAGCTACG	GAGGCTCCTTAGGCAGTTCC	60
Fetub	*Fetuin beta*	NM_021564.2	GACTCTGAAGTGCTGGCAGT	TTCTGTGCCTTCCTGCTGAG	194
Khdc3	*KH domain containing 3*	NM_025890.3	TACGTGGAACCTCGGCTACT	CAAATCTCAGCTCGCCTTTC	128
Mak16	*Mak16 homolog*	NM_026453.3	ACTGAAGCGACAGAGGAAGC	AGGCATGGATGGGGAAGTTG	176
Mcm3	*Minichromosome Maintenance Complex Component 3*	NM_008563.3	TGACCTGCTCTTCATCATGC	CTGTGGCCAGGATATCCACT	152
Mcm6	*Minichromosome Maintenance Complex Component 6*	NM_008567.2	TGAGAGAAACACGCTGGTTG	AAGGTCTTCAAGGCTCGACA	123
Nlrp5	*NLR family pyrin domain containing 5* (ou *Mater*)	NM_011860.3	CCTTGGGAATGCCTTGAGTA	CTGAAAAGGGCTGAGACCAG	108
Nlrp14	*NLR family pyrin domain containing 14*	NM_001002894.2	TCTGCAGAGCCTTGTGCTTA	CATTATCCGCCAGCTTGTTT	127
Nup98	*Nuclear pore complex protein 98*	NM_001287164.1	ATCAGGAGCCTGTGTCTGCT	CTCCTTTGGAAGGGATGTGA	150
Nup160	*Nuclear pore complex protein 160*	NM_021512.2	CCAACTACTGCATCCCTGGT	GATGCTGCCTGACAGAAACA	120
Padi6	*Peptidyl arginine deiminase 6*	NM_153106.2	GGACACTGTGGTCTGGAGGT	GGGACTTCTTGGTCAGGACA	126
RplP0	*60S acidic ribosomal protein P0*	NM_007475.5	ACTGGTCTAGGACCCGAGAAG	CTCCCACCTTGTCTCCAGTC	124
Rpl13a	*60S ribosomal protein L13a*	NM_009438.5	CATGAGGTCGGGTGGAAGTA	GCCTGTTTCCGTAACCTCAA	116
Tle6	*Transducin-like enhancer of split 6*	NM_053254.2	GGAACCTCAAAGGCCCTACC	ATGCTCCAGTGACACCTTGG	128
Xpot	*Exportin tRNA*	NM_001081056.2	TTCAAGATGCAGGTGTCCCC	GCACCTTGATTCGCGATCAC	200
Ybx2	*Y box protein 2*	NM_016875.3	AATCCAAGTCCTGGGCACAG	ACTCCGCAGAAACTTCCTGG	139
Zp1	*Zona pellucida 1*	NM_009580.2	GCCCAGGAGCACACAGTATT	CATTGGGTAGGACGGCTTGT	106
Zp3	*Zona pellucida 3*	NM_011776.1	CAATGTGAGCAGCCACCCTA	TCTCCTCCATCAGGCGAAGA	101

Table S1. Primer sequences used for the real time PCR

Table S2. Proteins down-regulated in mutant ovaries

EXOSC# are noted as EXOS#, EXOSC10 is noted EXOSX, NUP160 is noted NU160

UniprotKB Accession Number	Entry Name	Beta-binomial test	Log2 ratio (M vs C)
Q6B966	NAL14	8,2531E-05	-3,63
Q9R1M5	NALP5	1,4667E-05	-3,56
Q8K3V4	PADI6	1,3046E-05	-3,39
P49718	MCM5	7,7814E-06	-3,38
Q9WVB3	TLE6	1,1966E-05	-3,17
Q9CWU5	KHDC3	9,5966E-06	-2,93
Q8VDF2	UHRF1	6,2034E-05	-2,80
O35490	EHMT1	1,6493E-03	-2,63
Q61142	SPIN1	1,6246E-04	-2,49
P49717	MCM4	7,5944E-05	-2,38
P13864	DNMT1	2,4640E-02	-2,38
P16406	AMPE	1,4650E-04	-2,24
Q9Z1T2	TSP4	8,4495E-04	-2,14
P11440	CDK1	9,8451E-05	-2,14
P47226	TES	4,8469E-03	-2,07
P27106	MIS	2,9271E-04	-2,00
Q64GA5	PA24C	3,1319E-04	-1,93
Q3UHK6	TEN4	1,8847E-02	-1,93
P54276	MSH6	2,6072E-02	-1,85
Q9JHI7	EXOS9	1,1440E-03	-1,82
P25206	MCM3	2,6323E-04	-1,81
O35638	STAG2	5,9330E-02	-1,79
Q61881	MCM7	2,6537E-05	-1,77
Q6IR34	GPSM1	4,2466E-04	-1,77
Q07235	GDN	5,3651E-04	-1,77
Q9D753	EXOS8	1,5063E-03	-1,75
Q9D0M0	EXOS7	4,0330E-03	-1,74
P06684	CO5	4,4301E-03	-1,74
Q3V3R4	ITA1	8,1791E-03	-1,68
Q8R550	SH3K1	1,0973E-02	-1,68
Q810D6	GRWD1	1,1860E-02	-1,68
Q9ES00	UBE4B	1,8126E-02	-1,68
Q8BG60	TXNIP	2,8977E-02	-1,68
P97310	MCM2	3,1478E-04	-1,66
Q9DBD0	ICA	2,2327E-03	-1,64
P28867	KPCD	5,0743E-03	-1,63
Q8CII2	CD123	2,7373E-03	-1,60

Q62203	SF3A2	3,9554E-03	-1,60
Q6P5D8	SMHD1	7,3264E-02	-1,60
P11157	RIR2	1,1328E-03	-1,58
P31266	SUH	4,1538E-03	-1,58
Q9EPR5	SORC2	1,3238E-02	-1,58
Q3UQ28	PXDN	2,4355E-02	-1,58
P19137	LAMA1	9,5883E-02	-1,58
Q9D3E6	STAG1	8,2434E-02	-1,54
Q01320	TOP2A	1,1025E-02	-1,53
Q9JJA4	WDR12	1,2482E-03	-1,53
Q71FD7	FBLI1	3,7869E-03	-1,53
P70399	TP53B	6,1222E-03	-1,49
Q8VE19	MIO	2,0440E-03	-1,49
P26262	KLKB1	2,3103E-03	-1,49
Q9CRT8	XPOT	3,1560E-03	-1,49
Q8K2I4	MANBA	2,1937E-02	-1,49
Q9EP82	WDR4	2,6616E-02	-1,49
P98078	DAB2	6,0649E-03	-1,46
Q8VDP3	MICA1	6,3052E-03	-1,44
Q4PZA2	ECE1	1,4295E-02	-1,44
Q8C7V3	UTP15	2,0386E-02	-1,44
Q9QXC1	FETUB	2,3427E-02	-1,44
Q8VBT9	ASPC1	4,3108E-02	-1,44
Q6ZQ73	CAND2	1,1306E-02	-1,43
Q8BQM4	HEAT3	2,4135E-02	-1,42
P43247	MSH2	1,5348E-03	-1,40
P07742	RIR1	1,0932E-02	-1,39
D3YYU8	OBSL1	1,2528E-02	-1,39
Q8C129	LCAP	1,8265E-02	-1,39
Q5U4C1	GASP1	3,4743E-03	-1,38
P11859	ANGT	3,4743E-03	-1,38
P83887	TBG1	3,5343E-03	-1,38
Q9JKY0	CNOT9	3,5343E-03	-1,38
Q9EQF6	DPYL5	3,8780E-03	-1,38
Q8K2V6	IPO11	4,1005E-03	-1,38
E9PWG6	E9PWG6	5,5244E-03	-1,38
Q62393	TPD52	6,0807E-03	-1,38
Q8CG48	SMC2	1,2046E-02	-1,38
Q7TQK4	EXOS3	1,4346E-02	-1,38
O54988	SLK	2,1661E-02	-1,38
B9EJR8	DAAF5	2,4798E-02	-1,38
Q8CJ26	NRADD	2,6466E-02	-1,38
Q66X05	NAL4F	3,5456E-02	-1,38

Q61702	ITIH1	1,3387E-03	-1,36
Q8CEC6	PPWD1	1,0246E-02	-1,36
Q9Z0W3	NU160	1,1276E-02	-1,36
E9Q634	MYO1E	1,4313E-02	-1,36
Q99J62	RFC4	2,4312E-02	-1,36
P56960	EXOSX	1,1518E-02	-1,32
Q62469	ITA2	5,7119E-03	-1,30
Q6PFD9	NUP98	2,7533E-03	-1,30
P14873	MAP1B	1,1107E-03	-1,28
Q64261	CDK6	1,7452E-02	-1,27
Q8BGS0	MAK16	1,1382E-02	-1,26
Q8K337	I5P2	6,3457E-03	-1,26
Q91YK2	RRP1B	6,3511E-03	-1,26
Q9WVT6	CAH14	6,3511E-03	-1,26
Q8CFE2	HPF1	6,3513E-03	-1,26
P35441	TSP1	6,6566E-03	-1,26
Q3V3R1	C1TM	7,3924E-03	-1,26
Q9JJ28	FLII	1,1628E-02	-1,26
Q8K2Z2	PRP39	1,1628E-02	-1,26
Q9ER88	RT29	1,4332E-02	-1,26
Q9WUK4	RFC2	1,6930E-02	-1,26
Q3TYX3	SMYD5	1,6930E-02	-1,26
Q8BR92	PALM2	1,7776E-02	-1,26
Q3TKY6	CWC27	1,7963E-02	-1,26
Q8K1C9	LRC41	1,8168E-02	-1,26
Q8BLY1	SMOC1	2,2014E-02	-1,26
Q8BMQ2	TF3C4	2,2406E-02	-1,26
Q6NWW9	FND3B	4,0098E-02	-1,26
B1AZI6	THOC2	4,5779E-02	-1,26
Q3UIR3	DTX3L	5,6646E-02	-1,26
Q9D071	MMS19	7,8432E-02	-1,26
Q62383	SPT6H	9,4671E-02	-1,26
Q9Z2C8	YBX2	2,0973E-02	-1,24
P97311	MCM6	2,6146E-04	-1,22
Q6A068	CDC5L	1,3684E-02	-1,20
P70441	NHRF1	2,0336E-02	-1,20
Q8BY87	UBP47	2,0734E-02	-1,20
P52479	USP10	2,4809E-02	-1,19
Q99LI7	CSTF3	3,1518E-02	-1,19
Q9D706	RPAP3	2,8189E-02	-1,18
Q8CGC4	LS14B	2,9639E-02	-1,18
Q61191	HCFC1	9,5946E-03	-1,17
Q8R0G9	NU133	1,1758E-02	-1,16

Q6A4J8	UBP7	1,5263E-02	-1,16
Q91XU0	WRIP1	2,7555E-02	-1,16
Q64695	EPCR	2,7555E-02	-1,16
O89023	TPP1	2,7555E-02	-1,16
P01831	THY1	2,7555E-02	-1,16
Q08509	EPS8	2,7555E-02	-1,16
P97770	THUM3	2,7555E-02	-1,16
Q99KN2	CIAO1	3,0390E-02	-1,16
Q9Z321	TOP3B	7,2594E-02	-1,16
Q7TNP2	2AAB	1,3036E-02	-1,16
Q9D964	GATM	4,4766E-03	-1,15
Q7TPD0	INT3	4,3835E-02	-1,15
Q08879	FBLN1	1,2627E-02	-1,15
Q99PM9	UCK2	1,1458E-02	-1,14
P07607	TYSY	1,1458E-02	-1,14
Q8VE73	CUL7	1,3547E-02	-1,14
Q811S7	UBIP1	1,8064E-02	-1,14
Q6ZQ58	LARP1	1,8999E-02	-1,14
Q8R3N1	NOP14	1,8999E-02	-1,14
Q3TMX7	QSOX2	1,9810E-02	-1,14
Q8CFE3	RCOR1	1,9875E-02	-1,14
A2BH40	ARI1A	3,1600E-02	-1,14
Q5DW34	EHMT1	3,1665E-02	-1,14
O70362	PHLD	4,6016E-02	-1,14
Q9Z2L6	MINP1	7,2375E-02	-1,14
A6H5Y3	METH	7,2375E-02	-1,14
Q8K4G1	LTBP4	7,6323E-02	-1,14
O88508	DNM3A	7,8043E-02	-1,14
P54103	DNJC2	7,9875E-02	-1,14
Q9JL35	HMGN5	8,3083E-02	-1,14
Q8VHY0	CSPG4	9,3194E-02	-1,14
Q62059	CSPG2	4,0572E-02	-1,14
P97290	IC1	2,4790E-02	-1,13
Q62005	ZP1	4,7864E-02	-1,12
Q9D0M1	KPRA	2,2304E-02	-1,10
Q8VD75	HIP1	2,4653E-02	-1,09
O08738	CASP6	3,2764E-02	-1,09
Q9WV60	GSK3B	3,3826E-02	-1,09
Q921I9	EXOS4	3,4105E-02	-1,09
Q64726	ZA2G	4,1148E-02	-1,09
P24788	CD11B	4,3756E-02	-1,09
O88746	TOM1	4,5759E-02	-1,09
P97496	SMRC1	4,2541E-02	-1,08

O08532	CA2D1	9,2240E-02	-1,08
Q8R0F6	ILKAP	2,7207E-02	-1,08
Q91YS8	KCC1A	3,2905E-02	-1,07
Q91WN1	DNJC9	4,2421E-02	-1,07
Q6ZWQ0	SYNE2	5,9082E-02	-1,07
Q8K4Z3	NNRE	4,9690E-02	-1,06
P97298	PEDF	3,4020E-02	-1,06
Q9JHQ5	LZTL1	3,5502E-02	-1,06
O35654	DPOD2	4,4564E-02	-1,06
Q9CR86	CHSP1	4,4564E-02	-1,06
Q60590	A1AG1	4,5136E-02	-1,06
Q5SUQ9	CTC1	4,9629E-02	-1,06
Q05D44	IF2P	5,2985E-02	-1,06
Q6EDY6	CARL1	6,7757E-02	-1,06
Q60875	ARHG2	9,3868E-02	-1,06
Q9D906	ATG7	2,8380E-02	-1,05
Q01279	EGFR	2,1693E-02	-1,04
Q9JHK4	PGTA	5,5153E-02	-1,04
Q99P88	NU155	1,1559E-02	-1,04
P45448	NR5A2	9,2119E-02	-1,04
O55201	SUPT5	3,7863E-02	-1,04
Q80VJ3	DNPH1	4,6541E-02	-1,03
Q91W39	NCOA5	4,6607E-02	-1,03
P30681	HMGB2	5,6175E-02	-1,03
P21300	ALD1	5,7983E-03	-1,03
Q9R0Q6	ARC1A	2,3095E-02	-1,01
Q8R2M2	TDIF2	2,0760E-02	-1,00
Q8C180	FRS2	2,1700E-02	-1,00
Q91ZR2	SNX18	2,2508E-02	-1,00
Q52KI8	SRRM1	2,2508E-02	-1,00
O88796	RPP30	2,2957E-02	-1,00
Q9R1E0	FOXO1	2,3083E-02	-1,00
Q8K2V1	PP4R1	2,8320E-02	-1,00
P50427	STS	2,8320E-02	-1,00
P58501	PAXB1	2,9094E-02	-1,00
Q8C0J2	A16L1	2,9094E-02	-1,00
Q9D136	OGFD3	2,9094E-02	-1,00
Q9DBY8	NVL	2,9311E-02	-1,00
Q8CAA7	PGM2L	3,2794E-02	-1,00
Q8K363	DDX18	6,0973E-02	-1,00
O54692	ZW10	6,1547E-02	-1,00
Q8BVA4	LMOD1	6,1786E-02	-1,00
Q9EP97	SENP3	6,5058E-02	-1,00

P51949	MAT1	6,6276E-02	-1,00
Q8K021	SCAM1	6,6276E-02	-1,00
P69566	RANB9	6,6833E-02	-1,00
Q9CYH6	RRS1	6,7752E-02	-1,00
Q6PB93	GALT2	8,2016E-02	-1,00
Q8K0C9	GMDS	9,0445E-02	-1,00
Q8BSQ9	PB1	9,1586E-02	-1,00
Q80UK8	INT2	9,1586E-02	-1,00
Q8BND5	QSOX1	9,9556E-02	-1,00

Table S3. Proteins up-regulated in mutant ovaries

UniprotKB Accession Number	Entry Name	Beta-binomial test	Log2 ratio (M vs C)
Q9QXM0	ABHD2	1,7959E-02	1,88
Q9Z0R9	FADS2	9,6150E-04	1,53
Q8QZR3	EST2A	5,0372E-04	1,51
Q9DBV4	MXRA8	4,7735E-03	1,46
P31428	DPEP1	8,4244E-04	1,38
Q8VCC9	SPON1	3,4193E-03	1,32
Q3UHB1	NT5D3	2,8589E-03	1,32
O35660	GSTM6	6,1031E-03	1,22
Q5NCE8	MRS2	9,9143E-03	1,22
Q9WV68	DECR2	1,0692E-02	1,22
Q60716	P4HA2	3,2168E-03	1,17
Q80ZM8	CRLS1	4,6877E-03	1,17
Q9Z2G9	HTAI2	2,1978E-02	1,17
Q80XN0	BDH	2,6625E-02	1,17
Q8VCT4	CES1D	3,2168E-02	1,16
Q5IRJ6	ZNT9	8,8627E-03	1,15
Q9R049	AMFR	9,2036E-03	1,13
Q9D9V3	ECHD1	1,6262E-02	1,08
Q9QYI4	DJB12	2,1404E-02	1,06
Q9D6K8	FUND2	2,2503E-02	1,06
Q3TYS2	CYBC1	2,7622E-02	1,06
Q9D6K5	SYJ2B	1,4354E-02	1,06
Q920L1	FADS1	1,5442E-02	1,06
Q80U63	MFN2	2,0631E-02	1,06
O70456	1433S	1,9567E-02	1,04
Q9JKK1	STX6	2,1139E-02	1,02
Q8BHC4	DCAKD	3,2486E-03	1,01
P53798	FDFT	4,7805E-03	1,01
O35166	GOSR2	8,6736E-03	1,00
Q80XL6	ACD11	1,7536E-02	1,00
P70265	F262	3,6265E-02	1,00

Table S4. Non-differentially regulated proteins

EXOSC# are noted as EXOS# and DIS3 is noted RRP44

UniprotKB Accession Number	Entry Name	Beta-binomial test	Log2 ratio (M vs C)
Q99PU5	ACBG1	2,2806E-04	0,36
Q8BWT1	THIM	3,4969E-04	0,32
P31324	KAP3	3,6290E-04	0,38
Q8CI94	PYGB	1,1389E-03	-0,84
P47738	ALDH2	1,2255E-03	0,28
Q9QUR6	PPCE	1,9078E-03	-0,72
Q8BLN5	ERG7	2,0126E-03	0,73
Q61553	FSCN1	2,2471E-03	-0,80
P20152	VIME	2,9700E-03	0,10
P98192	GNPAT	3,1886E-03	0,99
P20918	PLMN	4,9860E-03	-0,98
P23953	EST1C	5,0053E-03	-0,93
P15626	GSTM2	5,2231E-03	0,23
P46656	ADX	5,3507E-03	0,41
P15105	GLNA	5,4698E-03	0,51
Q9R1J0	NSDHL	5,6231E-03	0,70
P28665	MUG1	5,7490E-03	-0,94
O88736	DHB7	6,3657E-03	0,88
Q9EQ20	MMSA	6,5978E-03	0,33
P00920	CAH2	7,3926E-03	0,69
Q71RI9	KAT3	7,3958E-03	0,69
P21614	VTDB	7,5377E-03	-0,97
Q9D1Q6	ERP44	7,9039E-03	0,45
P13439	UMPS	8,2535E-03	-0,92
Q3U1J4	DDB1	8,2601E-03	-0,67
Q9D517	PLCC	8,7226E-03	0,88
Q05920	PYC	9,3771E-03	0,22
P52825	CPT2	9,5622E-03	0,33
Q64435	UD16	9,5702E-03	0,93
P53994	RAB2A	9,7568E-03	0,48
P24815	3BHS1	9,9078E-03	0,16
Q9DB73	NB5R1	1,0360E-02	0,74
Q8JZK9	HMCS1	1,0792E-02	0,56
Q64737	PUR2	1,1047E-02	-0,81
O70400	PDLI1	1,1153E-02	-0,91
Q8R4N0	CLYBL	1,2003E-02	0,72
Q00897	A1AT4	1,2029E-02	-0,80

Q62087	PON3	1,2525E-02	0,70
P21981	TGM2	1,3083E-02	0,20
Q8VCW8	ACSF2	1,3556E-02	0,30
Q80VQ0	AL3B1	1,3587E-02	0,83
Q99L04	DHRS1	1,3958E-02	0,59
Q8VEE4	RFA1	1,4614E-02	-0,89
P46735	MYO1B	1,5234E-02	-0,91
Q9EQ06	DHB11	1,5286E-02	0,46
Q9ET54	PALLD	1,6998E-02	-0,82
Q91ZA3	PCCA	1,7243E-02	0,40
Q99J47	DRS7B	1,7575E-02	0,87
Q8BH95	ECHM	1,7587E-02	0,32
Q9R0P9	UCHL1	1,8445E-02	-0,88
P07724	ALBU	1,8936E-02	-0,66
Q8R2Y0	ABHD6	1,9099E-02	0,85
Q8C7R4	UBA6	2,0131E-02	-0,90
Q8BMS1	ECHA	2,0280E-02	0,12
Q9D1A2	CNDP2	2,0381E-02	-0,61
P22599	A1AT2	2,0515E-02	-0,74
A2A432	CUL4B	2,0561E-02	-0,95
O88844	IDHC	2,1276E-02	0,17
Q8R1K1	UBAC2	2,1963E-02	0,81
Q8K0C4	CP51A	2,2286E-02	0,59
Q61147	CERU	2,2810E-02	-0,76
P32507	NECT2	2,2883E-02	0,72
P61922	GABT	2,3126E-02	0,38
Q9QZ82	CP11A	2,3289E-02	0,18
P07758	A1AT1	2,3324E-02	-0,73
Q91VE0	S27A4	2,3377E-02	0,98
Q9EPL9	ACOX3	2,3552E-02	0,51
Q921H8	THIKA	2,4357E-02	0,19
Q9DCM2	GSTK1	2,4368E-02	0,45
Q99JR1	SFXN1	2,4645E-02	0,45
Q99MR8	MCCA	2,5226E-02	0,31
Q8BHD7	PTBP3	2,5347E-02	-0,84
P97742	CPT1A	2,5400E-02	0,29
Q60902	EP15R	2,5717E-02	-0,82
Q8VEM8	MPCP	2,6878E-02	0,32
Q3UMR5	MCU	2,7114E-02	0,84
Q91W36	UBP3	2,7688E-02	0,74
Q61823	PDCD4	2,8405E-02	-0,91
Q01853	TERA	2,9011E-02	0,03
Q64521	GPDM	2,9565E-02	0,39

Q91W86	VPS11	2,9649E-02	0,81
Q91VS7	MGST1	2,9944E-02	0,58
Q8VBZ3	CLPT1	3,0926E-02	0,66
Q8BMK4	CKAP4	3,1812E-02	0,20
Q91XE8	TM205	3,2202E-02	0,81
B1AUE5	PEX10	3,2543E-02	0,81
O08528	HXK2	3,3054E-02	0,32
Q6ZQM8	UD17C	3,3056E-02	0,61
O88428	PAPS2	3,3522E-02	0,60
Q9DBU0	TM9S1	3,4098E-02	0,86
Q8BRT1	CLAP2	3,4686E-02	-0,81
Q8CHT0	AL4A1	3,5256E-02	0,31
Q9D0M3	CY1	3,5409E-02	0,39
P08032	SPTA1	3,5589E-02	0,81
Q8C0F9	PRS35	3,6541E-02	0,91
Q8R1Z9	RN121	3,6809E-02	0,81
P22892	AP1G1	3,7187E-02	-0,89
Q9Z239	PLM	3,7607E-02	0,91
Q00724	RET4	3,7756E-02	-0,85
Q9ERI5	JMJD6	3,7756E-02	-0,85
P62313	LSM6	3,7756E-02	-0,85
Q91V92	ACLY	3,8197E-02	0,10
P61514	RL37A	3,8801E-02	0,82
Q8VCY6	UTP6	3,9108E-02	-0,85
P48377	RFX1	3,9108E-02	-0,85
Q99PM3	TF2AA	3,9108E-02	-0,85
Q8BP27	SFR1	3,9108E-02	-0,85
Q9ESW8	PGPI	3,9108E-02	-0,85
Q9D3U0	PUS10	3,9108E-02	-0,85
A6H8H2	DEN4C	3,9108E-02	-0,85
Q99MI1	RB6I2	3,9108E-02	-0,85
A2AJ15	MA1B1	3,9108E-02	-0,85
Q9JJA2	COG8	3,9108E-02	-0,85
P53762	ARNT	3,9108E-02	-0,85
Q9CQA5	MED4	3,9108E-02	-0,85
O09130	NF2IP	3,9108E-02	-0,85
Q8VEL2	MTMRE	3,9108E-02	-0,85
Q3V1V3	ESF1	3,9108E-02	-0,85
Q80W47	WIPI2	3,9108E-02	-0,85
Q9D0A3	ARPIN	3,9108E-02	-0,85
Q9CWX9	DDX47	3,9108E-02	-0,85
Q8CFQ3	AQR	3,9108E-02	-0,85
P97857	ATS1	3,9115E-02	-0,85

E9Q5K9	YTDC1	3,9258E-02	-0,85
Q9CS42	PRPS2	3,9366E-02	-0,88
P04945	KV6AB	3,9588E-02	-0,85
P10649	GSTM1	3,9970E-02	0,08
Q8R0Y6	AL1L1	4,0425E-02	0,37
P48193	41	4,2375E-02	0,91
Q6PIC6	AT1A3	4,2386E-02	0,87
P36552	HEM6	4,4773E-02	0,41
P63038	CH60	4,4922E-02	0,27
Q9JLZ3	AUHM	4,5248E-02	0,74
Q3U1V6	UEVLD	4,5892E-02	0,97
Q9CXD6	MCUR1	4,6750E-02	0,91
A6X935	ITIH4	4,6850E-02	-0,79
P17225	PTBP1	4,7040E-02	-0,54
P97434	MPRIP	4,7761E-02	-0,94
P51174	ACADL	4,8039E-02	0,12
Q61578	ADRO	4,8472E-02	0,11
Q9JJK2	LANC2	4,8875E-02	-0,95
P38647	GRP75	4,9261E-02	0,05
Q9CWS0	DDAH1	4,9451E-02	-0,84
Q8CC88	VWA8	4,9614E-02	0,69
Q91WK1	SPRY4	4,9715E-02	0,91
Q8BKG3	PTK7	4,9743E-02	-0,66
P17710	HXK1	4,9856E-02	0,22
Q6P542	ABCF1	4,9953E-02	0,41
Q8BSE0	RMD2	5,0283E-02	0,30
Q9D6M3	GHC1	5,0513E-02	0,68
P27048	RSMB	5,0598E-02	-0,89
P19096	FAS	5,0702E-02	0,13
Q921G7	ETFD	5,1214E-02	0,24
O08547	SC22B	5,1255E-02	0,42
P63268	ACTH	5,1647E-02	-0,42
Q61703	ITIH2	5,1881E-02	-0,95
Q8C052	MAP1S	5,2731E-02	-0,95
Q91YE6	IPO9	5,2768E-02	-0,66
Q9CQN1	TRAP1	5,3681E-02	0,13
O54734	OST48	5,4244E-02	0,28
Q3TDD9	PPR21	5,5004E-02	-0,93
O35459	ECH1	5,5366E-02	0,29
P01027	CO3	5,5956E-02	-0,52
Q08122	TLE3	5,6351E-02	0,91
P06801	MAOX	5,7650E-02	0,03
P01867	IGG2B	5,7675E-02	-0,76

Q9CZX0	ELP3	5,7700E-02	-0,95
Q9CZX9	EMC4	5,8026E-02	0,84
P01942	HBA	5,8817E-02	0,21
Q3TC72	FAHD2	6,0140E-02	0,51
Q5DTU0	AF1L2	6,0365E-02	0,84
Q9DBL1	ACDSB	6,0402E-02	0,25
P45700	MA1A1	6,1402E-02	0,58
Q78HU7	GLPC	6,1402E-02	0,58
Q9D975	SRXN1	6,1402E-02	0,58
Q9Z0J1	RECK	6,1402E-02	0,58
P98197	AT11A	6,1402E-02	0,58
Q9D0I4	STX17	6,1402E-02	0,58
Q9D8B6	F210B	6,1402E-02	0,58
O70281	TPST1	6,1402E-02	0,58
Q9CPS6	HINT3	6,1402E-02	0,58
Q8R5K2	UBP33	6,1402E-02	0,58
P39655	LOX12	6,1402E-02	0,58
Q60760	GRB10	6,1402E-02	0,58
Q61712	DNJC1	6,1402E-02	0,58
Q91XC9	PEX16	6,1402E-02	0,58
Q6P6J9	TXD15	6,1402E-02	0,58
Q8R4K2	IRAK4	6,1402E-02	0,58
O70480	VAMP4	6,1402E-02	0,58
P25119	TNR1B	6,1402E-02	0,58
P39098	MA1A2	6,1402E-02	0,58
Q8VDY9	CAAP1	6,1402E-02	0,58
Q8BSK8	KS6B1	6,1402E-02	0,58
Q3UR78	CD048	6,1402E-02	0,58
Q4VC33	MAEA	6,1402E-02	0,58
Q8BWY9	CIP2A	6,1402E-02	0,58
P08113	ENPL	6,1703E-02	0,02
P24472	GSTA4	6,1925E-02	0,44
Q8R5M0	GIPC3	6,2897E-02	0,58
Q9R0M6	RAB9A	6,3000E-02	0,74
Q8R010	AIMP2	6,3044E-02	-0,83
Q9DCD0	6PGD	6,3093E-02	0,18
Q61735	CD47	6,3693E-02	0,74
Q91Y97	ALDOB	6,4310E-02	0,52
Q8BZM1	GLMN	6,4569E-02	-0,85
Q6P1H6	ANKL2	6,4569E-02	-0,85
Q03265	ATPA	6,4612E-02	0,02
P56480	ATPB	6,4693E-02	-0,01
Q9QZD8	DIC	6,4766E-02	0,54

Q99KV1	DJB11	6,4772E-02	0,38
Q9CQU3	RER1	6,5267E-02	0,54
P06728	APOA4	6,5364E-02	-0,88
Q9WUA3	PFKAP	6,5443E-02	-0,69
P55096	ABCD3	6,5640E-02	0,42
Q8VDG3	PARN	6,5750E-02	-0,97
P51660	DHB4	6,6037E-02	0,15
P82343	RENBP	6,6258E-02	-0,97
Q6P2B1	TNPO3	6,6300E-02	-0,86
Q99MN9	PCCB	6,6745E-02	0,17
P80316	TCPE	6,7702E-02	-0,45
Q3TCN2	PLBL2	6,7959E-02	-0,94
O70433	FHL2	6,7969E-02	-0,86
Q91XI1	DUS3L	6,8142E-02	-0,87
P23116	EIF3A	6,8158E-02	-0,78
P06795	MDR1B	6,8164E-02	0,75
Q9CSH3	RRP44	6,8366E-02	-0,98
Q9Z2Q6	SEPT5	6,8447E-02	0,79
Q8R3Q0	SARAF	6,9142E-02	0,66
Q8BFW7	LPP	6,9311E-02	-0,57
Q9JI78	NGLY1	6,9449E-02	0,66
Q9JHI5	IVD	6,9657E-02	0,26
P97314	CSRP2	6,9773E-02	-0,95
Q8CGK3	LONM	6,9865E-02	0,09
P84091	AP2M1	7,0108E-02	0,39
Q8QZS1	HIBCH	7,0421E-02	0,30
Q8BUY5	TIDC1	7,0701E-02	0,85
P55302	AMRP	7,0959E-02	0,58
Q9R0H0	ACOX1	7,1785E-02	0,30
O55186	CD59A	7,1993E-02	-0,68
P58802	TB10A	7,1993E-02	-0,68
Q91YR5	EFNMT	7,1993E-02	-0,68
Q8VD04	GRAP1	7,1993E-02	-0,68
O88878	ZFAN5	7,1993E-02	-0,68
Q9JHR9	NRIP2	7,1993E-02	-0,68
Q6PE15	ABHDA	7,1993E-02	-0,68
Q921E6	EED	7,1993E-02	-0,68
Q80X71	T106B	7,1993E-02	-0,68
Q8BVW3	TRI14	7,1993E-02	-0,68
Q9DB85	RRP8	7,1993E-02	-0,68
P03976	KV2A5	7,1993E-02	-0,68
A2A5R2	BIG2	7,1993E-02	-0,68
Q64302	T4S1	7,1993E-02	-0,68

Q9CQA3	SDHB	7,2140E-02	0,30
Q9JMB0	GKAP1	7,2271E-02	-0,85
P97355	SPSY	7,2506E-02	-0,94
Q6P5F9	XPO1	7,2576E-02	-0,56
O70589	CSKP	7,2673E-02	-0,92
P24547	IMDH2	7,3129E-02	-0,56
Q9D024	CCD47	7,3564E-02	0,35
Q8K3G5	VRK3	7,3607E-02	-0,94
Q8CI33	C19L1	7,3607E-02	-0,94
Q9Z2I8	SUCB2	7,4183E-02	0,15
Q5DTM8	BRE1A	7,4340E-02	-0,93
Q7TNE3	SPAG7	7,4666E-02	-0,94
Q9Z1K6	ARI2	7,4666E-02	-0,94
O35250	EXOC7	7,4666E-02	-0,94
Q9DCX2	ATP5H	7,5259E-02	0,28
Q9D7B6	ACAD8	7,5485E-02	0,22
Q9CRC8	LRC40	7,5963E-02	-0,79
Q61129	CFAI	7,6522E-02	-0,94
Q9DCW4	ETFB	7,6670E-02	0,16
P29351	PTN6	7,6985E-02	-0,85
Q8BGZ4	CDC23	7,7243E-02	-0,74
P58281	OPA1	7,7401E-02	0,21
B2RQC6	PYR1	7,7656E-02	-0,62
Q9JKR6	HYOU1	7,8304E-02	0,10
Q8VED9	LEGL	7,8841E-02	-0,96
P54818	GALC	7,9333E-02	-0,96
Q9Z1P7	KANK3	7,9592E-02	0,74
Q9DBM2	ECHP	7,9592E-02	0,74
Q3UGR5	HDHD2	8,0017E-02	0,64
Q9JM76	ARPC3	8,0054E-02	-0,80
P52196	THTR	8,0093E-02	0,21
Q62048	PEA15	8,0362E-02	-0,96
Q61490	CD166	8,0638E-02	-0,68
Q80WB5	NTAQ1	8,0638E-02	-0,68
P59481	LMA2L	8,0638E-02	-0,68
P60605	UB2G2	8,0638E-02	-0,68
Q8CG47	SMC4	8,0638E-02	-0,68
Q8C0P5	COR2A	8,0638E-02	-0,68
Q8BL74	TF3C2	8,0638E-02	-0,68
P33434	MMP2	8,0638E-02	-0,68
A3KGB4	TBC8B	8,0638E-02	-0,68
Q9CPT3	NANP	8,0638E-02	-0,68
P01723	LV1A	8,0638E-02	-0,68

Q8BJ03	COX15	8,0638E-02	-0,68
Q7TMQ7	WDR91	8,0638E-02	-0,68
P60122	RUVB1	8,0841E-02	-0,48
Q8R5K4	NOL6	8,1708E-02	-0,94
Q60710	SAMH1	8,2146E-02	-0,58
Q62230	SN	8,2352E-02	0,74
O88874	CCNK	8,2539E-02	-0,68
Q3TVI8	PBIP1	8,2539E-02	-0,68
Q91YD9	WASL	8,2539E-02	-0,68
Q8BN58	RHG28	8,2702E-02	-0,68
P70268	PKN1	8,2702E-02	-0,68
P52431	DPOD1	8,2702E-02	-0,68
Q8C9B9	DIDO1	8,2702E-02	-0,68
Q14CH7	SYAM	8,2702E-02	-0,68
Q7TSZ8	NACC1	8,2702E-02	-0,68
B2RXC1	TPC11	8,2702E-02	-0,68
Q8VDD8	WASH1	8,2702E-02	-0,68
P51658	DHB2	8,4441E-02	-0,93
Q8BMC4	NOP9	8,4461E-02	-0,68
P25425	PO2F1	8,4461E-02	-0,68
Q9EPL8	IPO7	8,4469E-02	-0,58
Q6PAC3	DCA13	8,4977E-02	-0,68
Q8VCG3	WDR74	8,4977E-02	-0,68
Q8C0Z1	F234A	8,4977E-02	-0,68
Q05816	FABP5	8,5341E-02	-0,62
Q9Z1Q5	CLIC1	8,5348E-02	0,23
Q3U319	BRE1B	8,5783E-02	-0,89
Q5SW19	CLU	8,5867E-02	-0,98
Q8BI84	TGO1	8,6207E-02	0,56
Q80V03	ADCK5	8,6234E-02	0,47
Q8VEE1	LMCD1	8,6435E-02	-0,66
P70388	RAD50	8,6670E-02	-0,85
Q9Z160	COG1	8,6670E-02	-0,85
Q8BUE4	AIFM2	8,6732E-02	0,47
Q8BGS2	BOLA2	8,6858E-02	-0,94
Q8K297	GT251	8,6881E-02	-0,71
Q9D379	HYEP	8,6934E-02	0,13
Q9D0B6	PBDC1	8,6949E-02	-0,82
P63280	UBC9	8,6995E-02	-0,82
Q8BIA4	FBXW8	8,7228E-02	-0,93
Q64127	TIF1A	8,7577E-02	-0,94
Q8VHL1	SETD7	8,7911E-02	-0,96
Q8BTI8	SRRM2	8,8016E-02	-0,80

Q9CRB9	MIC19	8,8021E-02	0,35
Q8C878	UBA3	8,8046E-02	-0,64
Q689Z5	SBNO1	8,8463E-02	-0,85
Q570Y9	DPTOR	8,8463E-02	-0,85
O70494	SP3	8,8604E-02	-0,85
Q5RJG1	NOL10	8,8604E-02	-0,85
Q9CWE6	OOEP	8,8604E-02	-0,85
Q8VDD9	PHIP	8,8604E-02	-0,85
O88455	DHCR7	8,8809E-02	0,35
Q61699	HS105	8,9206E-02	-0,48
P11103	PARP1	8,9989E-02	-0,62
P17918	PCNA	9,0175E-02	-0,56
Q8BLF1	NCEH1	9,0709E-02	-0,68
Q9D0F6	RFC5	9,0968E-02	-0,90
A2ADY9	DDI2	9,1145E-02	-0,71
Q60929	MEF2A	9,1171E-02	-0,68
P58854	GCP3	9,1171E-02	-0,68
Q6DID3	SCAF8	9,1171E-02	-0,68
O35598	ADA10	9,1171E-02	-0,93
Q60597	ODO1	9,1320E-02	0,04
P62737	ACTA	9,1326E-02	-0,42
Q9Z1W8	AT12A	9,1449E-02	0,63
O54724	CAVN1	9,1658E-02	0,17
Q8BYH7	TBC17	9,1693E-02	-0,86
Q3ULJ0	GPD1L	9,1790E-02	0,50
P11881	ITPR1	9,1901E-02	-0,86
Q9CXY9	GPI8	9,2355E-02	0,49
Q9D787	PPIL2	9,2586E-02	-0,88
Q9WVH9	FBLN5	9,3817E-02	-0,88
Q61704	ITIH3	9,3927E-02	-0,68
Q61425	HCDH	9,3992E-02	0,27
P54822	PUR8	9,4118E-02	-0,64
Q99K48	NONO	9,4425E-02	-0,46
Q9Z2B9	KS6A4	9,5716E-02	-0,68
Q3UHH8	GXLT1	9,5716E-02	-0,68
O08992	SDCB1	9,5716E-02	-0,68
O08677	KNG1	9,5812E-02	-0,62
Q61879	MYH10	9,5895E-02	-0,50
Q99JW2	ACY1	9,5980E-02	-0,90
Q9CV28	MINY3	9,6017E-02	-0,68
Q61838	PZP	9,6305E-02	-0,49
Q6ZPY7	KDM3B	9,6949E-02	-0,85
Q6A058	ARMX2	9,7296E-02	0,49

Q6ZQK5	ACAP2	9,7443E-02	0,51
P30730	LSHR	9,7472E-02	0,96
P10852	4F2	9,7554E-02	0,15
Q924K8	MTA3	9,7634E-02	-0,80
P51569	AGAL	9,7805E-02	-0,90
P57746	VATD	9,8463E-02	0,67
O08739	AMPD3	9,8496E-02	0,52
Q8C5H8	NAKD2	9,8597E-02	0,36
O70172	PI42A	9,8656E-02	0,51
Q99K01	PDXD1	9,9248E-02	0,24
Q9CY21	BUD23	9,9263E-02	-0,90
Q62219	TGFI1	9,9776E-02	-0,76
Q9QY76	VAPB	9,9820E-02	0,32
P50431	GLYC	1,0012E-01	-0,74
P02798	MT2	1,0019E-01	-0,90
Q07456	AMBP	1,0019E-01	-0,90
Q6PDN3	MYLK	1,0019E-01	-0,53
Q9DBB9	CPN2	1,0033E-01	-0,68
Q8R0W0	EPIPL	1,0071E-01	-0,68
Q00493	CBPE	1,0083E-01	-0,76
P15508	SPTB1	1,0094E-01	1,05
P97447	FHL1	1,0096E-01	-0,60
Q9QZ06	TOLIP	1,0124E-01	0,64
P62315	SMD1	1,0139E-01	-0,76
P0C8K7	SMIM1	1,0192E-01	0,64
P60898	RPB9	1,0213E-01	-0,90
Q3ULD5	MCCB	1,0237E-01	0,19
Q61183	PAPOA	1,0273E-01	-0,87
Q7TNG8	LDHD	1,0340E-01	0,76
Q8BNV1	TRM2A	1,0392E-01	-0,94
Q3UN02	LCLT1	1,0402E-01	0,62
Q80U93	NU214	1,0422E-01	-1,12
Q9Z0X1	AIFM1	1,0430E-01	0,12
O35239	PTN9	1,0460E-01	0,58
Q9CZU3	MTREX	1,0486E-01	-0,65
Q68FL6	SYMC	1,0498E-01	-0,53
Q8BX02	KANK2	1,0505E-01	-0,58
Q64133	AOFA	1,0511E-01	0,20
Q3UW53	NIBA1	1,0573E-01	0,27
Q8BWU5	OSGEP	1,0584E-01	-0,77
P04919	B3AT	1,0623E-01	0,71
Q6PE01	SNR40	1,0661E-01	-0,77
Q8VDW0	DX39A	1,0679E-01	-0,68

Q9D328	TM35A	1,0805E-01	0,64
Q8K224	NAT10	1,0871E-01	-0,84
Q91X72	HEMO	1,0890E-01	-0,62
Q9ESZ8	GTF2I	1,0894E-01	-0,54
Q62009	POSTN	1,0902E-01	-0,88
Q8R127	SCPDL	1,0936E-01	0,62
P16546	SPTN1	1,0960E-01	0,01
Q9Z127	LAT1	1,0975E-01	0,42
Q8BW75	AOFB	1,0982E-01	0,42
Q04736	YES	1,1017E-01	0,43
Q8K0D5	EFGM	1,1039E-01	0,32
O89020	AFAM	1,1064E-01	-0,71
O88856	TPST2	1,1071E-01	0,68
Q06770	CBG	1,1206E-01	-0,78
Q6PDI5	ECM29	1,1246E-01	-0,68
Q921F4	HNRLL	1,1252E-01	-0,55
Q920B9	SP16H	1,1280E-01	-0,58
Q5SWU9	ACACA	1,1324E-01	1,03
O88811	STAM2	1,1361E-01	-0,88
Q8VHX6	FLNC	1,1433E-01	-0,49
Q8BHS3	RBM22	1,1472E-01	-0,78
Q9R112	SQOR	1,1548E-01	0,36
Q8K2B3	SDHA	1,1582E-01	0,05
P00375	DYR	1,1585E-01	-0,87
P21447	MDR1A	1,1586E-01	0,45
P51655	GPC4	1,1689E-01	-0,61
Q8C2E7	WASC5	1,1698E-01	-1,26
P07759	SPA3K	1,1701E-01	-0,48
P68181	KAPCB	1,1716E-01	-0,86
Q8BFR4	GNS	1,1771E-01	-0,85
Q8CAQ8	MIC60	1,1778E-01	0,07
P01631	KV2A7	1,1810E-01	-0,78
Q9CYZ8	SSBP2	1,1914E-01	-0,82
Q8CGU1	CACO1	1,1914E-01	-0,82
Q99PG2	OGFR	1,1914E-01	-0,82
A2AQ19	RTF1	1,1914E-01	-0,82
Q00899	TYY1	1,2041E-01	-0,96
Q9D0D5	T2EA	1,2050E-01	-0,85
Q62018	CTR9	1,2091E-01	-0,87
P01837	IGKC	1,2093E-01	-0,65
Q9QZH3	PPIE	1,2107E-01	-0,82
Q4VA53	PDS5B	1,2174E-01	-0,93
E9Q557	DESP	1,2197E-01	-3,06

P23475	XRCC6	1,2266E-01	-0,80
Q8BKC5	IPO5	1,2286E-01	-0,42
Q8CI95	OSB11	1,2296E-01	-0,85
Q62318	TIF1B	1,2379E-01	-0,42
Q99LX0	PARK7	1,2392E-01	0,13
P39447	ZO1	1,2464E-01	-0,59
Q91W50	CSDE1	1,2483E-01	0,22
P35293	RAB18	1,2502E-01	0,23
Q9DBZ5	EIF3K	1,2533E-01	-0,82
Q9R118	HTRA1	1,2544E-01	-0,83
P48678	LMNA	1,2552E-01	0,00
Q9D6Z1	NOP56	1,2577E-01	-0,51
Q9Z103	ADNP	1,2630E-01	-1,06
Q9QYJ3	DNJB1	1,2702E-01	-0,77
Q7TPV4	MBB1A	1,2757E-01	-1,02
O08638	MYH11	1,2763E-01	-0,43
P35235	PTN11	1,2793E-01	-0,57
Q9CZ44	NSF1C	1,2818E-01	-0,52
P63011	RAB3A	1,2845E-01	0,45
P34884	MIF	1,2859E-01	-0,72
Q8R016	BLMH	1,2885E-01	-0,63
O88967	YMEL1	1,2917E-01	0,36
P62242	RS8	1,2921E-01	0,16
Q8R2Y2	MUC18	1,2923E-01	-0,72
E9Q4P1	WDFY1	1,2958E-01	0,66
O08579	EMD	1,3063E-01	0,27
Q8R1I1	QCR9	1,3111E-01	0,44
Q9CXT8	MPPB	1,3155E-01	0,24
Q925F2	ESAM	1,3184E-01	-0,82
Q60715	P4HA1	1,3236E-01	0,22
P26638	SYSC	1,3257E-01	0,17
E9Q7G0	NUMA1	1,3282E-01	-0,56
Q64523	H2A2C	1,3296E-01	-0,75
Q91YN9	BAG2	1,3298E-01	-0,82
Q9CZU6	CISY	1,3349E-01	0,18
Q5SWT3	S2535	1,3358E-01	0,37
Q7TMY8	HUWE1	1,3405E-01	-1,18
Q99LD8	DDAH2	1,3475E-01	0,23
P08030	APT	1,3495E-01	-0,72
P55264	ADK	1,3520E-01	-0,57
Q6ZWZ2	UB2R2	1,3551E-01	0,64
Q924C1	XPO5	1,3622E-01	-0,73
Q8VCL2	SCO2	1,3663E-01	1,20

Q91VR2	ATPG	1,3714E-01	0,15
Q99JY0	ECHB	1,3714E-01	0,06
Q8VBW6	ULA1	1,3721E-01	-0,68
O35218	CPSF2	1,3793E-01	-0,68
Q02357	ANK1	1,3807E-01	1,20
Q921I1	TRFE	1,3830E-01	-0,44
Q07417	ACADS	1,3844E-01	0,12
Q9EPU4	CPSF1	1,3869E-01	-0,73
Q9ESU6	BRD4	1,3902E-01	-0,49
Q8K2F0	BRD3	1,3902E-01	-0,49
Q61301	CTNA2	1,3921E-01	0,55
P47802	MTX1	1,3975E-01	0,32
Q5HZI9	S2551	1,3978E-01	0,64
Q3UA37	QRIC1	1,4020E-01	-0,73
Q8BIJ6	SYIM	1,4099E-01	0,13
P62869	ELOB	1,4131E-01	-0,73
Q99MN1	SYK	1,4160E-01	0,15
P21844	CMA1	1,4167E-01	0,54
Q3THK7	GUAA	1,4170E-01	-0,48
O88876	DHRS3	1,4183E-01	-0,49
P29037	TBP	1,4183E-01	-0,49
Q8R0A0	T2FB	1,4183E-01	-0,49
Q923E4	SIR1	1,4183E-01	-0,49
Q99MY8	ASH1L	1,4183E-01	-0,49
Q63810	CANB1	1,4183E-01	-0,49
Q8BFU3	RN214	1,4183E-01	-0,49
Q99LN9	DOHH	1,4183E-01	-0,49
Q8BQ30	PPR18	1,4183E-01	-0,49
O55236	MCE1	1,4183E-01	-0,49
Q921I2	KLDC4	1,4183E-01	-0,49
Q8BQZ5	CPSF4	1,4183E-01	-0,49
P33611	DPOA2	1,4183E-01	-0,49
A6H5X4	PHF11	1,4183E-01	-0,49
Q8CG73	FTM	1,4183E-01	-0,49
Q8C3I8	HGH1	1,4183E-01	-0,49
Q3UM18	LSG1	1,4183E-01	-0,49
Q9D0I8	MRT4	1,4183E-01	-0,49
Q6A065	CE170	1,4183E-01	-0,49
Q5XJE5	LEO1	1,4183E-01	-0,49
Q80ZV0	RNH2B	1,4183E-01	-0,49
Q80TN4	DJC16	1,4183E-01	-0,49
Q9CY97	SSU72	1,4183E-01	-0,49
Q923D5	WBP11	1,4183E-01	-0,49

Q8BIK4	DOCK9	1,4183E-01	-0,49
Q9QZF2	GPC1	1,4183E-01	-0,49
P59470	RPC2	1,4183E-01	-0,49
Q8VDC1	FYCO1	1,4183E-01	-0,49
B2RRD7	BRPF1	1,4183E-01	-0,49
Q8BWR2	PITH1	1,4183E-01	-0,49
Q8BJ64	CHDH	1,4183E-01	-0,49
Q920Q4	VPS16	1,4183E-01	-0,49
Q80W85	NPM2	1,4183E-01	-0,49
Q8K0H5	TAF10	1,4183E-01	-0,49
Q9CQ18	RNH2C	1,4183E-01	-0,49
O70258	SGCE	1,4183E-01	-0,49
Q9QXG2	RAE1	1,4183E-01	-0,49
Q60994	ADIPO	1,4183E-01	-0,49
Q9EQH4	TAF8	1,4183E-01	-0,49
Q8C5P5	NT5D1	1,4183E-01	-0,49
Q8JZL3	THTPA	1,4183E-01	-0,49
P57080	UBP25	1,4183E-01	-0,49
P63139	NFYB	1,4183E-01	-0,49
P11672	NGAL	1,4183E-01	-0,49
P01654	KV3A1	1,4183E-01	-0,49
Q91WE2	PIP30	1,4183E-01	-0,49
Q6NSQ7	LTV1	1,4183E-01	-0,49
Q8VE11	MTMR6	1,4183E-01	-0,49
Q8VEG4	EXD2	1,4183E-01	-0,49
Q8CD92	TTC27	1,4183E-01	-0,49
Q9CY73	RM44	1,4183E-01	-0,49
Q8K1N2	PHLB2	1,4183E-01	-0,49
Q9Z1B5	MD2L1	1,4183E-01	-0,49
Q0GNC1	INF2	1,4183E-01	-0,49
Q8K4M5	COMD1	1,4183E-01	-0,49
Q8K330	SSH3	1,4183E-01	-0,49
E9Q4N7	ARI1B	1,4183E-01	-0,49
Q9ESE1	LRBA	1,4183E-01	-0,49
Q91YL2	RN126	1,4183E-01	-0,49
P63082	VATL	1,4183E-01	-0,49
Q505F1	NR2C1	1,4183E-01	-0,49
Q8C4B4	U119B	1,4183E-01	-0,49
P01725	LV1C	1,4183E-01	-0,49
Q8BX94	OSBL2	1,4183E-01	-0,49
P19785	ESR1	1,4183E-01	-0,49
Q8BVU0	LRCH3	1,4183E-01	-0,49
Q9JLI6	SCLY	1,4183E-01	-0,49

Q9D289	TPC6B	1,4183E-01	-0,49
Q9R098	HGFA	1,4183E-01	-0,49
P21271	MYO5B	1,4183E-01	-0,49
P70700	RPA2	1,4183E-01	-0,49
Q91YY4	ATPF2	1,4183E-01	-0,49
Q810J8	ZFYV1	1,4183E-01	-0,49
P12023	A4	1,4183E-01	-0,49
Q3U821	WDR75	1,4183E-01	-0,49
Q9QZR0	RNF25	1,4183E-01	-0,49
Q8K1H7	T11L2	1,4183E-01	-0,49
Q9WUB4	DCTN6	1,4183E-01	-0,49
Q8VCB2	MED25	1,4183E-01	-0,49
Q3U487	HECD3	1,4183E-01	-0,49
Q8BTM8	FLNA	1,4226E-01	-0,28
Q8BK48	EST2E	1,4228E-01	0,64
Q9Z1Y4	TRIP6	1,4268E-01	-0,90
P24549	AL1A1	1,4290E-01	-0,03
Q8BGC4	PTGR3	1,4361E-01	0,20
Q9CYI0	NJMU	1,4509E-01	-0,79
Q3KNM2	MARH5	1,4511E-01	0,47
Q61508	ECM1	1,4575E-01	0,37
Q80W21	GSTM7	1,4678E-01	0,33
Q921I0	ORML1	1,4722E-01	0,61
A2ALW5	DJC25	1,4722E-01	0,61
Q8R2Q8	BST2	1,4729E-01	-0,90
Q8CCH2	NHLC3	1,4798E-01	-0,79
Q9DBG5	PLIN3	1,4914E-01	-0,50
Q9Z2Z9	GFPT2	1,4946E-01	-0,72
P19973	LSP1	1,5011E-01	0,47
Q99LC5	ETFA	1,5017E-01	0,07
P50544	ACADV	1,5025E-01	0,11
Q9CR20	IR3IP	1,5030E-01	0,61
Q9NYQ2	HAOX2	1,5035E-01	0,47
Q9D1D4	TMEDA	1,5066E-01	0,17
Q78ZA7	NP1L4	1,5120E-01	-0,51
P50171	DHB8	1,5125E-01	0,26
Q8R0N6	HOT	1,5140E-01	0,49
Q8CGZ0	CHERP	1,5171E-01	-0,73
Q9Z1J3	NFS1	1,5177E-01	0,26
P03953	CFAD	1,5190E-01	-0,68
Q91YP3	DEOC	1,5283E-01	-0,79
Q9DBG7	SRPRA	1,5303E-01	0,58
Q99PL5	RRBP1	1,5310E-01	0,09

Q9ERU9	RBP2	1,5330E-01	-0,60
Q9DC11	PXDC2	1,5342E-01	-0,73
Q6IRU2	TPM4	1,5344E-01	0,17
P49222	EPB42	1,5390E-01	0,58
Q9R078	AAKB1	1,5390E-01	0,58
P59242	CING	1,5390E-01	0,58
P06802	ENPP1	1,5393E-01	-1,06
P50429	ARSB	1,5411E-01	0,37
Q9CQA1	TPPC5	1,5522E-01	0,58
Q9WTR5	CAD13	1,5522E-01	0,58
O09000	NCOA3	1,5522E-01	0,58
Q8R404	MIC13	1,5522E-01	0,58
Q02819	NUCB1	1,5534E-01	-0,50
Q9CXA2	T3HPD	1,5580E-01	-0,68
Q5SVQ0	KAT7	1,5587E-01	-0,79
Q9CQ92	FIS1	1,5591E-01	0,26
O55143	AT2A2	1,5618E-01	0,05
Q69Z99	ZN512	1,5761E-01	0,50
Q9QYF1	RDH11	1,5761E-01	0,50
Q99LB2	DHRS4	1,5782E-01	0,37
Q8R2Z5	VWA1	1,5801E-01	0,50
Q8BHF7	PGPS1	1,5848E-01	0,50
Q61335	BAP31	1,5916E-01	0,50
Q9WTS2	FUT8	1,5922E-01	0,56
Q9CXR1	DHRS7	1,5926E-01	0,26
Q60953	PML	1,5930E-01	-0,68
Q8CD91	SMOC2	1,5933E-01	0,81
Q3TBT3	STING	1,5985E-01	0,58
Q8R3C0	MCMBP	1,5986E-01	-0,74
Q9CQ62	DECR	1,6002E-01	0,19
Q9CWI3	BCCIP	1,6027E-01	-0,75
Q61738	ITA7	1,6037E-01	0,58
Q08501	PRLR	1,6037E-01	0,58
Q71KT5	ERG24	1,6037E-01	0,58
Q9R1L5	MAST1	1,6045E-01	0,59
Q8BMP6	GCP60	1,6055E-01	0,32
Q925N2	SFXN2	1,6088E-01	0,51
Q80UM7	MOGS	1,6099E-01	-0,71
P27786	CP17A	1,6118E-01	1,00
Q8K183	PDXK	1,6121E-01	0,29
Q8BWF0	SSDH	1,6122E-01	0,17
P28667	MRP	1,6130E-01	0,53
Q3TEA8	HP1B3	1,6184E-01	-0,68

Q80TM9	NISCH	1,6205E-01	-1,06
Q920A5	RISC	1,6215E-01	0,20
Q9QYE6	GOGA5	1,6259E-01	0,54
P63044	VAMP2	1,6284E-01	0,56
Q5RKZ7	MOCS1	1,6299E-01	0,56
Q9JIG8	PRAF2	1,6313E-01	0,53
Q922H1	ANM3	1,6337E-01	-0,78
P62309	RUXG	1,6345E-01	0,54
Q9QXW9	LAT2	1,6345E-01	0,54
P46460	NSF	1,6397E-01	0,22
Q8CI51	PDLI5	1,6460E-01	-0,60
Q8BP71	RFOX2	1,6466E-01	-0,74
Q80Y81	RNZ2	1,6519E-01	-0,75
P81117	NUCB2	1,6551E-01	0,29
Q8BNU0	ARMC6	1,6603E-01	-0,77
B2RXS4	PLXB2	1,6616E-01	-0,68
Q8C4J7	TBL3	1,6618E-01	-0,78
Q8K2I1	FNTB	1,6644E-01	-0,79
Q8BZG5	RRNAD	1,6682E-01	0,58
Q8BYZ1	ABI3	1,6682E-01	0,58
O35435	PYRD	1,6718E-01	0,38
Q8BVF2	PDCL3	1,6732E-01	-0,78
Q9D554	SF3A3	1,6744E-01	-0,49
Q91VR5	DDX1	1,6770E-01	-0,44
Q6GQT9	NOMO1	1,6893E-01	0,17
Q99LJ0	CT2NL	1,6931E-01	-0,75
Q8CIG8	ANM5	1,6962E-01	-0,76
Q9WUZ9	ENTP5	1,6971E-01	0,38
Q921M7	FA49B	1,7025E-01	-0,59
Q8K1R7	NEK9	1,7045E-01	-1,00
Q99ME9	NOG1	1,7045E-01	-0,76
Q9CR68	UCRI	1,7050E-01	0,22
Q9R0P3	ESTD	1,7073E-01	-0,45
Q91ZW3	SMCA5	1,7112E-01	-0,58
Q6ZWV3	RL10	1,7127E-01	0,18
Q14C51	PTCD3	1,7139E-01	-0,77
Q9DBZ1	IKIP	1,7188E-01	0,36
Q9D2G2	ODO2	1,7193E-01	0,18
Q9QUI0	RHOA	1,7255E-01	0,19
Q91ZH7	ABHD3	1,7303E-01	0,74
P28740	KIF2A	1,7304E-01	-0,76
Q8BG51	MIRO1	1,7414E-01	0,27
Q99KI3	EMC3	1,7449E-01	0,38

P13634	CAH1	1,7496E-01	0,87
P17563	SBP1	1,7510E-01	0,09
Q64012	RALY	1,7511E-01	0,32
Q99JW4	LIMS1	1,7589E-01	-0,53
P62918	RL8	1,7609E-01	0,22
Q8BMB3	IF4E2	1,7625E-01	0,54
P59598	ASXL1	1,7625E-01	0,54
Q3UFS0	ZY11B	1,7625E-01	0,54
O88974	SETB1	1,7625E-01	0,54
Q8BZ20	PAR12	1,7625E-01	0,54
Q8VHK9	DHX36	1,7667E-01	-0,87
P52293	IMA1	1,7699E-01	-0,73
Q9Z1G4	VPP1	1,7717E-01	0,38
Q9QYG0	NDRG2	1,7719E-01	0,22
Q61263	SOAT1	1,7846E-01	0,16
Q91V12	BACH	1,7860E-01	0,22
Q9CYT6	CAP2	1,7979E-01	-0,48
P70168	IMB1	1,8017E-01	-0,41
Q9WUN2	TBK1	1,8018E-01	-0,77
P62983	RS27A	1,8036E-01	0,06
Q99P72	RTN4	1,8097E-01	0,23
Q9WV55	VAPA	1,8136E-01	0,16
P50136	ODBA	1,8172E-01	0,32
Q8CGC7	SYEP	1,8174E-01	-0,36
Q9CQU0	TXD12	1,8209E-01	-0,75
O70318	E41L2	1,8217E-01	-0,55
P58021	TM9S2	1,8246E-01	0,32
Q8BH79	ANO10	1,8258E-01	0,51
Q8VDL4	ADPGK	1,8301E-01	0,18
Q8K4Z5	SF3A1	1,8312E-01	-0,50
Q6PHN9	RAB35	1,8313E-01	0,36
Q8C0I1	ADAS	1,8330E-01	0,17
P47964	RL36	1,8396E-01	0,54
Q3ULF4	SPG7	1,8396E-01	0,54
Q3U2A8	SYVM	1,8396E-01	0,54
Q9JM96	BORG4	1,8396E-01	0,54
Q61733	RT31	1,8396E-01	0,54
Q61112	CAB45	1,8434E-01	0,54
P58058	NADK	1,8455E-01	0,54
Q9D1M0	SEC13	1,8507E-01	0,18
Q9DBJ1	PGAM1	1,8509E-01	0,04
Q99M31	HSP7E	1,8511E-01	-0,68
Q9WVJ3	CBPQ	1,8539E-01	-0,64

P30999	CTND1	1,8553E-01	-0,49
Q8R2E9	ERO1B	1,8579E-01	0,54
P54751	SIA4A	1,8593E-01	0,32
O35874	SATT	1,8593E-01	0,32
A2AJI0	MA7D1	1,8593E-01	0,32
P27808	MGAT1	1,8593E-01	0,32
Q01237	HMDH	1,8593E-01	0,32
Q64739	COBA2	1,8593E-01	0,32
Q4QQM4	P5I11	1,8593E-01	0,32
Q3UDR8	YIPF3	1,8593E-01	0,32
Q8VCX5	MICU1	1,8593E-01	0,32
Q8R5L3	VPS39	1,8593E-01	0,32
Q9JM62	REEP6	1,8593E-01	0,32
P18155	MTDC	1,8593E-01	0,32
Q9ERY9	ERG28	1,8593E-01	0,32
O54784	DAPK3	1,8593E-01	0,32
Q3ZK22	VEZA	1,8593E-01	0,32
Q60584	FBXW2	1,8593E-01	0,32
Q8VDS4	RPR1A	1,8593E-01	0,32
Q80ZU0	ARL5A	1,8593E-01	0,32
Q3UN04	UBP30	1,8593E-01	0,32
Q9D273	MMAB	1,8593E-01	0,32
Q922Y0	DYRK3	1,8593E-01	0,32
O88998	NOE1	1,8593E-01	0,32
Q3U0B3	DHR11	1,8593E-01	0,32
Q80WG5	LRC8A	1,8593E-01	0,32
Q9D8B7	JAM3	1,8593E-01	0,32
Q8R3C1	CB042	1,8593E-01	0,32
Q8R035	ICT1	1,8593E-01	0,32
P56213	ALR	1,8593E-01	0,32
Q8C407	YIPF4	1,8593E-01	0,32
Q56A08	GPKOW	1,8593E-01	0,32
Q91ZW9	C209C	1,8593E-01	0,32
O08919	NUMBL	1,8593E-01	0,32
O35600	ABCA4	1,8593E-01	0,32
P54763	EPHB2	1,8593E-01	0,32
Q8CBG9	RN170	1,8593E-01	0,32
Q8BTV1	TUSC3	1,8593E-01	0,32
Q8K0T0	RTN1	1,8593E-01	0,32
Q9R016	BIR1E	1,8593E-01	0,32
G3X987	GIMA9	1,8593E-01	0,32
Q9QZI9	SERC3	1,8593E-01	0,32
Q8C172	CERS6	1,8593E-01	0,32

Q8BIG2	LKAM1	1,8593E-01	0,32
Q8CD54	PIEZ2	1,8593E-01	0,32
Q6NVE8	WDR44	1,8593E-01	0,32
O70200	AIF1	1,8593E-01	0,32
Q8R4Y4	STAB1	1,8593E-01	0,32
Q3TC33	CC127	1,8593E-01	0,32
B1AZP2	DLGP4	1,8593E-01	0,32
Q80UG1	FADS6	1,8593E-01	0,32
O55026	ENTP2	1,8593E-01	0,32
O88829	SIAT9	1,8593E-01	0,32
Q8C0Q2	ZHX3	1,8593E-01	0,32
Q6PD19	ARMD3	1,8593E-01	0,32
Q8CI08	SLAI2	1,8593E-01	0,32
Q9D7B1	DUS2L	1,8593E-01	0,32
Q9D2Z4	SENP8	1,8593E-01	0,32
Q8K1C0	ANGE2	1,8593E-01	0,32
Q8VE99	CC115	1,8593E-01	0,32
Q9DBX3	SUSD2	1,8593E-01	0,32
Q61234	SNTA1	1,8593E-01	0,32
Q9CX83	ARMX1	1,8593E-01	0,32
Q6P2L6	NSD3	1,8593E-01	0,32
A2AKB4	FRPD1	1,8593E-01	0,32
P0CW02	LY6C1	1,8593E-01	0,32
Q8C0L8	COG5	1,8593E-01	0,32
Q91UZ5	IMPA2	1,8593E-01	0,32
Q8VCD5	MED17	1,8593E-01	0,32
O88848	ARL6	1,8593E-01	0,32
O55042	SYUA	1,8593E-01	0,32
Q9DBY5	CBX6	1,8593E-01	0,32
B2RX14	TUT4	1,8593E-01	0,32
Q9DCI3	STR3N	1,8593E-01	0,32
Q8CFJ7	S2545	1,8593E-01	0,32
Q8BJT9	EDEM2	1,8593E-01	0,32
Q9CTN4	RHBT3	1,8593E-01	0,32
P33174	KIF4	1,8593E-01	0,32
Q9CQN3	TOM6	1,8593E-01	0,32
Q9DAN8	CST12	1,8593E-01	0,32
Q61391	NEP	1,8593E-01	0,32
Q78J03	MSRB2	1,8593E-01	0,32
Q9Z2X2	PSD10	1,8593E-01	0,32
Q9DCN1	NUD12	1,8593E-01	0,32
Q9JLB2	MPP5	1,8593E-01	0,32
Q8JZZ7	AGRL2	1,8593E-01	0,32

Q9CQX8	RT36	1,8593E-01	0,32
P82347	SGCD	1,8593E-01	0,32
Q5BL07	PEX1	1,8593E-01	0,32
B1AR13	CISD3	1,8593E-01	0,32
Q5U5M8	BL1S3	1,8593E-01	0,32
Q9JKL4	NDUF3	1,8593E-01	0,32
Q9WTZ0	UXT	1,8593E-01	0,32
P46062	SIPA1	1,8593E-01	0,32
Q9JLN9	MTOR	1,8593E-01	0,32
Q4FZC9	SYNE3	1,8593E-01	0,32
Q5SS00	ZDBF2	1,8593E-01	0,32
Q91VC7	PP14A	1,8593E-01	0,32
Q9CYK1	SYWM	1,8593E-01	0,32
Q9D074	MGRN1	1,8593E-01	0,32
O35678	MGLL	1,8593E-01	0,32
Q9WV96	T10B	1,8593E-01	0,32
Q9JKV5	SCAM4	1,8593E-01	0,32
Q8VBZ0	DHRSX	1,8593E-01	0,32
Q99P30	NUDT7	1,8593E-01	0,32
P62257	UBE2H	1,8593E-01	0,32
Q8CHP6	PHC3	1,8593E-01	0,32
P03930	ATP8	1,8593E-01	0,32
P70663	SPRL1	1,8593E-01	0,32
Q8BWA5	KLH31	1,8593E-01	0,32
Q8CHR6	DPYD	1,8593E-01	0,32
Q91WC1	POTE1	1,8593E-01	0,32
P51830	ADCY9	1,8593E-01	0,32
E9Q6J5	BD1L1	1,8593E-01	0,32
Q9Z2C4	MTMR1	1,8593E-01	0,32
Q9D8T7	SLIRP	1,8593E-01	0,32
Q9CQG9	TM100	1,8593E-01	0,32
Q9JK24	P2R3C	1,8593E-01	0,32
Q3UMU9	HDGR2	1,8593E-01	0,32
P97348	RHOD	1,8593E-01	0,32
Q9DBR7	MYPT1	1,8595E-01	-0,57
Q9CQP3	CHCH5	1,8649E-01	0,54
Q9DC69	NDUA9	1,8680E-01	0,25
P35276	RAB3D	1,8685E-01	0,40
Q9CY18	SNX7	1,8704E-01	0,54
Q9D880	TIM50	1,8710E-01	0,25
P04186	CFAB	1,8764E-01	-0,54
Q6A026	PDS5A	1,8829E-01	-0,61
Q99LG2	TNPO2	1,8908E-01	-0,73

Q9CWL8	CTBL1	1,8934E-01	-0,68
O09044	SNP23	1,8965E-01	0,32
Q6NVF9	CPSF6	1,8965E-01	-0,64
Q9JHR7	IDE	1,8989E-01	-0,54
Q8VE97	SRSF4	1,9006E-01	0,33
P07901	HS90A	1,9063E-01	-0,30
Q8VE09	TT39C	1,9114E-01	-0,68
E9Q5C9	NOLC1	1,9114E-01	-0,68
Q7TSG2	CTDP1	1,9114E-01	-0,68
Q9JJL8	SYSM	1,9114E-01	-0,68
P13745	GSTA1	1,9133E-01	-0,70
Q8BMG7	RBGPR	1,9143E-01	0,56
O08917	FLOT1	1,9152E-01	0,28
Q61169	GATA6	1,9256E-01	-0,54
O35969	GAMT	1,9307E-01	-0,58
Q9ESJ0	XPO4	1,9405E-01	-0,83
Q99PV0	PRP8	1,9428E-01	-1,28
Q80X50	UBP2L	1,9474E-01	-0,58
P62274	RS29	1,9498E-01	-0,68
Q08288	LYAR	1,9498E-01	-0,68
Q921N6	DDX27	1,9498E-01	-0,68
Q8C460	ERI3	1,9498E-01	-0,68
Q9ESY9	GILT	1,9498E-01	-0,68
P30115	GSTA3	1,9508E-01	0,45
Q91YM4	FAKD4	1,9570E-01	-0,90
A2BE28	LAS1L	1,9571E-01	-0,64
P35564	CALX	1,9571E-01	0,05
Q99KH8	STK24	1,9575E-01	-0,54
Q8K310	MATR3	1,9633E-01	-0,44
Q9WVG5	LIPE	1,9670E-01	0,74
Q9Z315	SNUT1	1,9739E-01	-0,82
P48962	ADT1	1,9740E-01	0,11
Q9DCL9	PUR6	1,9777E-01	-0,47
Q91XD7	CREL1	1,9839E-01	0,39
Q8CIB5	FERM2	1,9860E-01	-0,40
Q9EP71	RAI14	1,9881E-01	-1,06
P62484	ABI2	1,9887E-01	0,54
Q6PER3	MARE3	1,9890E-01	0,51
P05202	AATM	1,9924E-01	0,07
Q9ET22	DPP2	1,9953E-01	0,28
P37804	TAGL	1,9966E-01	-0,39
P27641	XRCC5	2,0014E-01	-0,64
Q9Z1N6	SFRP4	2,0025E-01	0,39

Q9CY64	BIEA	2,0030E-01	-0,55
D3Z7P3	GLSK	2,0067E-01	-0,51
P58771	TPM1	2,0076E-01	-0,45
Q99JT2	STK26	2,0134E-01	-0,60
Q3V1L4	5NTC	2,0166E-01	-0,68
P97346	NXN	2,0209E-01	0,39
Q8BJ71	NUP93	2,0233E-01	-0,44
Q99LP6	GRPE1	2,0238E-01	0,28
Q91VX2	UBAP2	2,0289E-01	-0,82
P47955	RLA1	2,0314E-01	-0,74
P09103	PDIA1	2,0350E-01	-0,02
O08586	PTEN	2,0431E-01	0,51
Q9ESL4	M3K20	2,0508E-01	0,40
Q3UV70	PDP1	2,0544E-01	0,40
Q8VHR5	P66B	2,0578E-01	-0,58
Q99NB1	ACS2L	2,0579E-01	0,21
O70503	DHB12	2,0648E-01	0,22
Q8BGA5	KRR1	2,0669E-01	-0,68
Q9JIM1	S29A1	2,0716E-01	0,51
Q5F2E8	TAOK1	2,0716E-01	0,51
P60670	NPL4	2,0721E-01	-0,52
Q6P9Q6	FKB15	2,0743E-01	-0,77
P58742	AAAS	2,0827E-01	-0,63
P54729	NUB1	2,0846E-01	0,32
Q8BFQ4	WDR82	2,0894E-01	-0,63
O35215	DOPD	2,0938E-01	0,28
P29416	HEXA	2,0984E-01	-0,60
Q8CFI7	RPB2	2,1000E-01	-0,68
C0HKE1	H2A1B	2,1028E-01	-0,63
P29758	OAT	2,1041E-01	0,03
P49722	PSA2	2,1065E-01	-0,54
Q8JZN7	MIRO2	2,1119E-01	0,26
P97855	G3BP1	2,1144E-01	-0,44
P20108	PRDX3	2,1167E-01	0,15
Q1HFZ0	NSUN2	2,1180E-01	-0,48
Q9DCZ4	MIC26	2,1196E-01	0,40
Q61247	A2AP	2,1228E-01	-0,72
Q9D6S7	RRFM	2,1251E-01	0,51
Q4VBD2	TAPT1	2,1253E-01	0,54
Q9CZ42	NNRD	2,1310E-01	0,20
Q9D7S7	RL22L	2,1330E-01	0,40
Q04447	KCRB	2,1371E-01	-0,36
Q6A028	SWP70	2,1436E-01	-0,68

Q8BHC9	FUT11	2,1482E-01	-0,68
Q8C4Y3	NELFB	2,1485E-01	-0,68
Q65Z40	WAPL	2,1504E-01	-0,85
O55222	ILK	2,1524E-01	-0,48
Q922Q9	CHID1	2,1527E-01	0,40
Q8CEC0	NUP88	2,1539E-01	-0,68
Q8BG05	ROA3	2,1549E-01	0,01
P23198	CBX3	2,1554E-01	0,18
Q63850	NUP62	2,1604E-01	-0,68
P21619	LMNB2	2,1684E-01	0,04
Q9JHU9	INO1	2,1687E-01	-0,44
Q921W4	QORL1	2,1689E-01	-0,60
P23492	PNPH	2,1737E-01	0,19
Q8BFS6	CPPED	2,1783E-01	-0,68
Q9D0S9	HINT2	2,1802E-01	0,40
Q80YV2	NIPA	2,1808E-01	-0,63
Q00915	RET1	2,1819E-01	0,15
P45878	FKBP2	2,1835E-01	0,40
P54731	FAF1	2,1874E-01	-0,63
Q9CZ28	SNF8	2,1924E-01	-0,68
Q9QXK7	CPSF3	2,1925E-01	-0,63
Q8BWZ3	NAA25	2,1932E-01	-0,85
Q62384	ZPR1	2,1961E-01	0,25
Q924Z4	CERS2	2,1981E-01	-0,68
Q08093	CNN2	2,1989E-01	-0,63
Q01339	APOH	2,1992E-01	-0,53
Q3UHD6	SNX27	2,2090E-01	-0,63
P97452	BOP1	2,2096E-01	-0,68
Q7TMM9	TBB2A	2,2105E-01	-0,44
Q3UVL4	VPS51	2,2133E-01	-0,78
P02469	LAMB1	2,2154E-01	-0,55
Q8K1B8	URP2	2,2161E-01	0,79
P97351	RS3A	2,2164E-01	0,08
Q8BGC0	HTSF1	2,2181E-01	-0,63
Q3UMY5	EMAL4	2,2215E-01	-0,63
P52019	ERG1	2,2279E-01	0,41
Q9WU28	PFD5	2,2303E-01	-0,68
Q91VE6	MK67I	2,2303E-01	-0,68
P03987	IGHG3	2,2303E-01	-0,68
P33587	PROC	2,2303E-01	-0,68
Q9D2E2	TOE1	2,2303E-01	-0,68
P11087	CO1A1	2,2324E-01	0,28
Q9Z0U1	ZO2	2,2348E-01	0,51

Q8BJU2	TSN9	2,2348E-01	0,51
Q61420	S35A1	2,2348E-01	0,51
P02088	HBB1	2,2369E-01	0,41
P14824	ANXA6	2,2376E-01	-0,02
Q9ERD7	TBB3	2,2443E-01	0,06
P24270	CATA	2,2458E-01	0,04
O55023	IMPA1	2,2466E-01	0,13
P45952	ACADM	2,2471E-01	0,11
Q9JHW4	SELB	2,2517E-01	-0,68
Q924M7	MPI	2,2543E-01	0,22
P97864	CASP7	2,2560E-01	-0,68
A2RSY6	TRM1L	2,2560E-01	-0,68
Q6NZC7	S23IP	2,2587E-01	0,22
P29699	FETUA	2,2640E-01	-0,44
P97494	GSH1	2,2709E-01	0,17
Q9JLJ2	AL9A1	2,2727E-01	0,04
O88792	JAM1	2,2749E-01	0,41
P15116	CADH2	2,2807E-01	-0,68
Q9D8B3	CHM4B	2,2828E-01	0,40
P26443	DHE3	2,2932E-01	0,01
Q8K0G5	EIPR1	2,2932E-01	-0,68
Q6ZQ38	CAND1	2,2975E-01	-0,36
P07309	TTHY	2,3059E-01	-0,57
Q9ERU3	ZNF22	2,3072E-01	-0,68
Q7TQK1	INT7	2,3079E-01	-0,68
Q569Z5	DDX46	2,3136E-01	-0,63
Q8BKZ9	ODPX	2,3158E-01	0,32
P29341	PABP1	2,3177E-01	-0,36
Q64314	CD34	2,3178E-01	0,42
P36371	TAP2	2,3189E-01	0,43
Q9WUK2	IF4H	2,3238E-01	-0,57
Q03141	MARK3	2,3257E-01	0,48
P12787	COX5A	2,3267E-01	0,27
Q8BM72	HSP13	2,3311E-01	-0,63
Q91ZW2	OFUT1	2,3327E-01	-0,68
Q8VEE0	RPE	2,3342E-01	-0,68
Q9EQ28	DPOD3	2,3343E-01	-0,68
P47857	PFKAM	2,3390E-01	0,20
P10605	CATB	2,3398E-01	0,07
Q3TZX3	S2533	2,3411E-01	0,49
Q9EPQ7	STAR5	2,3411E-01	0,49
Q8VCX1	AK1D1	2,3411E-01	0,49
P70288	HDAC2	2,3435E-01	-0,55

Q8BTZ4	APC5	2,3517E-01	-0,68
O08997	ATOX1	2,3517E-01	-0,68
Q5SUR0	PUR4	2,3554E-01	-0,41
Q9DC63	FBX3	2,3579E-01	-0,68
Q8R3S6	EXOC1	2,3579E-01	-0,68
P63166	SUMO1	2,3692E-01	-0,68
Q9JHS9	CWC15	2,3795E-01	0,51
Q9JIX8	ACINU	2,3797E-01	-0,51
Q8BU30	SYIC	2,3801E-01	-0,54
P70697	DCUP	2,3846E-01	0,20
Q80U95	UBE3C	2,3862E-01	-0,68
P68404	KPCB	2,3901E-01	0,50
Q2NL51	GSK3A	2,3925E-01	-0,68
Q9JIF0	ANM1	2,3947E-01	-0,36
Q9WV32	ARC1B	2,3999E-01	0,14
Q9ESD7	DYSF	2,4021E-01	0,32
Q8BG95	MYPT2	2,4045E-01	-0,67
P46978	STT3A	2,4066E-01	0,20
P61205	ARF3	2,4075E-01	0,10
Q8R079	BFAR	2,4087E-01	0,49
Q9QZS3	NUMB	2,4087E-01	0,49
Q91YE7	RBM5	2,4108E-01	-0,68
Q99KP6	PRP19	2,4142E-01	-0,43
Q9D051	ODPB	2,4173E-01	0,11
Q60648	SAP3	2,4204E-01	0,32
Q9WVQ5	MTNB	2,4207E-01	0,32
Q922Q8	LRC59	2,4244E-01	-0,51
Q9CYD3	CRTAP	2,4259E-01	0,27
Q8K2C6	SIR5	2,4284E-01	0,43
Q8K114	INT9	2,4301E-01	-0,79
Q9DB41	GHC2	2,4326E-01	0,38
Q9CWZ7	SNAG	2,4347E-01	0,32
O35855	BCAT2	2,4481E-01	0,07
P10853	H2B1F	2,4494E-01	0,08
Q501J7	PHAR4	2,4508E-01	0,49
Q9D1K2	VATF	2,4508E-01	0,49
Q8K4K6	PANK1	2,4508E-01	0,49
Q9CQX2	CYB5B	2,4541E-01	0,19
Q3URQ0	TEX10	2,4592E-01	-1,06
Q5RL79	KTAP2	2,4637E-01	0,47
P34022	RANG	2,4642E-01	-0,62
P13595	NCAM1	2,4660E-01	0,42
Q9QZH6	ECSIT	2,4670E-01	0,51

P63017	HSP7C	2,4693E-01	-0,03
Q8K284	TF3C1	2,4763E-01	-0,49
P33215	NEDD1	2,4763E-01	-0,49
Q80TE0	RPAP1	2,4763E-01	-0,49
Q9CWY8	RNH2A	2,4763E-01	-0,49
Q8C5L3	CNOT2	2,4763E-01	-0,49
Q9DBA9	TF2H1	2,4763E-01	-0,49
P01636	KV5A4	2,4763E-01	-0,49
Q80TP3	UBR5	2,4763E-01	-0,49
Q8R5C8	ZMY11	2,4763E-01	-0,49
Q61810	LTBP3	2,4763E-01	-0,49
Q8BJ56	PLPL2	2,4763E-01	-0,49
Q8BRN9	C2D1B	2,4763E-01	-0,49
Q5I043	UBP28	2,4763E-01	-0,49
Q8BJL0	SMAL1	2,4763E-01	-0,49
Q5M8N4	D39U1	2,4763E-01	-0,49
P48755	FOSL1	2,4763E-01	-0,49
Q61749	EI2BD	2,4773E-01	0,44
P47739	AL3A1	2,4835E-01	0,32
Q8VDM6	HNRL1	2,4852E-01	-0,49
P35601	RFC1	2,4942E-01	-0,68
Q8K4Q0	RPTOR	2,4942E-01	-0,68
Q8C050	KS6A5	2,4942E-01	-0,68
P01639	KV5A7	2,4942E-01	-0,68
Q6X893	CTL1	2,4942E-01	-0,68
Q8C156	CND2	2,4942E-01	-0,68
P83741	WNK1	2,4942E-01	-0,68
Q497V5	SRBD1	2,4942E-01	-0,68
Q6PE54	DHX40	2,4942E-01	-0,68
Q9CXE7	TMED5	2,4957E-01	0,47
Q9WUT3	KS6A2	2,4962E-01	0,32
Q9D0L4	ADCK1	2,4978E-01	0,46
Q8K2I3	FMO2	2,4978E-01	0,46
Q9D0L8	MCES	2,4979E-01	-0,51
P37913	DNLI1	2,4982E-01	-0,85
Q6PF93	PK3C3	2,4982E-01	-0,85
Q9Z0H1	WDR46	2,4982E-01	-0,85
Q80X19	COEA1	2,4982E-01	-0,85
Q9R0I7	YLPM1	2,4992E-01	-1,00
Q9DBE9	SPB1	2,4993E-01	-1,14
Q8R4X3	RBM12	2,5007E-01	-0,62
P97376	FRG1	2,5017E-01	0,45
P48036	ANXA5	2,5025E-01	-0,35

P35290	RAB24	2,5030E-01	0,43
Q80TI0	ASTRB	2,5064E-01	0,24
P06683	CO9	2,5081E-01	-0,82
Q3UM45	PP1R7	2,5086E-01	-0,45
O70305	ATX2	2,5108E-01	0,47
O35566	CD151	2,5108E-01	0,47
P13707	GPDA	2,5139E-01	0,31
P70257	NFIX	2,5170E-01	-0,66
O88487	DC1I2	2,5176E-01	-0,47
Q9CQJ4	RING2	2,5183E-01	-0,65
P37040	NCPR	2,5186E-01	0,02
P58137	ACOT8	2,5203E-01	0,32
Q922S4	PDE2A	2,5234E-01	0,46
O55242	SGMR1	2,5269E-01	0,44
P18531	HVM60	2,5288E-01	0,45
Q925N0	SFXN5	2,5288E-01	0,45
Q8BHI7	ELOV5	2,5288E-01	0,45
P56382	ATP5E	2,5330E-01	0,46
Q9R069	BCAM	2,5331E-01	0,19
Q9WUQ2	PREB	2,5372E-01	0,32
P01646	KV5AD	2,5393E-01	-0,31
Q6PHZ2	KCC2D	2,5407E-01	-0,54
P58196	PLS4	2,5417E-01	0,47
Q99JR5	TINAL	2,5419E-01	-0,58
Q91WS0	CISD1	2,5435E-01	0,46
Q8CC35	SYNPO	2,5437E-01	-0,62
Q923T9	KCC2G	2,5463E-01	0,18
Q9DBE8	ALG2	2,5510E-01	0,24
Q9D892	ITPA	2,5602E-01	-0,56
P16015	CAH3	2,5605E-01	-0,85
Q99KP3	CRYL1	2,5652E-01	-0,42
Q9D7M8	RPB4	2,5677E-01	-0,49
Q61646	HPT	2,5677E-01	-0,49
Q9QZB9	DCTN5	2,5677E-01	-0,49
P22935	RABP2	2,5677E-01	-0,49
P28700	RXRA	2,5677E-01	-0,49
Q8R0J7	VP37B	2,5677E-01	-0,49
Q8JZQ2	AFG32	2,5695E-01	0,21
Q9Z1R2	BAG6	2,5706E-01	-0,68
P11404	FABPH	2,5718E-01	0,27
Q61495	DSG1A	2,5737E-01	-0,68
P49615	CDK5	2,5743E-01	0,48
Q8VDV3	R3GEF	2,5747E-01	0,47

Q9CZD3	GARS	2,5748E-01	-0,44
Q9EPB4	ASC	2,5789E-01	-0,68
Q8BY71	HAT1	2,5865E-01	-0,62
Q64310	SURF4	2,5888E-01	0,21
Q9Z2H5	E41L1	2,5909E-01	-0,62
Q8BI72	CARF	2,5955E-01	-0,62
Q8VCI5	PEX19	2,5985E-01	0,27
Q99KI0	ACON	2,6008E-01	-0,03
P97350	PKP1	2,6012E-01	-0,49
Q08189	TGM3	2,6012E-01	-0,49
Q69ZI1	SH3R1	2,6012E-01	-0,49
Q8CCN5	BCAS3	2,6036E-01	-0,49
Q8CG19	LTBP1	2,6036E-01	-0,49
Q3TRM4	PLPL6	2,6036E-01	-0,49
Q60932	VDAC1	2,6041E-01	0,05
Q8R4R6	NUP35	2,6048E-01	-0,56
Q9ERG0	LIMA1	2,6050E-01	0,32
Q99LE6	ABCF2	2,6164E-01	0,21
Q8CJG0	AGO2	2,6172E-01	-0,62
Q6NSQ9	G6PC3	2,6206E-01	0,42
Q9CR57	RL14	2,6227E-01	-0,56
P11928	OAS1A	2,6257E-01	-0,49
P05132	KAPCA	2,6264E-01	-0,56
Q3U186	SYRM	2,6299E-01	-0,68
Q91VJ4	STK38	2,6357E-01	-0,61
Q7TMF3	NDUAC	2,6379E-01	0,27
Q9DCS3	MECR	2,6393E-01	0,23
Q62000	MIME	2,6397E-01	0,23
Q3UHJ0	AAK1	2,6404E-01	-1,12
Q7TMI3	UHRF2	2,6407E-01	-0,30
Q61316	HSP74	2,6427E-01	-0,31
P17751	TPIS	2,6477E-01	0,02
P62830	RL23	2,6487E-01	0,19
Q8C1Y8	CCZ1	2,6499E-01	0,46
Q8VI64	HUMMR	2,6511E-01	0,19
Q8VEJ9	VPS4A	2,6548E-01	0,47
P35951	LDLR	2,6580E-01	-0,90
Q8BMA6	SRP68	2,6595E-01	0,10
E9Q1P8	I2BP2	2,6657E-01	-0,63
P61750	ARF4	2,6696E-01	0,15
Q8CGY8	OGT1	2,6723E-01	-0,51
P58044	IDI1	2,6755E-01	0,14
Q9DC48	PRP17	2,6770E-01	-0,57

P08249	MDHM	2,6772E-01	0,00
Q80X90	FLNB	2,6791E-01	0,03
Q91V01	MBOA5	2,6821E-01	0,27
P70698	PYRG1	2,6848E-01	0,12
Q91X78	ERLN1	2,6891E-01	-0,46
Q8BHG1	NRDC	2,6897E-01	-0,80
Q9DCV4	RMD1	2,6993E-01	0,32
P62281	RS11	2,7009E-01	0,14
Q62432	SMAD2	2,7057E-01	-0,90
Q64518	AT2A3	2,7074E-01	0,32
P47856	GFPT1	2,7116E-01	-0,47
Q02013	AQP1	2,7218E-01	0,32
P43277	H13	2,7442E-01	-0,49
Q9DCC4	P5CR3	2,7495E-01	0,18
P55772	ENTP1	2,7502E-01	0,32
Q9D6Y7	MSRA	2,7506E-01	0,27
Q8JZQ9	EIF3B	2,7508E-01	-0,38
P53395	ODB2	2,7663E-01	0,15
P97930	KTHY	2,7682E-01	-0,61
P84084	ARF5	2,7767E-01	0,20
Q8C0C7	SYFA	2,7770E-01	0,15
Q8CI11	GNL3	2,7771E-01	-0,58
Q9Z247	FKBP9	2,7981E-01	0,44
Q9CQQ7	AT5F1	2,7987E-01	-0,50
P51661	DHI2	2,8027E-01	0,32
Q06185	ATP5I	2,8035E-01	0,32
Q64191	ASPG	2,8071E-01	-0,60
Q99J45	NRBP	2,8088E-01	-0,60
P51881	ADT2	2,8194E-01	0,04
Q8C0N1	KIF2B	2,8278E-01	-0,28
P46737	BRCC3	2,8284E-01	0,32
Q9CYH2	PXL2A	2,8294E-01	0,20
Q61166	MARE1	2,8336E-01	-0,42
Q9JI46	NUDT3	2,8378E-01	0,58
Q8BFR5	EFTU	2,8423E-01	-0,02
Q922D4	PP6R3	2,8429E-01	-0,54
Q9D855	QCR7	2,8450E-01	0,23
Q61559	FCGRN	2,8537E-01	0,32
Q9QYJ0	DNJA2	2,8698E-01	0,14
Q8CAY6	THIC	2,8723E-01	0,14
Q9DBC7	KAP0	2,8762E-01	0,15
Q9CRY7	GDPD1	2,8831E-01	-0,60
Q8BPU7	ELMO1	2,8866E-01	-0,57

P61161	ARP2	2,8910E-01	0,09
Q8K268	ABCF3	2,9037E-01	-0,60
P45376	ALDR	2,9071E-01	0,02
Q3UQ44	IQGA2	2,9141E-01	0,18
Q920A7	AFG31	2,9167E-01	-0,27
Q6ZQ08	CNOT1	2,9186E-01	-0,60
Q8K3G9	DP13B	2,9199E-01	-0,42
Q91VT4	CBR4	2,9201E-01	0,32
O88895	HDAC3	2,9335E-01	-0,27
Q8K1X1	WDR11	2,9392E-01	-0,88
P01029	CO4B	2,9401E-01	-0,53
Q62077	PLCG1	2,9417E-01	-0,68
A2A8Z1	OSBL9	2,9442E-01	0,26
Q62523	ZYX	2,9478E-01	-0,44
Q9DB60	PXL2B	2,9489E-01	0,32
Q99NB9	SF3B1	2,9489E-01	-0,47
Q32Q92	ACOT6	2,9519E-01	0,31
Q9DBD5	PELP1	2,9532E-01	-0,54
Q62422	OSTF1	2,9600E-01	0,26
Q05CL8	LARP7	2,9644E-01	-0,60
P11688	ITA5	2,9654E-01	-0,60
P32020	NLTP	2,9658E-01	0,12
Q8BJD1	ITIH5	2,9658E-01	0,51
P26043	RADI	2,9682E-01	-0,35
P42125	ECI1	2,9707E-01	0,08
Q99LY9	NDUS5	2,9761E-01	0,32
O55234	PSB5	2,9779E-01	0,12
Q99LI2	CLCC1	2,9858E-01	0,32
P18572	BASI	2,9881E-01	-0,49
Q80YF6	UPK3B	2,9892E-01	-0,26
P14847	CRP	2,9892E-01	-0,26
Q64339	ISG15	2,9892E-01	-0,26
Q8CA72	GAN	2,9892E-01	-0,26
Q9QY24	ZBP1	2,9892E-01	-0,26
P70180	ANPRC	2,9892E-01	-0,26
Q8R242	DIAC	2,9892E-01	-0,26
Q9JI13	SAS10	2,9892E-01	-0,26
Q8BHJ9	SLU7	2,9892E-01	-0,26
O08784	TCOF	2,9892E-01	-0,26
Q9D8M4	RL7L	2,9892E-01	-0,26
Q61730	IL1AP	2,9892E-01	-0,26
Q9CR41	HYPK	2,9892E-01	-0,26
Q05A36	MEX3C	2,9892E-01	-0,26

Q8C0W1	ANMY1	2,9892E-01	-0,26
Q64345	IFIT3	2,9892E-01	-0,26
Q8C0D0	TRUB1	2,9892E-01	-0,26
P20352	TF	2,9892E-01	-0,26
P0DN34	NDUB1	2,9892E-01	-0,26
P23188	FURIN	2,9892E-01	-0,26
Q66JV4	R12BB	2,9892E-01	-0,26
Q7TND5	RPF1	2,9892E-01	-0,26
Q71FD5	ZNRF2	2,9892E-01	-0,26
Q80US4	ARP5	2,9892E-01	-0,26
Q9D1P2	KAT8	2,9892E-01	-0,26
Q6A0D4	RFTN1	2,9892E-01	-0,26
P36423	THAS	2,9892E-01	-0,26
Q8C0D4	RHG12	2,9892E-01	-0,26
Q9CQL5	RM18	2,9892E-01	-0,26
Q8BGB8	COQ4	2,9892E-01	-0,26
Q9EPQ2	RPGR1	2,9892E-01	-0,26
Q6PAQ4	REXO4	2,9892E-01	-0,26
Q5SQM0	EMAL6	2,9892E-01	-0,26
Q8BQM8	EMAL5	2,9892E-01	-0,26
P17809	GTR1	2,9892E-01	-0,26
Q9D009	LIPT2	2,9892E-01	-0,26
Q8K1L5	PP1RB	2,9892E-01	-0,26
Q8K4L4	POF1B	2,9892E-01	-0,26
O88413	TULP3	2,9892E-01	-0,26
P00158	CYB	2,9892E-01	-0,26
Q9ERS5	PKHA2	2,9892E-01	-0,26
P28828	PTPRM	2,9892E-01	-0,26
Q9CWX2	CIA30	2,9892E-01	-0,26
Q9Z0Y7	IRS4	2,9892E-01	-0,26
O88597	BECN1	2,9892E-01	-0,26
Q8VE22	RT23	2,9892E-01	-0,26
Q9D2R0	AACS	2,9892E-01	-0,26
Q8R5A0	SMYD2	2,9892E-01	-0,26
Q8BTZ5	ANR46	2,9892E-01	-0,26
Q9CZB3	THUM2	2,9892E-01	-0,26
Q9WTQ5	AKA12	2,9892E-01	-0,26
Q99NF2	NSMF	2,9892E-01	-0,26
Q9DBX1	RGCC	2,9892E-01	-0,26
Q9Z175	LOXL3	2,9892E-01	-0,26
P28650	PURA1	2,9892E-01	-0,26
Q8BVW0	GANC	2,9892E-01	-0,26
Q9CUN6	SMUF1	2,9892E-01	-0,26

Q9D0L1	ZBED3	2,9892E-01	-0,26
Q91VX9	TM168	2,9892E-01	-0,26
Q9D0K1	PEX13	2,9892E-01	-0,26
Q80WT5	AFTIN	2,9892E-01	-0,26
Q1HCM0	FGFP3	2,9892E-01	-0,26
P59672	ANS1A	2,9892E-01	-0,26
Q9DCJ9	NPL	2,9892E-01	-0,26
Q6DFV7	NCOA7	2,9892E-01	-0,26
Q7TN29	SMAP2	2,9892E-01	-0,26
Q3UDK1	TRAD1	2,9892E-01	-0,26
Q80TL7	MON2	2,9892E-01	-0,26
Q6P3E7	HDA10	2,9892E-01	-0,26
Q91WQ5	TAF5L	2,9892E-01	-0,26
Q8C4S8	DEN2A	2,9892E-01	-0,26
Q8CGY6	UN45B	2,9892E-01	-0,26
Q99MR1	GGYF1	2,9892E-01	-0,26
Q9QZN0	FBX15	2,9892E-01	-0,26
Q9WTK5	NFKB2	2,9892E-01	-0,26
Q9Z2U2	ZN292	2,9892E-01	-0,26
Q91YM2	RHG35	2,9892E-01	-0,26
Q3TRJ4	K1C26	2,9892E-01	-0,26
Q9D132	UPK1A	2,9892E-01	-0,26
Q80SU3	ZAR1	2,9892E-01	-0,26
Q99LJ5	CKLF3	2,9892E-01	-0,26
P70206	PLXA1	2,9892E-01	-0,26
Q9DAM7	TM263	2,9892E-01	-0,26
Q9JII1	INP5E	2,9892E-01	-0,26
Q60989	XIAP	2,9892E-01	-0,26
Q8CIV8	TBCE	2,9892E-01	-0,26
Q80ZM7	T2AG	2,9892E-01	-0,26
Q8BVG4	DPP9	2,9892E-01	-0,26
Q8K1A6	C2D1A	2,9892E-01	-0,26
Q8BFW4	TRI65	2,9892E-01	-0,26
Q09143	CTR1	2,9892E-01	-0,26
Q9D0Q7	RM45	2,9892E-01	-0,26
P52482	UB2E1	2,9892E-01	-0,26
Q8R123	FAD1	2,9892E-01	-0,26
Q499X9	SYMM	2,9892E-01	-0,26
Q6PDH0	PHLB1	2,9892E-01	-0,26
Q8CDM8	F16B1	2,9892E-01	-0,26
Q60780	GAS7	2,9892E-01	-0,26
Q3UKC1	TAXB1	2,9892E-01	-0,26
Q64434	PTK6	2,9892E-01	-0,26

Q04692	SMRCD	2,9892E-01	-0,26
Q9CQ45	NENF	2,9892E-01	-0,26
O35744	CHIL3	2,9892E-01	-0,26
Q5SXA9	KIBRA	2,9892E-01	-0,26
P11152	LIPL	2,9892E-01	-0,26
Q3UUG6	TBC24	2,9892E-01	-0,26
Q8BGS1	E41L5	2,9892E-01	-0,26
Q8BGU5	CCNY	2,9892E-01	-0,26
Q8VE10	NAA40	2,9892E-01	-0,26
Q810B8	SLIK4	2,9892E-01	-0,26
Q8K3A0	HSC20	2,9892E-01	-0,26
P49891	ST1E1	2,9892E-01	-0,26
Q99J56	DERL1	2,9892E-01	-0,26
Q9JJ11	TACC3	2,9892E-01	-0,26
Q62470	ITA3	2,9892E-01	-0,26
Q99LH2	PTSS1	2,9892E-01	-0,26
P01680	KV4A1	2,9892E-01	-0,26
Q6NZR2	MSD2	2,9892E-01	-0,26
P18828	SDC1	2,9892E-01	-0,26
Q9EST4	PSMG2	2,9892E-01	-0,26
P83093	STIM2	2,9892E-01	-0,26
Q60866	PTER	2,9892E-01	-0,26
Q9CR60	GOT1B	2,9892E-01	-0,26
Q8VCS0	PGRP2	2,9892E-01	-0,26
Q810B9	SLIK3	2,9892E-01	-0,26
Q9EPL4	METL9	2,9892E-01	-0,26
Q91XL9	OSBL1	2,9892E-01	-0,26
P15307	REL	2,9892E-01	-0,26
Q8BGF3	WDR92	2,9892E-01	-0,26
Q9D7K2	TEN1L	2,9892E-01	-0,26
Q91V04	TRAM1	2,9892E-01	-0,26
Q9QXY9	PEX3	2,9892E-01	-0,26
Q8VDS8	STX18	2,9892E-01	-0,26
Q8BQZ4	RLGPB	2,9892E-01	-0,26
P70414	NAC1	2,9892E-01	-0,26
P70353	NFYC	2,9892E-01	-0,26
Q8K4P0	WDR33	2,9892E-01	-0,26
Q99K74	MED24	2,9892E-01	-0,26
Q9CR70	LAGE3	2,9892E-01	-0,26
Q8CG70	P3H3	2,9892E-01	-0,26
Q6DYE8	ENPP3	2,9892E-01	-0,26
Q8C7H1	MMAA	2,9892E-01	-0,26
Q61216	MRE11	2,9892E-01	-0,26

Q3U0J8	TBD2B	2,9892E-01	-0,26
O88668	CREG1	2,9892E-01	-0,26
Q9D1Z3	ACKMT	2,9892E-01	-0,26
A2AAY5	SPD2B	2,9892E-01	-0,26
Q9Z1E4	GYS1	2,9892E-01	-0,26
Q9CY16	RT28	2,9892E-01	-0,26
Q8BMD6	TM260	2,9892E-01	-0,26
O88286	WIZ	2,9892E-01	-0,26
Q6WKZ8	UBR2	2,9892E-01	-0,26
Q8CIV2	MBRL	2,9892E-01	-0,26
Q8VBV3	EXOS2	2,9892E-01	-0,26
Q8CB77	ELOA1	2,9892E-01	-0,26
Q8CD15	RIOX2	2,9892E-01	-0,26
Q99MS7	EH1L1	2,9892E-01	-0,26
Q9CWR2	SMYD3	2,9892E-01	-0,26
Q8BJW5	NOL11	2,9892E-01	-0,26
Q7TMR0	PCP	2,9892E-01	-0,26
Q9Z1X2	PTSS2	2,9892E-01	-0,26
Q5SUE8	ANR40	2,9892E-01	-0,26
Q4FZF3	DDX49	2,9892E-01	-0,26
Q9CR95	NECP1	2,9892E-01	-0,26
Q91WC9	DGLB	2,9892E-01	-0,26
Q8R2Q4	RRF2M	2,9892E-01	-0,26
Q8BGA7	CG026	2,9892E-01	-0,26
O35613	DAXX	2,9892E-01	-0,26
Q00262	STX2	2,9892E-01	-0,26
Q8BUY8	GASP2	2,9892E-01	-0,26
E9Q3L2	PI4KA	2,9892E-01	-0,26
O35134	RPA1	2,9892E-01	-0,26
Q8BUR4	DOCK1	2,9892E-01	-0,26
Q9CR25	DPH2	2,9892E-01	-0,26
Q9D0V7	RCAS1	2,9892E-01	-0,26
Q62313	TGON1	2,9892E-01	-0,26
Q61124	CLN3	2,9892E-01	-0,26
Q921Q3	ALG1	2,9892E-01	-0,26
Q9JJC6	RIPL1	2,9892E-01	-0,26
Q8BGZ2	F168A	2,9892E-01	-0,26
Q9R0B6	LAMC3	2,9892E-01	-0,26
Q9D1C9	RRP7A	2,9892E-01	-0,26
Q9D2X5	SCC4	2,9892E-01	-0,26
Q9WUX5	MRVI1	2,9892E-01	-0,26
Q9CPR5	RM15	2,9892E-01	-0,26
Q8VCM4	LIPT	2,9892E-01	-0,26

Q8R2N2	UTP4	2,9892E-01	-0,26
Q9R045	ANGL2	2,9892E-01	-0,26
Q9CQ28	DPH6	2,9892E-01	-0,26
Q9EPK2	XRP2	2,9892E-01	-0,26
Q9CQ02	COMD4	2,9892E-01	-0,26
Q6ZPK0	PF21A	2,9892E-01	-0,26
P48441	IDUA	2,9892E-01	-0,26
Q9CZ15	PSF1	2,9892E-01	-0,26
Q9D868	PPIH	2,9892E-01	-0,26
A6H5Z3	EXC6B	2,9892E-01	-0,26
Q3UFM5	NOM1	2,9892E-01	-0,26
P56671	MAZ	2,9892E-01	-0,26
O70496	CLCN7	2,9892E-01	-0,26
P53564	CUX1	2,9892E-01	-0,26
Q6P4S8	INT1	2,9892E-01	-0,26
Q9CQL0	MT21A	2,9892E-01	-0,26
A2AJK6	CHD7	2,9892E-01	-0,26
Q70FJ1	AKAP9	2,9892E-01	-0,26
Q9CQX5	CLDN1	2,9892E-01	-0,26
Q80V70	MEGF6	2,9892E-01	-0,26
Q9QYC7	VKGC	2,9892E-01	-0,26
Q9EQN3	T22D4	2,9892E-01	-0,26
Q9R269	PEPL	2,9892E-01	-0,26
Q8BTY8	SCFD2	2,9892E-01	-0,26
Q9CXJ1	SYEM	2,9892E-01	-0,26
Q6ZPJ3	UBE2O	2,9892E-01	-0,26
Q61214	DYR1A	2,9892E-01	-0,26
Q8CHU3	EPN2	2,9892E-01	-0,26
P97360	ETV6	2,9892E-01	-0,26
Q9D394	RUFY3	2,9892E-01	-0,26
Q8VCH8	UBXN4	2,9892E-01	-0,26
Q6PAM0	AAKB2	2,9892E-01	-0,26
Q8CG14	CS1A	2,9892E-01	-0,26
Q6P5E6	GGA2	2,9892E-01	-0,26
Q9JJH1	RNAS4	2,9892E-01	-0,26
P70175	DLG3	2,9892E-01	-0,26
Q8BXA1	GOLI4	2,9892E-01	-0,26
Q8K2Z4	CND1	2,9892E-01	-0,26
Q99JT9	MTND	2,9892E-01	-0,26
Q91VL8	TE2IP	2,9892E-01	-0,26
Q3SXD3	HDDC2	2,9892E-01	-0,26
Q8C0P7	ZN451	2,9892E-01	-0,26
Q8BPS4	GP180	2,9892E-01	-0,26

Q91WD1	RPC4	2,9892E-01	-0,26
Q8C3P7	MTA70	2,9892E-01	-0,26
P38585	TTL	2,9892E-01	-0,26
Q8BMI0	FBX38	2,9892E-01	-0,26
Q80Y55	BSDC1	2,9892E-01	-0,26
Q925J9	MED1	2,9892E-01	-0,26
Q6A0A2	LAR4B	2,9892E-01	-0,26
Q922Q2	RIOK1	2,9892E-01	-0,26
A2AIV2	VIR	2,9892E-01	-0,26
Q8R0I0	ACE2	2,9892E-01	-0,26
Q8VDH1	FBX21	2,9892E-01	-0,26
Q5XJY4	PARL	2,9892E-01	-0,26
Q99MR3	S12A9	2,9892E-01	-0,26
P24860	CCNB1	2,9892E-01	-0,26
Q8BMQ3	BNC2	2,9892E-01	-0,26
P16951	ATF2	2,9892E-01	-0,26
Q9DCU6	RM04	2,9892E-01	-0,26
Q8BJL1	FBX30	2,9892E-01	-0,26
P23950	TISB	2,9892E-01	-0,26
Q8BHT6	B3GLT	2,9892E-01	-0,26
Q80XC2	TRM61	2,9892E-01	-0,26
Q8R366	IGSF8	2,9892E-01	-0,26
Q99LG4	TTC5	2,9892E-01	-0,26
O08808	DIAP1	2,9892E-01	-0,26
Q91W96	APC4	2,9892E-01	-0,26
Q64705	USF2	2,9892E-01	-0,26
Q9CQ26	STABP	2,9892E-01	-0,26
Q9D7E3	OVCA2	2,9892E-01	-0,26
Q924W7	ST5	2,9892E-01	-0,26
Q9Z2E2	MBD1	2,9892E-01	-0,26
B1ARD6	SLFN9	2,9892E-01	-0,26
B7ZNG0	KIF7	2,9892E-01	-0,26
P0DN89	T254A	2,9892E-01	-0,26
Q8K202	RPA49	2,9892E-01	-0,26
P28704	RXRB	2,9892E-01	-0,26
Q8BZH4	POGZ	2,9892E-01	-0,26
Q91VM3	WIPI4	2,9892E-01	-0,26
Q5U3K5	RABL6	2,9892E-01	-0,26
Q9CQ71	RFA3	2,9892E-01	-0,26
Q99LS3	SERB	2,9892E-01	-0,26
Q9DB28	POP5	2,9892E-01	-0,26
Q80W68	KIRR1	2,9892E-01	-0,26
Q8BU40	NAL4A	2,9892E-01	-0,26

Q924Z5	TRAM2	2,9892E-01	-0,26
P70429	EVL	2,9892E-01	-0,26
Q8CGB3	UACA	2,9892E-01	-0,26
Q8K1H1	TDRD7	2,9892E-01	-0,26
Q9CQK7	RWDD1	2,9892E-01	-0,26
Q3TSG4	ALKB5	2,9892E-01	-0,26
O55033	NCK2	2,9892E-01	-0,26
P47931	FST	2,9892E-01	-0,26
Q91YX5	LGAT1	2,9892E-01	-0,26
Q91ZM2	SH2B1	2,9892E-01	-0,26
Q9Z2Q2	KNOP1	2,9892E-01	-0,26
Q6NVF0	OCRL	2,9892E-01	-0,26
Q8R3K3	PTCD2	2,9892E-01	-0,26
Q921J4	UBE2S	2,9892E-01	-0,26
Q6IRU5	CLCB	2,9892E-01	-0,26
Q9D1H9	MFAP4	2,9892E-01	-0,26
Q62311	TAF6	2,9892E-01	-0,26
Q6NS82	RETR2	2,9892E-01	-0,26
Q8BT14	CNOT4	2,9892E-01	-0,26
P20491	FCERG	2,9892E-01	-0,26
Q9DB30	PHKG2	2,9892E-01	-0,26
Q64373	B2CL1	2,9892E-01	-0,26
Q8BIP0	SYDM	2,9892E-01	-0,26
P70704	AT8A1	2,9892E-01	-0,26
Q9CZ49	KLH35	2,9892E-01	-0,26
Q9D4V0	EKI1	2,9892E-01	-0,26
Q9D1I5	MCEE	2,9892E-01	-0,26
Q60675	LAMA2	2,9892E-01	-0,26
P61406	EST1A	2,9892E-01	-0,26
O35425	BOK	2,9892E-01	-0,26
Q9JIA7	SPHK2	2,9892E-01	-0,26
P30051	TEAD1	2,9892E-01	-0,26
A2APY7	NDUF5	2,9892E-01	-0,26
Q8CE96	TRM6	2,9892E-01	-0,26
Q60695	RGL1	2,9892E-01	-0,26
P70313	NOS3	2,9892E-01	-0,26
P08775	RPB1	2,9898E-01	-0,76
P31001	DESM	2,9941E-01	0,00
Q8BJS4	SUN2	2,9967E-01	-0,43
P62082	RS7	3,0000E-01	0,15
O70252	HMOX2	3,0025E-01	-0,60
Q8R326	PSPC1	3,0034E-01	-0,45
Q3UIU2	NDUB6	3,0037E-01	0,51

P63276	RS17	3,0090E-01	0,16
P24288	BCAT1	3,0130E-01	-0,50
Q922L6	NELFD	3,0159E-01	-0,60
Q8BNE1	TCAF1	3,0172E-01	0,51
Q920Q8	NS1BP	3,0194E-01	-0,57
Q9WUL7	ARL3	3,0199E-01	-0,47
Q4KWH5	PLCH1	3,0288E-01	-0,53
Q8R3Y5	CS047	3,0288E-01	-0,53
P46938	YAP1	3,0288E-01	-0,53
Q9DBS1	TMM43	3,0302E-01	-0,47
Q8BGB7	ENOPH	3,0352E-01	-0,60
Q3UJB9	EDC4	3,0357E-01	-0,49
Q99L45	IF2B	3,0366E-01	-0,44
Q921G8	GCP2	3,0367E-01	-0,60
Q91WJ8	FUBP1	3,0478E-01	-0,37
Q9ET01	PYGL	3,0505E-01	-0,56
Q8CAK1	CAF17	3,0543E-01	0,32
P57784	RU2A	3,0567E-01	-0,56
P52480	KPYM	3,0571E-01	-0,05
Q01405	SC23A	3,0590E-01	0,03
Q9DC51	GNAI3	3,0616E-01	0,13
Q9D8C4	IN35	3,0618E-01	-0,60
Q80WW9	DDRGK	3,0618E-01	-0,60
Q9Z1M8	RED	3,0618E-01	-0,60
Q04857	CO6A1	3,0621E-01	0,00
Q9DB25	ALG5	3,0641E-01	0,32
P14211	CALR	3,0717E-01	-0,03
Q80W54	FACE1	3,0761E-01	0,22
Q99KK7	DPP3	3,0816E-01	0,04
Q8VE70	PDC10	3,0951E-01	0,32
Q8BVI4	DHPR	3,0977E-01	0,14
O88947	FA10	3,1008E-01	-0,53
P40240	CD9	3,1008E-01	-0,53
Q8BYN3	ITPK1	3,1008E-01	-0,53
Q9QXG4	ACSA	3,1008E-01	-0,53
Q2TBE6	P4K2A	3,1008E-01	-0,53
A2AWA9	RBGP1	3,1008E-01	-0,53
P37889	FBLN2	3,1008E-01	-0,53
P58269	DPF3	3,1008E-01	-0,53
P09813	APOA2	3,1008E-01	-0,53
Q8K409	DPOLB	3,1008E-01	-0,53
Q8K301	DDX52	3,1008E-01	-0,53
Q8C078	KKCC2	3,1008E-01	-0,53

P01642	KV5A9	3,1008E-01	-0,53
Q9QZV9	NXT1	3,1008E-01	-0,53
Q6P4S6	SIK3	3,1008E-01	-0,53
P60867	RS20	3,1017E-01	0,14
O54941	SMCE1	3,1067E-01	-0,48
P10630	IF4A2	3,1082E-01	0,07
Q3TJD7	PDLI7	3,1087E-01	-0,50
P14733	LMNB1	3,1105E-01	-0,05
P55258	RAB8A	3,1164E-01	-0,59
Q9Z2I9	SUCB1	3,1182E-01	0,05
Q9D7J4	COX20	3,1193E-01	0,32
P29387	GBB4	3,1252E-01	-0,59
P47740	AL3A2	3,1256E-01	0,11
P0DP26	CALM1	3,1301E-01	0,14
Q76LS9	MINY1	3,1432E-01	-0,54
Q9QXB9	DRG2	3,1463E-01	-0,52
Q8R3G9	TSN8	3,1485E-01	0,61
Q8K0E8	FIBB	3,1493E-01	-0,35
P16294	FA9	3,1504E-01	-0,58
P48025	KSYK	3,1504E-01	-0,58
Q8VI75	IPO4	3,1529E-01	-0,54
Q60631	GRB2	3,1566E-01	-0,52
P18525	HVM54	3,1580E-01	-0,31
Q9D832	DNJB4	3,1661E-01	0,17
Q8R307	VPS18	3,1685E-01	-0,58
Q3ULW8	PARP3	3,1685E-01	-0,58
Q91ZV0	MIA2	3,1689E-01	0,32
Q99K85	SERC	3,1696E-01	-0,54
Q9JIZ9	PLS3	3,1706E-01	0,26
Q8R2T8	TF3C5	3,1718E-01	-0,60
O08583	THOC4	3,1771E-01	-0,46
Q99LD4	CSN1	3,1782E-01	-0,46
O09164	SODE	3,1821E-01	0,32
P10711	TCEA1	3,1860E-01	0,12
Q61586	GPAT1	3,1883E-01	0,17
F6ZDS4	TPR	3,1885E-01	-0,44
P24369	PPIB	3,1935E-01	0,05
Q91YN5	UAP1	3,1963E-01	-0,58
P62245	RS15A	3,2030E-01	0,17
Q8K2X3	STN1	3,2044E-01	-0,58
Q8VCN9	TBCC	3,2076E-01	-0,57
P97377	CDK2	3,2101E-01	-0,33
P18872	GNAO	3,2128E-01	0,17

Q8CF89	TAB1	3,2146E-01	-0,58
Q8C8R3	ANK2	3,2158E-01	-0,57
Q80X82	SYMPK	3,2175E-01	-0,74
P62897	CYC	3,2191E-01	0,14
O35226	PSMD4	3,2252E-01	0,07
Q3UMC0	AFG2H	3,2328E-01	-0,57
P01863	GCAA	3,2355E-01	0,70
Q8VI36	PAXI	3,2362E-01	0,22
Q923D2	BLVRB	3,2441E-01	0,15
Q8R323	RFC3	3,2465E-01	0,32
Q8BHB4	WDR3	3,2590E-01	-0,58
O88470	FOXL2	3,2596E-01	-0,57
B8JK39	ITA9	3,2596E-01	-0,57
P48722	HS74L	3,2605E-01	-0,43
P49182	HEP2	3,2671E-01	-0,55
P52432	RPAC1	3,2694E-01	-0,50
P26618	PGFRA	3,2722E-01	-0,26
Q91VN6	DDX41	3,2732E-01	-0,55
Q9Z0E0	NCDN	3,2772E-01	-0,57
P33622	APOC3	3,2772E-01	-0,57
P48771	CX7A2	3,2774E-01	0,32
Q8BX09	RBBP5	3,2781E-01	-0,55
Q5XG73	ACBD5	3,2784E-01	0,32
P61957	SUMO2	3,2819E-01	0,34
A6H611	MIPEP	3,2830E-01	-0,57
O55131	SEPT7	3,2849E-01	0,04
Q9CXS4	CENPV	3,2871E-01	0,07
O35730	RING1	3,2873E-01	-0,57
Q9QXX4	CMC2	3,2877E-01	-0,43
Q8BH35	CO8B	3,2884E-01	-0,68
F8VPU2	FARP1	3,2887E-01	0,32
Q5SQX6	CYFP2	3,2920E-01	-0,57
Q9QZ73	DCNL1	3,2952E-01	0,32
P40336	VP26A	3,3001E-01	0,12
Q8BXV2	BRI3B	3,3014E-01	0,32
P58242	ASM3B	3,3082E-01	0,19
Q6PDG5	SMRC2	3,3128E-01	-0,49
P55284	CADH5	3,3129E-01	-0,57
Q9CPX6	ATG3	3,3135E-01	-0,53
P08905	LYZ2	3,3144E-01	-0,55
P83877	TXN4A	3,3144E-01	-0,55
Q91Z31	PTBP2	3,3144E-01	-0,55
Q9R0N0	GALK1	3,3167E-01	-0,38

Q78IK2	ATPMD	3,3168E-01	0,25
P58389	PTPA	3,3184E-01	-0,51
Q8VCS3	XYLK	3,3216E-01	0,51
P62320	SMD3	3,3235E-01	-0,47
O35295	PURB	3,3250E-01	0,09
P62911	RL32	3,3251E-01	0,32
Q08857	CD36	3,3301E-01	-0,57
Q8JZM7	CDC73	3,3301E-01	-0,57
Q61001	LAMA5	3,3307E-01	0,25
Q03734	SPA3M	3,3333E-01	-0,47
Q80WC7	AGFG2	3,3439E-01	-0,64
P18527	HVM56	3,3467E-01	0,46
P11680	PROP	3,3538E-01	-0,57
Q9CWW6	PIN4	3,3539E-01	-0,55
Q3TPX4	EXOC5	3,3539E-01	-0,55
Q8C1E7	T120A	3,3539E-01	-0,55
Q8BWW9	PKN2	3,3556E-01	-0,53
Q9Z0M6	CD97	3,3563E-01	-0,57
Q6ZQ88	KDM1A	3,3566E-01	-0,55
P70195	PSB7	3,3574E-01	0,15
Q91WQ3	SYYC	3,3580E-01	0,06
Q922H4	GMPPA	3,3608E-01	0,25
Q80WJ7	LYRIC	3,3679E-01	0,25
Q6NZJ6	IF4G1	3,3717E-01	0,08
O35887	CALU	3,3717E-01	0,04
Q8BP40	PPA6	3,3754E-01	0,25
Q8BH74	NU107	3,3787E-01	-0,46
Q62074	KPCI	3,3816E-01	-0,57
P16330	CN37	3,3827E-01	0,25
Q8C5N3	CWC22	3,3968E-01	-0,55
Q8BXK8	AGAP1	3,3968E-01	-0,55
Q64449	MRC2	3,3968E-01	-0,55
Q06335	APLP2	3,3968E-01	-0,55
Q5SVR0	TBC9B	3,3968E-01	-0,55
Q99M51	NCK1	3,3998E-01	0,32
Q8BHA3	DTD2	3,3998E-01	0,32
Q9QX47	SON	3,4098E-01	-0,57
Q99PT1	GDIR1	3,4103E-01	-0,35
Q9CQR2	RS21	3,4149E-01	0,11
E9Q735	UBE4A	3,4161E-01	0,32
P33242	STF1	3,4178E-01	-0,56
Q78IS1	TMED3	3,4185E-01	0,32
P97379	G3BP2	3,4198E-01	-0,49

Q8VCA8	SCRN2	3,4204E-01	-0,42
Q9QZ08	NAGK	3,4213E-01	-0,49
Q9QXE7	TBL1X	3,4227E-01	-0,43
Q8R5J9	PRAF3	3,4261E-01	0,25
Q9JIF7	COPB	3,4267E-01	-0,31
Q921F2	TADBP	3,4299E-01	-0,35
Q8BMD8	SCMC1	3,4330E-01	-0,44
Q3UE37	UBE2Z	3,4354E-01	-0,54
Q8K1N1	PLPL8	3,4371E-01	0,32
Q91VC9	GHITM	3,4371E-01	0,32
P00405	COX2	3,4376E-01	0,12
P61082	UBC12	3,4402E-01	0,18
Q6P9R2	OXSR1	3,4482E-01	-0,50
Q9QYR6	MAP1A	3,4484E-01	-0,48
Q11136	PEPD	3,4506E-01	-0,39
P42567	EPS15	3,4541E-01	0,21
P39749	FEN1	3,4562E-01	-0,54
Q9EST5	AN32B	3,4581E-01	0,10
P42232	STA5B	3,4606E-01	0,15
Q9CZW4	ACSL3	3,4615E-01	-0,26
Q9DC29	ABCB6	3,4621E-01	-0,51
Q9QYA2	TOM40	3,4644E-01	0,13
Q9CQI3	GMFB	3,4664E-01	0,16
O54946	DNJB6	3,4686E-01	0,31
O08807	PRDX4	3,4697E-01	-0,40
P70335	ROCK1	3,4704E-01	0,28
Q9WUM5	SUCA	3,4711E-01	0,14
Q61655	DD19A	3,4775E-01	-0,42
Q9CQ48	NUDC2	3,4790E-01	-0,54
Q3B7Z2	OSBP1	3,4837E-01	0,14
O70475	UGDH	3,4858E-01	-0,35
O54974	LEG7	3,4860E-01	-0,49
Q7TSQ8	PDPR	3,4862E-01	-0,55
O70311	NMT2	3,4863E-01	-0,52
Q9EQP2	EHD4	3,4868E-01	0,06
O54781	SRPK2	3,4869E-01	-0,31
P08121	CO3A1	3,4900E-01	-0,53
B2RSH2	GNAI1	3,4906E-01	0,26
Q64433	CH10	3,4939E-01	0,10
P62774	MTPN	3,5072E-01	0,25
Q9ERA6	TFP11	3,5110E-01	-0,58
Q91V81	RBM42	3,5115E-01	-0,54
Q99J99	THTM	3,5169E-01	-0,43

Q4VBE8	WDR18	3,5172E-01	-0,50
Q8VC70	RBMS2	3,5186E-01	0,32
Q8C570	RAE1L	3,5235E-01	-0,43
Q60960	IMA5	3,5266E-01	0,28
Q9ET26	RN114	3,5320E-01	-0,51
Q9DBR0	AKAP8	3,5323E-01	-0,54
Q8BMS4	COQ3	3,5389E-01	0,32
P01901	HA1B	3,5394E-01	0,13
Q99LC3	NDUAA	3,5396E-01	0,10
Q9D0C1	RN115	3,5421E-01	0,32
Q9EQQ2	YIPF5	3,5421E-01	0,32
Q8K009	AL1L2	3,5490E-01	0,35
P12382	PFKAL	3,5571E-01	-0,38
Q9CWP6	MSPD2	3,5625E-01	0,32
Q8VDG5	PPCS	3,5646E-01	0,32
Q01730	RSU1	3,5724E-01	-0,51
Q61937	NPM	3,5725E-01	-0,35
Q9R257	HEBP1	3,5730E-01	-0,54
Q9R0U0	SRS10	3,5750E-01	0,18
P53810	PIPNA	3,5786E-01	0,12
P08003	PDIA4	3,5794E-01	-0,04
Q9D358	PPAC	3,5798E-01	0,18
Q922B1	MACD1	3,5813E-01	-0,54
Q9JHJ0	TMOD3	3,5886E-01	0,16
P14576	SRP54	3,5923E-01	0,14
P56391	CX6B1	3,5939E-01	0,18
D0QMC3	MNDAL	3,5940E-01	-0,27
A3KMP2	TTC38	3,5991E-01	0,10
Q9QYI3	DNJC7	3,6001E-01	-0,42
P35585	AP1M1	3,6007E-01	-0,47
E9Q555	RN213	3,6016E-01	-0,55
Q60692	PSB6	3,6063E-01	-0,47
Q99L47	F10A1	3,6138E-01	0,07
O89112	LANC1	3,6161E-01	-0,54
Q9DBL7	COASY	3,6179E-01	0,21
Q5SRX1	TM1L2	3,6257E-01	-0,51
P51557	STAR	3,6287E-01	0,12
P70188	KIFA3	3,6294E-01	0,32
Q9JI39	ABCBA	3,6294E-01	0,32
Q9R0E1	PLOD3	3,6306E-01	-0,47
P59999	ARPC4	3,6354E-01	0,21
Q9CZ13	QCR1	3,6406E-01	-0,02
Q07076	ANXA7	3,6425E-01	0,05

Q8BTZ7	GMPPB	3,6462E-01	0,21
Q9CYR6	AGM1	3,6491E-01	0,16
Q62448	IF4G2	3,6552E-01	-0,43
Q8BU33	ILVBL	3,6586E-01	0,32
O54833	CSK22	3,6625E-01	-0,45
O88393	TGBR3	3,6635E-01	-0,53
Q8VHI3	OFUT2	3,6727E-01	-0,54
Q9EPC1	PARVA	3,6769E-01	-0,40
Q60598	SRC8	3,6776E-01	-0,37
O09174	AMACR	3,6781E-01	0,32
P58404	STRN4	3,6889E-01	-0,53
Q9JK53	PRELP	3,6898E-01	-0,45
Q9DBX6	CP2S1	3,6971E-01	-0,53
Q9CXG3	PPIL4	3,6971E-01	-0,53
P30416	FKBP4	3,6992E-01	-0,02
Q3U0V2	TRADD	3,6997E-01	0,21
Q9DBF1	AL7A1	3,7006E-01	-0,36
P43274	H14	3,7050E-01	0,12
Q3UMB9	WASC4	3,7082E-01	-0,68
Q811U4	MFN1	3,7113E-01	-0,53
Q9WVA4	TAGL2	3,7125E-01	-0,33
Q8CFX1	G6PE	3,7128E-01	-0,40
P06909	CFAH	3,7157E-01	-1,10
Q64511	TOP2B	3,7203E-01	-0,54
Q63918	CAVN2	3,7223E-01	0,18
Q99JY3	GIMA4	3,7275E-01	0,13
Q9D666	SUN1	3,7318E-01	-0,50
O35465	FKBP8	3,7326E-01	0,12
O55022	PGRC1	3,7342E-01	0,14
Q8BVY0	RL1D1	3,7347E-01	-0,53
Q99JY9	ARP3	3,7382E-01	0,01
P11983	TCPA	3,7386E-01	-0,28
Q9WTX5	SKP1	3,7442E-01	-0,49
O09159	MA2B1	3,7450E-01	-0,47
Q9CWJ9	PUR9	3,7490E-01	-0,31
Q9DAU1	CNPY3	3,7633E-01	0,32
O08599	STXB1	3,7642E-01	0,25
Q9QX60	DGUOK	3,7649E-01	0,25
Q60870	REEP5	3,7652E-01	0,32
O88587	COMT	3,7670E-01	-0,53
P70336	ROCK2	3,7687E-01	-1,10
Q9EPB5	SERHL	3,7700E-01	0,32
Q9JLV1	BAG3	3,7806E-01	0,20

P61255	RL26	3,7836E-01	0,24
Q63836	SBP2	3,7848E-01	0,17
O08749	DLDH	3,7870E-01	0,00
Q9R0Q3	TMED2	3,7876E-01	0,20
O35114	SCRB2	3,7922E-01	-0,49
Q8CES0	NAA30	3,7986E-01	-0,50
D3YZP9	CCDC6	3,8047E-01	0,24
Q9DAR7	DCPS	3,8048E-01	-0,37
Q8BJW6	EIF2A	3,8133E-01	0,13
P01872	IGHM	3,8150E-01	-0,50
Q99KF1	TMED9	3,8157E-01	0,14
Q8JZX4	SPF45	3,8165E-01	-0,56
Q9CWE0	MFR1L	3,8180E-01	0,20
Q149G0	CB068	3,8224E-01	0,32
Q9DAW9	CNN3	3,8249E-01	-0,33
Q9QZI8	SERC1	3,8364E-01	0,32
Q5EBG8	CA050	3,8364E-01	0,32
Q78YY6	DJC15	3,8364E-01	0,32
Q99LP8	YIPF2	3,8364E-01	0,32
Q8BYK8	ZC3H6	3,8364E-01	0,32
Q8VD76	TF2H3	3,8364E-01	0,32
Q9CQ56	USE1	3,8364E-01	0,32
P70280	VAMP7	3,8364E-01	0,32
Q9JK38	GNA1	3,8364E-01	0,32
Q9DCC8	TOM20	3,8364E-01	0,32
Q9D8Y1	T126A	3,8364E-01	0,32
Q8BRH0	TMTC3	3,8364E-01	0,32
Q9JJT0	RCL1	3,8364E-01	0,32
Q4VAE3	TMM65	3,8364E-01	0,32
Q8K2A1	GULP1	3,8364E-01	0,32
Q9DBY1	SYVN1	3,8364E-01	0,32
Q9R1Z7	PTPS	3,8364E-01	0,32
Q61624	ZN148	3,8364E-01	0,32
Q8R1N0	ZN830	3,8364E-01	0,32
P58059	RT21	3,8364E-01	0,32
Q9D3B1	HACD2	3,8364E-01	0,32
Q8R5F7	IFIH1	3,8364E-01	0,32
Q9JM13	RABX5	3,8364E-01	0,32
Q8BJZ4	RT35	3,8364E-01	0,32
Q91WL8	WWOX	3,8364E-01	0,32
Q9R061	NUBP2	3,8364E-01	0,32
Q9CWD8	NUBPL	3,8364E-01	0,32
Q91WV0	NC2B	3,8364E-01	0,32

P08074	CBR2	3,8364E-01	0,32
Q8C8T8	TSR2	3,8364E-01	0,32
Q9Z210	PX11B	3,8386E-01	0,20
P51150	RAB7A	3,8421E-01	0,02
Q8K2B0	SC65	3,8539E-01	0,32
Q8BH04	PCKGM	3,8668E-01	-0,35
Q9CW46	RAVR1	3,8705E-01	-0,46
P62267	RS23	3,8716E-01	0,20
Q9CXW3	CYBP	3,8724E-01	-0,43
Q9D2N9	VP33A	3,8735E-01	0,32
Q9WUD8	FAIM1	3,8735E-01	0,32
Q9CXW2	RT22	3,8735E-01	0,32
O70591	PFD2	3,8766E-01	-0,51
Q9Z0P4	PALM	3,8790E-01	0,12
Q99LB6	MAT2B	3,8806E-01	-0,43
P10922	H10	3,8832E-01	0,13
Q9CPY7	AMPL	3,8889E-01	-0,02
O88712	CTBP1	3,8923E-01	0,08
Q99LU0	CH1B1	3,8930E-01	0,32
P11930	NUD19	3,8983E-01	0,32
Q99J77	SIAS	3,9110E-01	-0,43
Q91YH5	ATLA3	3,9116E-01	0,02
Q8BU85	MSRB3	3,9136E-01	-0,68
Q64442	DHSO	3,9230E-01	-0,51
O35343	IMA3	3,9252E-01	0,15
Q61687	ATRX	3,9263E-01	-0,90
O08788	DCTN1	3,9293E-01	-0,42
Q62241	RU1C	3,9378E-01	-0,51
Q9CPP6	NDUA5	3,9383E-01	0,24
Q9JMA1	UBP14	3,9384E-01	-0,38
P06537	GCR	3,9410E-01	0,32
Q62084	PP14B	3,9458E-01	-0,51
Q3TAS6	EMC10	3,9489E-01	0,32
P02802	MT1	3,9489E-01	0,32
Q8VEK0	CC50A	3,9489E-01	0,32
Q571I9	A16A1	3,9626E-01	-0,46
Q9CQF3	CPSF5	3,9627E-01	-0,46
Q9CZM2	RL15	3,9664E-01	0,20
P20029	BIP	3,9675E-01	-0,09
Q9DCZ1	GMPR1	3,9710E-01	-0,51
P67984	RL22	3,9724E-01	0,10
Q8VDK1	NIT1	3,9743E-01	-0,46
Q8R349	CDC16	3,9846E-01	-0,43

Q9D7A6	SRP19	3,9886E-01	0,32
P45591	COF2	3,9898E-01	0,16
P97868	RBBP6	3,9905E-01	0,32
Q5SWD9	TSR1	3,9911E-01	-0,51
Q9CPP0	NPM3	3,9960E-01	-0,51
Q9QZZ6	DERM	3,9978E-01	0,32
Q3UIA2	RHG17	4,0030E-01	0,32
Q9QZD9	EIF3I	4,0073E-01	-0,37
P49312	ROA1	4,0078E-01	-0,30
Q924D0	RT4I1	4,0123E-01	0,32
Q99KR7	PPIF	4,0123E-01	0,32
P25976	UBF1	4,0174E-01	0,20
Q8R5H1	UBP15	4,0215E-01	-0,50
Q8QZY9	SF3B4	4,0230E-01	0,24
E9PV24	FIBA	4,0259E-01	-0,36
Q9R099	TBL2	4,0267E-01	0,16
Q8VCT3	AMPB	4,0285E-01	-0,37
P03899	NU3M	4,0307E-01	0,32
Q91ZR1	RAB4B	4,0332E-01	0,31
Q8BPM2	M4K5	4,0348E-01	0,06
P56394	COX17	4,0378E-01	0,32
O08734	BAK	4,0378E-01	0,32
Q80ZJ1	RAP2A	4,0405E-01	0,32
Q3V1T4	P3H1	4,0423E-01	-0,40
Q9DD03	RAB13	4,0430E-01	0,33
O70566	DIAP2	4,0436E-01	0,00
Q8BML9	SYQ	4,0462E-01	0,02
A2A8L1	CHD5	4,0531E-01	-0,43
Q9DB05	SNAA	4,0560E-01	-0,36
Q9CW03	SMC3	4,0595E-01	-0,36
Q8K3J1	NDUS8	4,0608E-01	0,24
C0HKD8	MFA1A	4,0647E-01	-0,49
Q3TC93	H1BP3	4,0689E-01	-0,68
Q8BKS9	PUM3	4,0712E-01	-0,46
Q8CIF4	BTD	4,0725E-01	0,23
P01869	IGH1M	4,0736E-01	-0,89
Q6PHQ8	NAA35	4,0754E-01	0,32
Q8R1S0	COQ6	4,0754E-01	0,32
Q9EP89	LACTB	4,0754E-01	0,32
Q9DC23	DJC10	4,0758E-01	-0,49
O88531	PPT1	4,0759E-01	-0,47
Q99J93	IFM2	4,0770E-01	0,28
Q01149	CO1A2	4,0773E-01	0,14

P97927	LAMA4	4,0775E-01	-0,62
P09925	SURF1	4,0803E-01	0,32
Q7TQI3	OTUB1	4,0812E-01	-0,38
Q6P2K6	P4R3A	4,0842E-01	-0,47
Q99JP6	HOME3	4,0881E-01	0,32
Q61475	DAF1	4,0881E-01	0,32
Q9DBL9	ABHD5	4,0881E-01	0,32
Q91Z49	UIF	4,0881E-01	0,32
Q8CHW4	EI2BE	4,0881E-01	0,32
Q8BG07	PLD4	4,0897E-01	0,32
Q810U5	CCD50	4,0897E-01	0,32
Q99KK9	SYHM	4,0902E-01	0,28
Q810U4	NRCAM	4,0949E-01	0,46
Q9DC16	ERGI1	4,0964E-01	-0,49
P52912	TIA1	4,0980E-01	0,28
Q9Z0Z4	HEPH	4,1006E-01	-0,43
P61327	MGN	4,1032E-01	-0,44
Q8VC03	EMAL3	4,1079E-01	0,32
Q8BTU1	CFA20	4,1079E-01	0,32
Q8BXA5	CLP1L	4,1079E-01	0,32
Q9QYY8	SPAST	4,1079E-01	0,32
Q9CX30	YIF1B	4,1079E-01	0,32
Q66GT5	PTPM1	4,1079E-01	0,32
P14685	PSMD3	4,1095E-01	0,01
Q3U9G9	LBR	4,1096E-01	-0,47
Q8BGA9	OXA1L	4,1150E-01	0,32
Q8BSF4	PISD	4,1150E-01	0,32
Q922E4	PCY2	4,1150E-01	0,32
Q9D0E1	HNRPM	4,1176E-01	-0,06
Q8K4B0	MTA1	4,1194E-01	-0,40
Q9JI10	STK3	4,1239E-01	-0,43
P00329	ADH1	4,1320E-01	-0,31
P51807	DYLT1	4,1438E-01	0,19
Q9D1M4	MCA3	4,1441E-01	0,32
O35711	LIPB2	4,1505E-01	-0,26
P61022	CHP1	4,1514E-01	0,19
B2RY56	RBM25	4,1521E-01	-0,43
Q9WV92	E41L3	4,1529E-01	0,21
Q9Z0J0	NPC2	4,1544E-01	0,19
Q9CQJ6	DENR	4,1545E-01	0,23
Q99LF4	RTCB	4,1549E-01	0,02
P21279	GNAQ	4,1587E-01	-0,42
P35279	RAB6A	4,1606E-01	0,07

Q61576	FKB10	4,1624E-01	-0,39
Q9D7N3	RT09	4,1677E-01	0,19
Q8BHG2	CZIB	4,1690E-01	0,23
Q9CZR8	EFTS	4,1691E-01	0,19
P19536	COX5B	4,1694E-01	0,32
Q569Z6	TR150	4,1697E-01	-0,44
P68510	1433F	4,1704E-01	0,02
Q8CI32	BAG5	4,1749E-01	-0,49
Q3U0V1	FUBP2	4,1763E-01	-0,31
Q9QUJ7	ACSL4	4,1764E-01	-0,38
Q8CH25	SLTM	4,1773E-01	-0,49
P61164	ACTZ	4,1796E-01	-0,37
Q9Z2W1	STK25	4,1835E-01	-0,31
P32067	LA	4,1859E-01	-0,36
P30677	GNA14	4,1883E-01	-0,25
Q9CQI7	RU2B	4,1936E-01	0,32
O35083	PLCA	4,1936E-01	0,32
P04627	ARAF	4,1936E-01	0,32
Q9D3P8	PLRKT	4,1936E-01	0,32
Q9CX00	IST1	4,1967E-01	0,23
Q3UDP0	WDR41	4,1980E-01	0,32
Q921H9	COA7	4,1990E-01	0,32
P49710	HCLS1	4,2006E-01	-0,49
P35285	RB22A	4,2021E-01	-0,42
P70122	SBDS	4,2113E-01	0,23
P97315	CSRP1	4,2157E-01	-0,32
Q9CXW4	RL11	4,2214E-01	0,12
P35123	UBP4	4,2222E-01	-0,54
Q2HXL6	EDEM3	4,2259E-01	-0,49
Q8BGT5	ALAT2	4,2308E-01	0,45
Q3UFY8	TM10C	4,2314E-01	0,32
Q8BGR9	UBCP1	4,2314E-01	0,32
Q8BR07	BICD1	4,2314E-01	0,32
Q6PCP5	MFF	4,2314E-01	0,32
P34152	FAK1	4,2314E-01	0,32
Q9D7M1	GID8	4,2314E-01	0,32
O55013	TPPC3	4,2327E-01	-0,49
Q91XF0	PNPO	4,2327E-01	-0,49
P56873	ZNRD2	4,2327E-01	-0,49
P45377	ALD2	4,2360E-01	-0,34
P47962	RL5	4,2414E-01	0,00
Q8K4F5	ABHDB	4,2434E-01	0,32
Q91YE8	SYNP2	4,2469E-01	-1,06

Q3UJD6	UBP19	4,2491E-01	0,16
P41317	MBL2	4,2562E-01	0,16
Q9Z2A9	GGT5	4,2584E-01	0,14
Q9D0K2	SCOT1	4,2632E-01	0,00
Q01147	CREB1	4,2651E-01	-0,49
O89013	OBRG	4,2651E-01	-0,49
P08122	CO4A2	4,2677E-01	-0,45
P43883	PLIN2	4,2724E-01	0,04
P97760	RPB3	4,2738E-01	-0,45
P07356	ANXA2	4,2754E-01	-0,05
P61079	UB2D3	4,2807E-01	-0,45
Q3UFY7	5NT3B	4,2858E-01	-0,40
Q9WU84	CCS	4,2902E-01	-0,49
P08752	GNAI2	4,3003E-01	0,01
Q8C181	MBNL2	4,3003E-01	-0,47
Q60770	STXB3	4,3038E-01	0,13
Q9JJU8	SH3L1	4,3041E-01	-0,42
Q8BZ98	DYN3	4,3114E-01	0,29
Q9WU78	PDC6I	4,3242E-01	0,00
Q922D8	C1TC	4,3278E-01	0,00
Q05512	MARK2	4,3359E-01	-0,48
Q9JM14	NT5C	4,3379E-01	-0,43
Q9JKB3	YBOX3	4,3413E-01	-0,36
Q9DBG9	TX1B3	4,3421E-01	-0,49
Q9EPU0	RENT1	4,3435E-01	-0,31
Q9CQT1	MTNA	4,3565E-01	-0,40
Q9WTU0	PHF2	4,3572E-01	0,32
P27546	MAP4	4,3672E-01	0,08
Q5EG47	AAPK1	4,3681E-01	0,18
Q80U87	UBP8	4,3720E-01	-0,53
Q60634	FLOT2	4,3776E-01	-0,38
P62908	RS3	4,3797E-01	-0,01
Q99L13	3HIDH	4,3840E-01	0,06
Q91Z38	TTC1	4,3923E-01	-0,47
P80314	TCPB	4,3979E-01	-0,06
Q8CDG3	VCIP1	4,3985E-01	-0,53
Q9WTP7	KAD3	4,4009E-01	0,04
Q80W22	THNS2	4,4070E-01	-0,47
Q9DCT1	AKCL2	4,4078E-01	-0,46
Q2TPA8	HSDL2	4,4121E-01	0,15
P19157	GSTP1	4,4134E-01	0,01
Q91VU7	PUS7	4,4175E-01	-0,46
P14115	RL27A	4,4187E-01	0,15

P26645	MARCS	4,4230E-01	-0,36
O35551	RABE1	4,4266E-01	0,74
Q6ZQL4	WDR43	4,4340E-01	-0,46
P20239	ZP2	4,4426E-01	-0,36
Q8BWW4	LARP4	4,4439E-01	-0,49
Q99NB8	UBQL4	4,4523E-01	-0,69
Q3TKT4	SMCA4	4,4552E-01	-0,82
P62137	PP1A	4,4561E-01	0,05
O88492	PLIN4	4,4574E-01	-0,82
O09012	PEX5	4,4620E-01	0,23
Q62465	VAT1	4,4623E-01	-0,35
Q3THG9	AASD1	4,4719E-01	-0,39
Q8K124	PKHO2	4,4724E-01	-0,47
Q9D0B0	SRSF9	4,4754E-01	-0,42
P21550	ENOB	4,4771E-01	0,10
Q99MD9	NASP	4,4799E-01	-0,39
Q9JLI8	SART3	4,4833E-01	-0,36
P11531	DMD	4,4995E-01	-0,47
P54869	HMCS2	4,5021E-01	-0,04
Q9D0M5	DYL2	4,5031E-01	0,16
Q69ZQ2	ISY1	4,5063E-01	0,22
Q8K182	CO8A	4,5069E-01	-0,68
Q9CU62	SMC1A	4,5077E-01	-0,49
Q8BMF4	ODP2	4,5138E-01	0,01
O55060	TPMT	4,5145E-01	0,18
Q8BP47	SYNC	4,5148E-01	-0,31
Q9D8Y0	EFHD2	4,5171E-01	-0,39
Q9R013	CATF	4,5220E-01	0,18
Q9D8S4	ORN	4,5234E-01	-0,46
Q8R0F3	SUMF1	4,5234E-01	-0,46
P15089	CBPA3	4,5263E-01	0,13
P97863	NFIB	4,5279E-01	0,29
Q9JK23	PSMG1	4,5301E-01	-0,43
Q9JKP5	MBNL1	4,5307E-01	-0,46
Q9DCD2	SYF1	4,5312E-01	-0,40
Q9CXZ1	NDUS4	4,5456E-01	0,22
P56395	CYB5	4,5502E-01	-0,37
P62307	RUXF	4,5505E-01	0,18
Q8CJ40	CROCC	4,5539E-01	-0,42
P47753	CAZA1	4,5553E-01	0,04
Q91VH2	SNX9	4,5590E-01	-0,43
Q8C522	ENDD1	4,5592E-01	0,11
Q8BYB9	PGLT1	4,5607E-01	-0,46

Q8C5W3	TBCEL	4,5627E-01	0,15
P46412	GPX3	4,5706E-01	0,15
P09405	NUCL	4,5731E-01	-0,26
P35486	ODPA	4,5774E-01	0,02
Q9WU40	MAN1	4,5844E-01	-0,53
P49935	CATH	4,5910E-01	0,18
P42703	LIFR	4,6005E-01	-0,46
Q3THS6	METK2	4,6031E-01	-0,36
Q8C5Q4	GRSF1	4,6046E-01	0,22
Q9CR98	F136A	4,6178E-01	-0,46
Q91V09	WDR13	4,6179E-01	-0,42
P56183	RRP1	4,6179E-01	-0,42
Q8BZA9	TIGAR	4,6179E-01	-0,42
Q91YR1	TWF1	4,6230E-01	0,06
P35282	RAB21	4,6235E-01	0,08
Q9D1J3	SARNP	4,6247E-01	0,11
Q8VD66	ABHD4	4,6265E-01	0,18
Q8CHP8	PGP	4,6266E-01	0,08
P52503	NDUS6	4,6293E-01	0,15
Q9JIH2	NUP50	4,6325E-01	-0,68
O70572	NSMA	4,6367E-01	0,15
Q9R1C7	PR40A	4,6378E-01	-0,62
Q9Z2U0	PSA7	4,6406E-01	0,01
Q80XR2	AT2C1	4,6408E-01	-0,46
Q9WV80	SNX1	4,6447E-01	-0,32
O54984	ASNA	4,6513E-01	0,06
P42227	STAT3	4,6565E-01	-0,33
P51855	GSHB	4,6644E-01	-0,42
P04104	K2C1	4,6649E-01	0,28
O35098	DPYL4	4,6663E-01	-0,44
Q9WUB3	PYGM	4,6710E-01	-0,37
Q8VDP4	CCAR2	4,6711E-01	-0,34
Q9ERG2	STRN3	4,6744E-01	-0,43
P26041	MOES	4,6750E-01	-0,26
P70261	PALD	4,6786E-01	-0,44
Q9CPQ8	ATP5L	4,6795E-01	-0,44
Q91WT8	RBM47	4,6831E-01	0,09
Q8CG03	PDE5A	4,6836E-01	-0,42
P10493	NID1	4,6870E-01	0,00
Q91YT7	YTHD2	4,6872E-01	-0,45
Q9CRA4	MSMO1	4,6907E-01	0,22
Q9DB77	QCR2	4,6927E-01	-0,04
Q62433	NDRG1	4,6931E-01	-0,42

O09167	RL21	4,6950E-01	-0,44
Q9DBP5	KCY	4,7032E-01	0,09
Q8CBY8	DCTN4	4,7060E-01	-0,44
Q8BKU8	TM87B	4,7100E-01	-0,53
Q91V61	SFXN3	4,7126E-01	0,02
Q9JK48	SHLB1	4,7143E-01	-0,44
P56375	ACYP2	4,7158E-01	-0,42
Q3U7U3	FBX7	4,7158E-01	-0,42
B8ZXI1	QTRT2	4,7158E-01	-0,42
Q3V0K9	PLSI	4,7246E-01	-0,43
Q19LI2	A1BG	4,7257E-01	-0,29
Q810A7	DDX42	4,7276E-01	-0,39
Q9CPV4	GLOD4	4,7369E-01	-0,31
Q64487	PTPRD	4,7372E-01	-0,68
Q62165	DAG1	4,7440E-01	-0,42
Q61592	GAS6	4,7482E-01	0,24
Q9ER69	FL2D	4,7488E-01	-0,44
O54901	OX2G	4,7508E-01	-0,44
Q9R233	TPSN	4,7541E-01	0,17
Q9Z0L8	GGH	4,7570E-01	0,17
Q8BHJ5	TBL1R	4,7606E-01	0,15
P60766	CDC42	4,7621E-01	0,06
Q8K3H0	DP13A	4,7631E-01	-0,37
Q9DB20	ATPO	4,7644E-01	0,09
Q9WVB0	RBPMS	4,7682E-01	-0,44
Q99PU8	DHX30	4,7698E-01	-0,49
Q9D883	U2AF1	4,7705E-01	0,05
Q8VBT0	TMX1	4,7756E-01	0,17
P97390	VPS45	4,7801E-01	0,17
P49769	PSN1	4,7806E-01	0,22
Q8BUY9	PGTB1	4,7814E-01	-0,42
Q8BZX4	SREK1	4,7814E-01	-0,42
Q8C754	VPS52	4,7814E-01	-0,42
P21958	TAP1	4,7814E-01	-0,42
Q922U1	PRPF3	4,7814E-01	-0,42
Q9CQG2	MET16	4,7814E-01	-0,42
Q8CD10	MICU2	4,7814E-01	-0,42
Q3U308	CTU2	4,7814E-01	-0,42
Q8BU03	PWP2	4,7814E-01	-0,42
A2AR02	PPIG	4,7814E-01	-0,42
P70245	EBP	4,7815E-01	0,22
P48410	ABCD1	4,7815E-01	0,22
Q925E7	2ABD	4,7832E-01	-0,26

O08709	PRDX6	4,7842E-01	-0,04
Q8JZN5	ACAD9	4,7846E-01	0,00
Q6Y7W8	GGYF2	4,7891E-01	-0,33
Q8BMF3	MAON	4,7915E-01	-0,52
Q922V4	PLRG1	4,7918E-01	-0,40
Q3UJU9	RMD3	4,7988E-01	0,12
Q9DBN5	LONP2	4,8016E-01	0,17
Q922J9	FACR1	4,8035E-01	0,04
Q6Y685	TACC1	4,8041E-01	0,22
Q9CQB5	CISD2	4,8041E-01	0,22
P05064	ALDOA	4,8080E-01	-0,07
Q8R1X6	SPART	4,8107E-01	0,32
Q9Z130	HNRDL	4,8124E-01	-0,36
O70439	STX7	4,8134E-01	0,12
P35846	FOLR1	4,8179E-01	0,14
P14131	RS16	4,8191E-01	0,07
Q8BX17	GEMI5	4,8194E-01	-0,68
Q8CIM8	INT4	4,8194E-01	-0,68
Q6R0H7	GNAS1	4,8230E-01	-0,38
Q8BKT7	THOC5	4,8237E-01	0,17
P42669	PURA	4,8245E-01	0,04
D3YZV8	CCDC8	4,8250E-01	-0,68
Q9ES28	ARHG7	4,8266E-01	0,10
Q80UW8	RPAB1	4,8275E-01	0,22
Q9Z0N1	IF2G	4,8298E-01	-0,02
P26883	FKB1A	4,8299E-01	0,07
P09055	ITB1	4,8343E-01	-0,31
Q62418	DBNL	4,8422E-01	0,02
Q3U5Q7	CMPK2	4,8437E-01	0,07
P48024	EIF1	4,8460E-01	0,17
Q9DBB5	IF4E3	4,8486E-01	-0,42
Q9DCS1	T176A	4,8486E-01	-0,42
P54761	EPHB4	4,8486E-01	-0,42
P62254	UB2G1	4,8486E-01	-0,42
Q64669	NQO1	4,8486E-01	-0,42
Q8R502	LRC8C	4,8486E-01	-0,42
Q8R059	GALE	4,8490E-01	-0,36
Q9JI44	DMAP1	4,8490E-01	-0,36
Q9CWK3	CD2B2	4,8490E-01	-0,36
P55821	STMN2	4,8490E-01	-0,36
Q8BIF0	C99L2	4,8490E-01	-0,36
Q8BZT9	LACC1	4,8490E-01	-0,36
Q62191	RO52	4,8490E-01	-0,36

Q9ERA0	TFCP2	4,8490E-01	-0,36
Q9CQ89	CUTA	4,8490E-01	-0,36
Q8CBY3	LENG8	4,8490E-01	-0,36
Q8C1D8	IWS1	4,8490E-01	-0,36
Q9R1Q7	PLP2	4,8490E-01	-0,36
P59114	CAPAM	4,8490E-01	-0,36
Q6P5C5	SMUG1	4,8490E-01	-0,36
Q8CC86	PNCB	4,8490E-01	-0,36
O54782	MA2B2	4,8490E-01	-0,36
Q9D2N4	DTNA	4,8490E-01	-0,36
Q9QXA1	CYHR1	4,8490E-01	-0,36
P01637	KV5A5	4,8490E-01	-0,36
Q9CQJ2	PIHD1	4,8490E-01	-0,36
Q61035	SYHC	4,8508E-01	-0,33
P70290	EM55	4,8509E-01	-0,42
O08573	LEG9	4,8522E-01	-0,40
Q8BHD0	RB39A	4,8525E-01	-0,25
P70296	PEBP1	4,8531E-01	-0,03
P02089	HBB2	4,8577E-01	0,15
Q99MQ4	ASPN	4,8643E-01	0,14
Q9JJF3	RIOX1	4,8646E-01	-0,68
Q78PY7	SND1	4,8708E-01	-0,06
Q9CZJ2	HS12B	4,8776E-01	-0,36
P49443	PPM1A	4,8779E-01	0,09
Q9D819	IPYR	4,8782E-01	-0,32
Q64522	H2A2B	4,8790E-01	-0,27
P82198	BGH3	4,8851E-01	-0,29
Q8BL97	SRSF7	4,8883E-01	-0,36
Q9D8B4	NDUAB	4,8903E-01	-0,42
Q9R1T2	SAE1	4,8925E-01	-0,33
P28660	NCKP1	4,8938E-01	0,03
P60060	SC61G	4,8974E-01	0,22
Q9D7H3	RTCA	4,9050E-01	-0,44
Q8R2U4	NTM1A	4,9056E-01	-0,44
Q6PIP5	NUDC1	4,9091E-01	-0,39
Q9CZR2	NALD2	4,9111E-01	-0,34
P97386	DNLI3	4,9124E-01	-0,42
O88986	KBL	4,9124E-01	-0,42
Q9CQH7	BT3L4	4,9124E-01	-0,42
Q8BIJ7	RUFY1	4,9124E-01	-0,42
Q9Z0F8	ADA17	4,9134E-01	-0,04
G5E897	PLGT3	4,9144E-01	-0,42
P06797	CATL1	4,9146E-01	0,12

Q9CVB6	ARPC2	4,9188E-01	0,03
Q8BWM0	PGES2	4,9222E-01	-0,38
Q91VU0	FAM3C	4,9235E-01	0,22
Q9CQW2	ARL8B	4,9243E-01	0,13
P62488	RPB7	4,9251E-01	-0,55
Q9CPT4	MYDGF	4,9290E-01	-0,42
Q505F5	LRC47	4,9299E-01	-0,34
Q9CQV8	1433B	4,9328E-01	-0,03
Q60714	S27A1	4,9330E-01	0,09
Q8VDN2	AT1A1	4,9437E-01	-0,08
P58252	EF2	4,9498E-01	-0,23
Q8BMJ2	SYLC	4,9548E-01	-0,33
P36536	SAR1A	4,9566E-01	-0,40
P46467	VPS4B	4,9777E-01	-0,42
Q62261	SPTB2	4,9787E-01	-0,10
Q8BIW1	PRUN1	4,9812E-01	-0,40
Q922Q4	P5CR2	4,9823E-01	0,07
Q9D8U8	SNX5	4,9881E-01	0,05
Q9CRC9	GNPI2	4,9934E-01	0,16
P19426	NELFE	4,9935E-01	-0,36
Q3UM29	COG7	4,9935E-01	-0,36
Q9JIS8	S12A4	4,9935E-01	-0,36
Q7TSS2	UB2Q1	4,9935E-01	-0,36
Q9CVD2	ATX3	4,9935E-01	-0,36
P97287	MCL1	4,9935E-01	-0,36
Q9D0C4	TRM5	4,9935E-01	-0,36
Q9CWN7	CNO11	4,9935E-01	-0,36
Q6NXI6	RPRD2	4,9935E-01	-0,36
E9PYH6	SET1A	4,9935E-01	-0,36
Q8CJF7	ELYS	4,9935E-01	-0,36
P03921	NU5M	4,9935E-01	-0,36
P22437	PGH1	4,9935E-01	-0,36
Q80V86	INT8	4,9935E-01	-0,36
Q91WG8	GLCNE	4,9935E-01	-0,36
A2RTL5	RSRC2	4,9935E-01	-0,36
Q8CHQ0	FBX4	4,9936E-01	-0,36
Q69ZR2	HECD1	4,9942E-01	0,32
P56528	CD38	4,9942E-01	0,32
Q9CY57	CHTOP	4,9962E-01	-0,36
Q9R0Q1	SYTL4	5,0023E-01	0,32
Q9JME7	TPC2L	5,0058E-01	-0,36
Q6PIU9	YJ005	5,0070E-01	0,13
Q8R1N4	NUDC3	5,0107E-01	0,32

Q9D3D9	ATPD	5,0181E-01	0,16
Q9D5T0	ATAD1	5,0236E-01	0,07
Q60759	GCDH	5,0288E-01	0,11
Q91WP6	SPA3N	5,0311E-01	-0,37
Q8CGA0	PPM1F	5,0318E-01	0,16
Q8R180	ERO1A	5,0327E-01	-0,42
Q8K3W0	BABA2	5,0352E-01	0,21
P42230	STA5A	5,0401E-01	0,10
O70325	GPX4	5,0410E-01	0,11
Q9R226	KHDR3	5,0434E-01	0,24
Q9JIQ3	DBLOH	5,0452E-01	0,16
P16627	HS71L	5,0453E-01	0,26
P51656	DHB1	5,0508E-01	-0,36
Q99K70	RRAGC	5,0540E-01	0,11
Q99LJ6	GPX7	5,0565E-01	-0,42
Q8K0Q5	RHG18	5,0566E-01	0,20
Q62193	RFA2	5,0594E-01	-0,42
Q91WM2	HDHD5	5,0627E-01	0,13
Q9WV54	ASAH1	5,0635E-01	0,02
Q791T5	MTCH1	5,0647E-01	0,21
P49962	SRP09	5,0647E-01	0,21
Q9JHH9	COPZ2	5,0650E-01	0,21
Q8BG67	EFR3A	5,0650E-01	0,21
Q5SUC9	SCO1	5,0650E-01	0,21
Q62188	DPYL3	5,0670E-01	-0,26
O35469	3BHS6	5,0691E-01	0,04
Q62425	NDUA4	5,0709E-01	0,13
Q9CQE7	ERGI3	5,0709E-01	0,13
Q8BG32	PSD11	5,0774E-01	-0,02
Q61753	SERA	5,0795E-01	-0,31
Q9QXZ0	MACF1	5,0823E-01	0,20
O89053	COR1A	5,0842E-01	0,05
P36993	PPM1B	5,0843E-01	0,23
Q9QY36	NAA10	5,0850E-01	-0,42
Q61599	GDIR2	5,0855E-01	-0,36
P11679	K2C8	5,0897E-01	-0,28
Q9R1T4	SEPT6	5,0897E-01	0,20
Q8C8U0	LIPB1	5,0908E-01	0,16
Q62426	CYTB	5,0917E-01	0,11
Q8BM55	TM214	5,0935E-01	0,21
Q9D710	TMX2	5,0935E-01	0,21
Q9JLR9	HIG1A	5,0935E-01	0,21
Q9Z1Q2	ABHGA	5,0935E-01	0,21

Q9WTX8	MD1L1	5,0957E-01	-0,42
P70236	MP2K6	5,1046E-01	0,22
P17047	LAMP2	5,1071E-01	-0,42
Q80WQ2	VAC14	5,1082E-01	-0,42
Q8BVI5	STX16	5,1089E-01	0,32
Q06138	CAB39	5,1089E-01	0,13
P31230	AIMP1	5,1119E-01	-0,35
Q9JLM9	GRB14	5,1192E-01	0,07
Q9JIK5	DDX21	5,1203E-01	-0,32
Q61792	LASP1	5,1220E-01	-0,35
P01897	HA1L	5,1252E-01	-0,40
P12367	KAP2	5,1297E-01	-0,38
P35922	FMR1	5,1302E-01	-0,37
Q8BWG8	ARRB1	5,1312E-01	-0,38
Q9Z1W9	STK39	5,1341E-01	-0,36
P61222	ABCE1	5,1371E-01	-0,34
E9PVA8	GCN1	5,1385E-01	-0,46
Q80XI3	IF4G3	5,1427E-01	0,14
P70202	LXN	5,1501E-01	-0,38
Q61122	NAB1	5,1509E-01	0,21
Q8R1G6	PDLI2	5,1509E-01	0,21
P51175	PPOX	5,1509E-01	0,21
Q9CYN2	SPCS2	5,1540E-01	0,08
P26350	PTMA	5,1566E-01	-0,42
P56380	AP4A	5,1566E-01	-0,42
Q5XKN4	JAGN1	5,1566E-01	-0,42
Q8BJ05	ZC3HE	5,1567E-01	-0,42
P59235	NUP43	5,1567E-01	-0,38
Q04997	INHA	5,1643E-01	0,04
Q61555	FBN2	5,1669E-01	-0,66
Q3UPH1	PRRC1	5,1677E-01	-0,38
Q91YR7	PRP6	5,1685E-01	-0,34
Q9JLQ2	GIT2	5,1717E-01	-0,40
Q9CX80	CYGB	5,1721E-01	0,12
Q64324	STXB2	5,1723E-01	0,10
Q9JKB1	UCHL3	5,1774E-01	0,06
Q9EPK7	XPO7	5,1818E-01	-0,33
Q62348	TSN	5,1835E-01	0,06
Q6PAV2	HERC4	5,1856E-01	-0,40
Q9CQW1	YKT6	5,1914E-01	-0,37
Q02053	UBA1	5,1979E-01	-0,24
P63028	TCTP	5,2022E-01	-0,01
P01843	LAC1	5,2032E-01	-0,42

Q8BUV3	GEPH	5,2043E-01	-0,37
P97465	DOK1	5,2064E-01	-0,36
Q6P4T2	U520	5,2075E-01	-0,36
P31786	ACBP	5,2096E-01	0,08
Q62351	TFR1	5,2118E-01	0,15
Q9WVL0	MAAI	5,2139E-01	0,08
Q8BU31	RAP2C	5,2202E-01	0,20
Q80X95	RRAGA	5,2216E-01	0,13
Q91ZJ5	UGPA	5,2238E-01	0,03
P61759	PFD3	5,2270E-01	0,10
Q9Z204	HNRPC	5,2287E-01	-0,03
Q8BHL8	PSMF1	5,2289E-01	-0,42
P62806	H4	5,2305E-01	-0,06
P23242	CXA1	5,2374E-01	0,05
Q3URD3	SLMAP	5,2409E-01	0,10
P62301	RS13	5,2415E-01	-0,37
Q8VH51	RBM39	5,2481E-01	0,05
P68254	1433T	5,2484E-01	-0,05
Q8VC19	HEM1	5,2505E-01	0,32
Q8CHY6	P66A	5,2522E-01	-0,42
Q8VE92	RBM4B	5,2538E-01	-0,40
Q8R050	ERF3A	5,2594E-01	-0,33
P68372	TBB4B	5,2629E-01	-0,28
Q9D8N0	EF1G	5,2629E-01	-0,06
P61211	ARL1	5,2649E-01	0,10
Q9R0A0	PEX14	5,2654E-01	0,15
P70303	PYRG2	5,2681E-01	-0,36
Q9CR62	M2OM	5,2687E-01	-0,37
Q8VE96	S35F6	5,2724E-01	-0,42
Q61550	RAD21	5,2746E-01	0,08
P53702	CCHL	5,2791E-01	0,20
Q9CQR4	ACO13	5,2791E-01	0,20
Q9JIW9	RALB	5,2792E-01	-0,40
P48453	PP2BB	5,2813E-01	-0,26
O35704	SPTC1	5,2821E-01	0,15
Q9D7N9	APMAP	5,2825E-01	-0,36
O70370	CATS	5,2896E-01	0,12
P22907	HEM3	5,2897E-01	-0,39
Q9DC70	NDUS7	5,2955E-01	0,15
Q8R574	KPRB	5,2979E-01	-0,40
P97823	LYPA1	5,2982E-01	0,15
Q9WTP6	KAD2	5,2986E-01	0,01
O88271	CFDP1	5,2997E-01	0,12

Q99J36	THUM1	5,3009E-01	-0,39
Q9CZW5	TOM70	5,3026E-01	0,01
Q9R0P6	SC11A	5,3027E-01	0,12
O89086	RBM3	5,3056E-01	-0,36
Q11011	PSA	5,3083E-01	-0,30
Q61210	ARHG1	5,3089E-01	-0,39
P99027	RLA2	5,3108E-01	-0,36
Q9D2V8	MFS10	5,3146E-01	0,20
P15379	CD44	5,3146E-01	0,20
P14901	HMOX1	5,3194E-01	-0,36
P35762	CD81	5,3219E-01	0,12
P16332	MUTA	5,3223E-01	0,00
Q6PAR5	GAPD1	5,3225E-01	0,12
Q8C0L6	PAOX	5,3267E-01	0,15
Q8CCS6	PABP2	5,3290E-01	0,07
P25911	LYN	5,3298E-01	0,20
Q9R1P0	PSA4	5,3374E-01	0,00
Q9QYB5	ADDG	5,3436E-01	0,03
P60603	ROMO1	5,3517E-01	0,20
Q9DCF9	SSRG	5,3517E-01	0,20
Q99L27	GMPR2	5,3517E-01	0,20
Q9DB16	CB39L	5,3517E-01	0,20
Q8BIG7	CMTD1	5,3522E-01	0,20
Q9CPZ6	ORML3	5,3522E-01	0,20
Q9WUR2	ECI2	5,3534E-01	0,03
Q60930	VDAC2	5,3591E-01	-0,02
Q68FH4	GALK2	5,3606E-01	0,07
Q9WVL3	S12A7	5,3650E-01	-0,40
Q9Z2M7	PMM2	5,3676E-01	0,05
Q8BMJ3	IF1AX	5,3705E-01	0,20
P68369	TBA1A	5,3788E-01	0,05
Q9Z1N5	DX39B	5,3860E-01	-0,06
Q811D0	DLG1	5,3875E-01	0,11
Q6P3A8	ODBB	5,3878E-01	0,20
Q3UDW8	HGNAT	5,3878E-01	0,20
Q9Z266	SNAPN	5,3878E-01	0,20
Q99J95	CDK9	5,3901E-01	0,20
P09411	PGK1	5,3937E-01	-0,07
Q91X97	NCALD	5,3969E-01	-0,40
Q3UYV9	NCBP1	5,3978E-01	-0,32
Q9Z0E6	GBP2	5,3989E-01	-0,33
P70699	LYAG	5,4141E-01	-0,34
Q60737	CSK21	5,4215E-01	0,01

Q8N7N5	DCAF8	5,4279E-01	-0,38
Q8CAS9	PARP9	5,4339E-01	-0,68
Q9CYZ2	TPD54	5,4424E-01	0,02
O08848	RO60	5,4519E-01	-0,38
P55194	3BP1	5,4519E-01	-0,38
Q9EQC5	SCYL1	5,4519E-01	-0,38
O35074	PTGIS	5,4568E-01	-0,32
Q3TIR3	RIC8A	5,4583E-01	-0,39
Q9EQH3	VPS35	5,4584E-01	-0,04
Q8BH59	CMC1	5,4667E-01	-0,03
Q9D6F9	TBB4A	5,4722E-01	-0,38
Q91YP0	L2HDH	5,4786E-01	-0,39
Q9D4H8	CUL2	5,4802E-01	0,09
Q6ZPU9	KBP	5,4852E-01	-0,38
Q61205	PA1B3	5,4870E-01	-0,38
O35309	NMI	5,4909E-01	-0,39
Q9R1P4	PSA1	5,4967E-01	-0,01
Q6PEB6	PHOCN	5,4996E-01	0,09
Q7TSV4	PGM2	5,5021E-01	-0,03
Q9D2M8	UB2V2	5,5079E-01	-0,31
Q149F3	ERF3B	5,5104E-01	-0,37
Q80YX1	TENA	5,5190E-01	0,07
O89103	C1QR1	5,5211E-01	-0,39
P04117	FABP4	5,5228E-01	-0,39
Q71LX4	TLN2	5,5300E-01	0,00
O88456	CPNS1	5,5322E-01	0,06
P05480	SRC	5,5336E-01	-0,38
Q9EQ80	NIF3L	5,5338E-01	-0,36
Q921M3	SF3B3	5,5358E-01	-0,27
P26039	TLN1	5,5433E-01	-0,24
O88952	LIN7C	5,5496E-01	0,14
Q8R409	HEXI1	5,5552E-01	-0,38
Q9D142	NUD14	5,5552E-01	-0,38
Q9CR09	UFC1	5,5552E-01	-0,38
Q9JMA2	TGT	5,5567E-01	-0,37
P62702	RS4X	5,5710E-01	-0,05
P63101	1433Z	5,5711E-01	-0,07
Q9QZB7	ARP10	5,5747E-01	0,11
Q8BK64	AHSA1	5,5770E-01	-0,30
Q60847	COCA1	5,5778E-01	-0,39
O09117	SYPL1	5,5832E-01	0,14
P42932	TCPQ	5,5837E-01	-0,08
Q9EP72	EMC7	5,5872E-01	-0,39

Q9Z2V5	HDAC6	5,5889E-01	-0,38
Q3TIU4	PDE12	5,5889E-01	-0,38
Q9CR26	VTA1	5,5889E-01	-0,38
P97471	SMAD4	5,5889E-01	-0,38
Q9Z2W0	DNPEP	5,5977E-01	-0,06
Q9Z2A7	DGAT1	5,5981E-01	0,32
Q61093	CY24B	5,5981E-01	0,32
Q91YR9	PTGR1	5,6014E-01	0,04
Q8BVE3	VATH	5,6021E-01	0,04
P97765	WBP2	5,6072E-01	0,11
P70460	VASP	5,6096E-01	-0,35
O35387	HAX1	5,6123E-01	0,32
Q923X4	GLRX2	5,6123E-01	0,32
P70255	NFIC	5,6125E-01	-0,38
Q3THE2	ML12B	5,6149E-01	0,01
Q9QYR9	ACOT2	5,6188E-01	0,00
P70302	STIM1	5,6196E-01	0,14
P70318	TIAR	5,6196E-01	-0,35
Q61206	PA1B2	5,6212E-01	0,14
Q9BCZ4	SELS	5,6236E-01	0,18
Q8R3Q6	CCD58	5,6239E-01	0,18
Q64514	TPP2	5,6249E-01	-0,01
Q91V76	CK054	5,6314E-01	0,04
Q9D7X3	DUS3	5,6396E-01	0,07
Q9D6Y9	GLGB	5,6476E-01	-0,36
Q9DAW6	PRP4	5,6477E-01	-0,35
P47199	QOR	5,6513E-01	-0,34
Q9D2C2	SAAL1	5,6570E-01	-0,30
Q8C0L0	TMX4	5,6587E-01	-0,38
Q9D154	ILEUA	5,6594E-01	-0,05
Q64282	IFIT1	5,6595E-01	-1,26
Q80V26	IMPA3	5,6644E-01	-0,34
Q8BFZ9	ERLN2	5,6687E-01	-0,31
Q8BYA0	TBCD	5,6706E-01	-0,34
P29595	NEDD8	5,6713E-01	0,18
Q6PGF7	EXOC8	5,6713E-01	0,18
B1AVZ0	UPP	5,6713E-01	0,18
Q9CQH3	NDUB5	5,6713E-01	0,18
Q5M8N0	CNRP1	5,6713E-01	0,18
Q9DB96	NGDN	5,6713E-01	0,18
Q9CQZ5	NDUA6	5,6713E-01	0,18
Q80ZS3	RT26	5,6718E-01	0,18
Q9CR16	PPID	5,6791E-01	-0,32

O88291	ZN326	5,6796E-01	-0,32
P63242	IF5A1	5,6803E-01	-0,05
Q99JF8	PSIP1	5,6804E-01	-0,34
Q9D4J1	EFHD1	5,6880E-01	0,06
Q9DCB8	ISCA2	5,6920E-01	-0,38
Q9JI75	NQO2	5,6938E-01	-0,34
B2RRE7	OTUD4	5,6946E-01	-0,38
Q80UM3	NAA15	5,7045E-01	-0,33
O08663	MAP2	5,7059E-01	0,01
P59017	B2L13	5,7065E-01	0,02
P12970	RL7A	5,7081E-01	-0,31
Q9R0P5	DEST	5,7096E-01	-0,04
P16125	LDHB	5,7171E-01	-0,26
Q9CT10	RANB3	5,7183E-01	-0,32
P41241	CSK	5,7192E-01	-0,37
P63154	CRNL1	5,7220E-01	-0,37
P19221	THRB	5,7226E-01	-0,33
P35550	FBRL	5,7237E-01	-0,03
Q3UMF0	COBL1	5,7245E-01	0,04
Q8K2Y9	CCM2	5,7253E-01	0,32
Q9DCJ1	LST8	5,7313E-01	-0,37
Q922F4	TBB6	5,7326E-01	-0,25
Q921U8	SMTN	5,7345E-01	-0,35
Q9WVE8	PACN2	5,7372E-01	-0,32
Q9WVJ9	FBLN4	5,7392E-01	-0,36
Q9ESX5	DKC1	5,7394E-01	-0,35
P62962	PROF1	5,7445E-01	-0,05
Q8CG76	ARK72	5,7487E-01	0,01
O09131	GSTO1	5,7547E-01	0,04
Q8VEJ4	NLE1	5,7549E-01	-0,37
Q9EQ61	PESC	5,7665E-01	0,18
E9QAT4	SC16A	5,7665E-01	0,18
Q99JB8	PACN3	5,7684E-01	0,05
Q8R081	HNRPL	5,7763E-01	-0,25
Q6NTA4	RRAGB	5,7807E-01	-0,25
Q8R5C5	ACTY	5,7883E-01	0,03
Q9R0Y5	KAD1	5,7889E-01	0,32
Q6NV83	SR140	5,7953E-01	-0,63
Q9Z1Z0	USO1	5,7997E-01	-0,03
P97429	ANXA4	5,8092E-01	-0,27
Q9ERK4	XPO2	5,8143E-01	-0,06
Q61136	PRP4B	5,8154E-01	0,18
P11276	FINC	5,8215E-01	-0,25

Q9Z2N8	ACL6A	5,8254E-01	-0,03
P62264	RS14	5,8274E-01	-0,01
Q9WVJ5	CRBB1	5,8282E-01	0,32
P24668	MPRD	5,8313E-01	-0,36
P55937	GOGA3	5,8315E-01	0,17
Q9CQI6	COTL1	5,8323E-01	-0,36
Q61024	ASNS	5,8327E-01	-0,37
Q8K2Q7	BROX	5,8339E-01	-0,36
Q8CHK3	MBOA7	5,8367E-01	0,13
Q61133	GSTT2	5,8372E-01	0,13
Q70IV5	SYNEM	5,8398E-01	-0,33
P54071	IDHP	5,8401E-01	-0,26
Q9CZN7	GLYM	5,8429E-01	-0,03
Q3UIZ8	MYLK3	5,8438E-01	0,17
Q99K23	UFSP2	5,8565E-01	0,13
Q99M87	DNJA3	5,8576E-01	0,06
Q9Z2D6	MECP2	5,8585E-01	0,01
Q6PB44	PTN23	5,8593E-01	-0,73
Q9D281	NXP20	5,8686E-01	-0,35
Q3THK3	T2FA	5,8687E-01	-0,36
P62192	PRS4	5,8706E-01	-0,27
Q8R3N6	THOC1	5,8709E-01	-0,35
Q91YJ2	SNX4	5,8712E-01	-0,36
Q99JX3	GORS2	5,8732E-01	0,02
P60335	PCBP1	5,8756E-01	-0,07
Q9DAK9	PHP14	5,8869E-01	0,06
P10761	ZP3	5,8899E-01	-0,28
Q8BJ90	ZN771	5,8943E-01	0,32
Q9D0D3	PAPD1	5,8943E-01	0,32
P26231	CTNA1	5,8970E-01	-0,26
Q6GU68	ISLR	5,9032E-01	0,13
Q99KY4	GAK	5,9047E-01	0,17
Q8R3D1	TBC13	5,9065E-01	-0,36
Q8CFE4	SCYL2	5,9080E-01	0,17
Q62186	SSRD	5,9089E-01	0,06
Q91WD5	NDUS2	5,9096E-01	-0,30
Q63844	MK03	5,9151E-01	-0,01
P27046	MA2A1	5,9169E-01	-0,02
Q9DBE0	CSAD	5,9171E-01	0,07
Q920E5	FPPS	5,9213E-01	-0,04
Q8R3H7	HS2ST	5,9235E-01	-0,19
Q9CR58	KMCP1	5,9243E-01	0,13
P70398	USP9X	5,9371E-01	-0,34

P01921	HB2D	5,9388E-01	0,20
P56959	FUS	5,9399E-01	-0,03
P62880	GBB2	5,9443E-01	-0,04
Q8R164	BPHL	5,9450E-01	0,01
Q4FK66	PR38A	5,9476E-01	-0,36
Q9D0G0	RT30	5,9476E-01	-0,36
Q8BIQ5	CSTF2	5,9480E-01	-0,35
Q9JKF1	IQGA1	5,9568E-01	-0,25
Q3U7R1	ESYT1	5,9644E-01	-0,30
Q8BHN0	PPM1L	5,9690E-01	0,17
Q9CXY1	TM175	5,9690E-01	0,17
Q9ESP1	SDF2L	5,9690E-01	0,17
Q9D7P6	ISCU	5,9690E-01	0,17
Q8BR90	CE051	5,9691E-01	0,17
P41233	ABCA1	5,9691E-01	0,17
Q9CRA8	EXOS5	5,9694E-01	-0,36
Q9QZN4	FBX6	5,9697E-01	0,17
Q8K1M6	DNM1L	5,9818E-01	-0,28
Q9DC61	MPPA	5,9838E-01	0,00
Q9D1R1	T126B	5,9849E-01	0,32
O35593	PSDE	5,9865E-01	0,00
Q8BGX2	TIM29	5,9870E-01	-0,36
Q91WM3	U3IP2	5,9870E-01	-0,36
P10833	RRAS	5,9918E-01	0,08
P29788	VTNC	5,9931E-01	-0,33
Q9Z2U1	PSA5	5,9957E-01	-0,03
Q6A0A9	F120A	6,0025E-01	-0,03
P16675	PPGB	6,0047E-01	-0,34
P39053	DYN1	6,0052E-01	-0,35
P50396	GDIA	6,0058E-01	-0,05
Q9JKC8	AP3M1	6,0064E-01	-0,34
O70194	EIF3D	6,0084E-01	-0,28
P62331	ARF6	6,0087E-01	0,07
Q2YDW2	MSTO1	6,0120E-01	-0,36
P97300	NPTN	6,0137E-01	-0,36
P20060	HEXB	6,0144E-01	-0,36
P62270	RS18	6,0154E-01	-0,02
Q8BGH2	SAM50	6,0285E-01	-0,01
Q68FF6	GIT1	6,0291E-01	-0,32
Q3U3R4	LMF1	6,0303E-01	-0,36
Q9CQ49	NCBP2	6,0328E-01	0,17
Q8C5L6	INP5K	6,0328E-01	0,17
Q9DAS9	GBG12	6,0328E-01	0,17

Q9CQ10	CHMP3	6,0328E-01	0,17
Q9JHK5	PLEK	6,0328E-01	0,17
O88983	STX8	6,0328E-01	0,17
Q9DCG9	TR112	6,0328E-01	0,17
Q60739	BAG1	6,0334E-01	0,07
Q80TL0	PPM1E	6,0337E-01	0,17
Q3T9E4	TGTP2	6,0337E-01	0,17
O09106	HDAC1	6,0364E-01	0,01
P28798	GRN	6,0372E-01	-0,42
Q9QWR8	NAGAB	6,0410E-01	-0,32
Q9WU81	G6PT3	6,0423E-01	0,17
Q8BP48	MAP11	6,0426E-01	-0,31
Q920Q6	MSI2H	6,0428E-01	0,04
Q9JLV6	PNKP	6,0446E-01	-0,32
P01898	HA10	6,0488E-01	-0,32
Q91WK2	EIF3H	6,0513E-01	-0,31
Q0VGB7	PP4R2	6,0527E-01	-0,32
Q8R3G1	PP1R8	6,0550E-01	-0,31
Q8C7Q4	RBM4	6,0726E-01	-0,35
Q02257	PLAK	6,0871E-01	-0,62
Q9DBS2	TPRGL	6,0939E-01	0,07
Q9Z0Y1	DCTN3	6,0960E-01	0,17
Q99M01	SYFM	6,0960E-01	0,17
Q9WU56	TRUA	6,1186E-01	-0,33
Q9CXV1	DHSD	6,1203E-01	-0,33
Q8BWL5	RBMS3	6,1203E-01	-0,33
Q9CQN6	TM14C	6,1203E-01	-0,33
Q3V0C5	UBP48	6,1203E-01	-0,33
Q3UFF7	LYPL1	6,1203E-01	-0,33
Q8K274	KT3K	6,1203E-01	-0,33
Q9DCS2	MTL26	6,1251E-01	0,09
G5E870	TRIPC	6,1325E-01	0,05
Q9CZ30	OLA1	6,1329E-01	0,05
Q8VCR7	ABHEB	6,1356E-01	0,09
Q8VD62	CK068	6,1379E-01	0,09
Q9CR39	WIPI3	6,1405E-01	-0,19
Q9JMC3	DNJA4	6,1426E-01	0,13
Q6ZWU9	RS27	6,1504E-01	0,09
P18293	ANPRA	6,1505E-01	-0,03
O70551	SRPK1	6,1511E-01	-0,34
O88851	RBBP9	6,1556E-01	-0,34
Q9QXK3	COPG2	6,1568E-01	-0,02
Q8QZT1	THIL	6,1571E-01	-0,08

P32233	DRG1	6,1574E-01	-0,01
Q8BKE6	CP20A	6,1610E-01	0,05
O88561	S27A3	6,1631E-01	0,09
Q64674	SPEE	6,1660E-01	-0,27
Q9CQE8	RTRAF	6,1670E-01	-0,32
P40142	TKT	6,1749E-01	-0,22
Q8R4H2	ARHGC	6,1759E-01	-0,33
Q3UMT1	PP12C	6,1759E-01	-0,33
Q8BMS9	RASF2	6,1759E-01	-0,33
Q8R322	GLE1	6,1766E-01	-0,33
Q61189	ICLN	6,1766E-01	-0,33
Q6PGB6	NAA50	6,1766E-01	-0,33
P26369	U2AF2	6,1804E-01	-0,28
Q08369	GATA4	6,1805E-01	-0,32
O88544	CSN4	6,1842E-01	-0,04
Q9JMH6	TRXR1	6,1868E-01	-0,28
P59708	SF3B6	6,1876E-01	-0,34
P80317	TCPZ	6,1951E-01	-0,07
Q9CWU6	UQCC1	6,2031E-01	0,12
Q9Z2Y8	PLPHP	6,2038E-01	0,05
Q91VM9	IPYR2	6,2115E-01	-0,02
P83870	PHF5A	6,2182E-01	0,12
P48428	TBCA	6,2216E-01	-0,34
Q3TJZ6	FA98A	6,2218E-01	-0,33
P52875	TM165	6,2231E-01	0,12
Q8CI75	DI3L2	6,2231E-01	0,12
P51432	PLCB3	6,2248E-01	-0,36
Q9WVK4	EHD1	6,2262E-01	-0,28
P14602	HSPB1	6,2306E-01	-0,36
Q5SSI6	UTP18	6,2337E-01	-0,33
Q9QZS0	CO4A3	6,2337E-01	-0,33
Q9EPE9	AT131	6,2418E-01	0,14
Q9ES97	RTN3	6,2478E-01	0,03
Q9JKF7	RM39	6,2494E-01	0,12
Q80UP5	AN13A	6,2494E-01	0,12
P01644	KV5AB	6,2532E-01	-0,34
Q6PDL0	DC1L2	6,2602E-01	0,00
P62821	RAB1A	6,2629E-01	-0,05
Q8BVL3	SNX17	6,2708E-01	0,15
Q3UX10	TBAL3	6,2750E-01	-0,33
Q8K4G5	ABLM1	6,2787E-01	-0,58
Q5SUF2	LC7L3	6,2823E-01	-0,29
Q8BHL5	ELMO2	6,2839E-01	0,14

Q01768	NDKB	6,2864E-01	-0,26
Q9ER38	TOR3A	6,2882E-01	-0,33
Q60855	RIPK1	6,2882E-01	-0,33
Q922R8	PDIA6	6,2884E-01	-0,09
Q6PD26	PIGS	6,2885E-01	0,02
Q00612	G6PD1	6,2937E-01	-0,06
Q91V64	ISOC1	6,2960E-01	-0,29
P61620	S61A1	6,2973E-01	0,01
Q3URS9	CCD51	6,2980E-01	0,23
P21956	MFGM	6,3102E-01	-0,33
Q9R1P3	PSB2	6,3114E-01	0,02
O55126	NIPS2	6,3238E-01	0,12
Q9D0J4	ARL2	6,3260E-01	0,10
O35129	PHB2	6,3264E-01	-0,06
Q9WTX2	PRKRA	6,3340E-01	-0,33
P19253	RL13A	6,3351E-01	0,06
P17879	HS71B	6,3406E-01	-0,07
Q3UTJ2	SRBS2	6,3465E-01	-0,28
P17439	GLCM	6,3479E-01	0,06
Q9CRD0	OCAD1	6,3501E-01	0,02
Q9D2V7	CORO7	6,3521E-01	-0,33
Q810B6	ANFY1	6,3609E-01	0,00
Q9DC50	OCTC	6,3612E-01	0,00
P68368	TBA4A	6,3613E-01	-0,23
Q8VHN8	TIRR	6,3617E-01	0,15
Q91W52	TMM19	6,3617E-01	0,15
Q9CZB0	C560	6,3617E-01	0,15
Q61072	ADAM9	6,3621E-01	0,15
Q9WTN0	GGPPS	6,3621E-01	0,15
P11835	ITB2	6,3621E-01	0,15
Q9CR51	VATG1	6,3623E-01	0,15
Q8K354	CBR3	6,3657E-01	0,00
Q00898	A1AT5	6,3731E-01	-0,29
Q9EQI8	RM46	6,3745E-01	-0,32
P54227	STMN1	6,3745E-01	-0,32
Q99KQ4	NAMPT	6,3785E-01	-0,26
Q91YW3	DNJC3	6,3805E-01	-0,03
Q9D5V5	CUL5	6,3855E-01	-0,30
P57759	ERP29	6,3869E-01	-0,29
Q8VE88	F1142	6,3872E-01	-0,32
Q9JHP7	PLGT2	6,3872E-01	-0,32
Q8BG81	PDIP3	6,3902E-01	-0,31
Q8K019	BCLF1	6,3902E-01	-0,32

Q00PI9	HNRL2	6,3912E-01	-0,25
O89110	CASP8	6,3915E-01	-0,33
Q9DBG6	RPN2	6,3918E-01	-0,07
Q6WVG3	KCD12	6,3949E-01	-0,02
Q8BP92	RCN2	6,3964E-01	-0,03
P35278	RAB5C	6,4110E-01	-0,04
Q6P1B1	XPP1	6,4128E-01	-0,03
Q99P31	HPBP1	6,4144E-01	-0,32
Q07813	BAX	6,4146E-01	-0,29
Q9QXT0	CNPY2	6,4198E-01	-0,03
Q02788	CO6A2	6,4211E-01	-0,06
Q8VCM7	FIBG	6,4257E-01	-0,24
Q02780	NFIA	6,4292E-01	-0,32
Q02248	CTNB1	6,4298E-01	-0,05
Q9JIG7	CCD22	6,4319E-01	-0,31
Q9ERF3	WDR61	6,4335E-01	-0,30
O89079	COPE	6,4374E-01	-0,27
Q9WTQ8	TIM23	6,4378E-01	0,04
Q9D6R2	IDH3A	6,4386E-01	-0,05
P17182	ENOA	6,4393E-01	-0,22
Q62189	SNRPA	6,4437E-01	0,04
P28659	CELF1	6,4451E-01	-0,30
Q8BFY6	PEF1	6,4459E-01	-0,32
O09172	GSH0	6,4459E-01	-0,31
Q3TW96	UAP1L	6,4521E-01	-0,30
Q9JKN1	ZNT7	6,4535E-01	0,15
Q99JH8	ERD21	6,4535E-01	0,15
Q9JHS3	LTOR2	6,4535E-01	0,15
Q91X52	DCXR	6,4554E-01	0,15
Q9D1H7	GET4	6,4574E-01	0,04
Q91YI0	ARLY	6,4587E-01	-0,26
Q04592	PCSK5	6,4610E-01	-0,32
Q78JE5	FBX22	6,4785E-01	-0,31
Q923D4	SF3B5	6,4847E-01	-0,32
Q9D6J5	NDUB8	6,4925E-01	-0,32
Q9Z1Q9	SYVC	6,5080E-01	-0,25
P61804	DAD1	6,5120E-01	0,10
Q9D0R4	DDX56	6,5175E-01	0,10
Q99KN9	EPN4	6,5225E-01	0,02
P51863	VA0D1	6,5242E-01	0,01
P61027	RAB10	6,5282E-01	0,00
P38060	HMGCL	6,5303E-01	0,04
Q9CZV5	ST65G	6,5308E-01	-0,26

P80315	TCPD	6,5381E-01	-0,23
P62305	RUXE	6,5407E-01	-0,32
Q8VE95	CH082	6,5407E-01	-0,32
Q60823	AKT2	6,5426E-01	0,14
Q8R1U1	COG4	6,5464E-01	-0,32
P54823	DDX6	6,5464E-01	-0,26
Q8C166	CPNE1	6,5479E-01	-0,30
A2BDX3	MOCS3	6,5574E-01	0,01
Q6ZWX6	IF2A	6,5589E-01	-0,24
Q99JI4	PSMD6	6,5592E-01	-0,06
O09005	DEGS1	6,5710E-01	-0,32
Q921S7	RM37	6,5710E-01	-0,32
Q9DCH4	EIF3F	6,5723E-01	-0,27
Q99JY8	PLPP3	6,5725E-01	0,10
O35206	COFA1	6,5772E-01	0,15
P47791	GSHR	6,5780E-01	-0,03
Q9D662	SC23B	6,5787E-01	-0,27
Q91ZX7	LRP1	6,5791E-01	-0,13
P47754	CAZA2	6,5903E-01	-0,06
Q8R0X7	SGPL1	6,5959E-01	-0,28
Q99LD9	EI2BB	6,6001E-01	-0,31
Q9Z2G6	SE1L1	6,6025E-01	0,05
P19783	COX41	6,6065E-01	-0,03
Q4KML4	ABRAL	6,6101E-01	0,05
P50516	VATA	6,6142E-01	-0,07
P15864	H12	6,6148E-01	-0,29
Q8BWY3	ERF1	6,6160E-01	-0,28
Q8VE37	RCC1	6,6226E-01	-0,02
Q61598	GDIB	6,6304E-01	-0,09
Q3UPL0	SC31A	6,6313E-01	-0,07
Q99J39	DCMC	6,6322E-01	0,10
Q9R0Q9	MPU1	6,6333E-01	-0,32
Q505B7	ARCH	6,6347E-01	-0,26
Q91ZE0	TMLH	6,6347E-01	-0,26
P47811	MK14	6,6347E-01	0,07
Q3UFK8	FRMD8	6,6352E-01	0,10
Q99KB8	GLO2	6,6352E-01	0,10
Q6P069	SORCN	6,6365E-01	0,05
Q9WTI7	MYO1C	6,6381E-01	-0,08
Q9CSN1	SNW1	6,6393E-01	-0,28
Q99PL6	UBXN6	6,6394E-01	-0,31
Q8K2C9	HACD3	6,6417E-01	-0,03
Q8R1B4	EIF3C	6,6531E-01	-0,07

Q7M6Y3	PICAL	6,6551E-01	-0,28
Q9Z1X4	ILF3	6,6650E-01	-0,07
Q80UJ7	RB3GP	6,6698E-01	-0,32
O09061	PSB1	6,6704E-01	-0,28
Q99JY4	TRABD	6,6775E-01	0,13
Q61387	COX7R	6,6775E-01	0,13
Q99MJ9	DDX50	6,6775E-01	0,13
Q9ERL9	GCYA1	6,6775E-01	0,13
Q924L1	LTMD1	6,6775E-01	0,13
O35643	AP1B1	6,6781E-01	-0,28
Q99JX7	NXF1	6,6793E-01	-0,28
Q61033	LAP2A	6,6797E-01	-0,25
P10126	EF1A1	6,6809E-01	-0,22
Q6Q477	AT2B4	6,6817E-01	-0,29
O70435	PSA3	6,6869E-01	-0,28
Q9Z0R4	ITSN1	6,6870E-01	-0,57
Q8C6G8	WDR26	6,6873E-01	-0,31
P61226	RAP2B	6,6878E-01	0,10
P16045	LEG1	6,6954E-01	-0,07
Q8BYU6	TOIP2	6,7006E-01	0,14
Q99PW4	PRPK	6,7025E-01	-0,31
A2AN08	UBR4	6,7054E-01	-0,68
P46935	NEDD4	6,7217E-01	-0,23
O70309	ITB5	6,7219E-01	-0,39
P00493	HPRT	6,7266E-01	-0,28
P99029	PRDX5	6,7307E-01	-0,25
Q9D1G1	RAB1B	6,7314E-01	-0,03
P62748	HPCL1	6,7383E-01	0,03
Q640N1	AEBP1	6,7386E-01	-0,36
O55101	SNG2	6,7407E-01	-0,26
Q8C3W1	CA198	6,7407E-01	-0,26
Q9D902	T2EB	6,7407E-01	-0,26
Q8BVU5	NUDT9	6,7421E-01	-0,31
P21126	UBL4A	6,7465E-01	0,15
Q06180	PTN2	6,7478E-01	-0,31
P28352	APEX1	6,7479E-01	-0,26
P47757	CAPZB	6,7514E-01	-0,08
Q5U458	DJC11	6,7539E-01	-0,28
Q3TCH7	CUL4A	6,7574E-01	-0,29
O35857	TIM44	6,7623E-01	-0,05
Q8VEH5	EPMIP	6,7643E-01	-0,28
Q9CRB8	MTFP1	6,7728E-01	0,06
O55106	STRN	6,7760E-01	-0,26

Q9R062	GLYG	6,7794E-01	0,01
O88685	PRS6A	6,7812E-01	-0,23
Q8BH24	TM9S4	6,7840E-01	-0,30
P62259	1433E	6,7859E-01	-0,11
Q922K7	NOP2	6,7863E-01	0,06
P08228	SODC	6,7866E-01	-0,04
O08810	U5S1	6,7929E-01	-0,09
P54726	RD23A	6,7951E-01	0,07
Q9JHU4	DYHC1	6,7975E-01	-0,10
Q9D6J6	NDUV2	6,7985E-01	-0,28
Q9Z0K8	VNN1	6,8026E-01	-0,28
Q9Z2Z6	MCAT	6,8043E-01	0,01
Q8BK67	RCC2	6,8048E-01	-0,25
Q99K28	ARFG2	6,8082E-01	-0,01
Q8VHE0	SEC63	6,8107E-01	0,03
P17426	AP2A1	6,8114E-01	-0,05
Q7TNG5	EMAL2	6,8121E-01	-0,03
P56376	ACYP1	6,8192E-01	0,06
P08226	APOE	6,8203E-01	-0,04
Q8BU88	RM22	6,8204E-01	0,13
O35143	ATIF1	6,8204E-01	0,13
Q91X20	ASH2L	6,8204E-01	0,13
Q80VA0	GALT7	6,8204E-01	0,13
Q3TLH4	PRC2C	6,8204E-01	0,13
P49138	MAPK2	6,8204E-01	0,13
Q3UWW6	GA2L3	6,8204E-01	0,13
Q8K201	KCT2	6,8204E-01	0,13
Q9CR50	ZN363	6,8204E-01	0,13
Q3U0S6	RAIN	6,8212E-01	0,13
Q9JJ80	RPF2	6,8212E-01	0,13
Q8R151	ZNFX1	6,8212E-01	0,13
Q5Y5T1	ZDH20	6,8212E-01	0,13
Q9WVC3	CAV2	6,8212E-01	0,13
Q9CZH7	MXRA7	6,8212E-01	0,13
O88559	MEN1	6,8212E-01	0,13
Q925B0	PAWR	6,8220E-01	0,07
Q99KD5	UN45A	6,8223E-01	-0,01
Q9D8S3	ARFG3	6,8315E-01	0,06
Q9DBB8	DHDH	6,8317E-01	-0,28
O55135	IF6	6,8321E-01	-0,28
Q9DCN2	NB5R3	6,8345E-01	-0,25
Q9JKV1	ADRM1	6,8359E-01	-0,01
Q9DB29	IAH1	6,8361E-01	0,06

Q8BGW1	FTO	6,8361E-01	-0,28
Q9D7S9	CHMP5	6,8373E-01	-0,09
Q6ZQI3	MLEC	6,8381E-01	-0,01
Q9JJV2	PROF2	6,8391E-01	0,06
Q9JMD0	ZN207	6,8421E-01	-0,30
P11352	GPX1	6,8456E-01	-0,02
Q9WVF7	DPOE1	6,8463E-01	-0,26
Q9DCH6	ZFAN6	6,8463E-01	-0,26
Q9CWR1	WDR73	6,8463E-01	-0,26
Q8C079	STRP1	6,8463E-01	-0,26
Q8BH93	MISSL	6,8463E-01	-0,26
Q0P678	ZCH18	6,8463E-01	-0,26
Q8K1N4	SPAS2	6,8463E-01	-0,26
Q9QX15	CA3A1	6,8463E-01	-0,26
Q61069	USF1	6,8463E-01	-0,26
Q923B1	DBR1	6,8463E-01	-0,26
Q99ME2	WDR6	6,8463E-01	-0,26
Q6P9N1	HYCCI	6,8463E-01	-0,26
Q9JII6	AK1A1	6,8466E-01	-0,08
Q78JW9	UBFD1	6,8486E-01	-0,29
Q6P5E4	UGGG1	6,8509E-01	-0,08
P97371	PSME1	6,8538E-01	-0,06
Q9WUM4	COR1C	6,8538E-01	-0,02
P53811	PIPNB	6,8542E-01	-0,02
Q921T2	TOIP1	6,8550E-01	0,01
Q921M4	GOGA2	6,8570E-01	-0,29
O70133	DHX9	6,8574E-01	-0,23
Q9CQY6	UQCC2	6,8619E-01	0,06
Q9DCT2	NDUS3	6,8676E-01	-0,02
P62915	TF2B	6,8685E-01	-0,29
Q8R480	NUP85	6,8708E-01	-0,28
Q9D0T1	NH2L1	6,8721E-01	-0,29
P97449	AMPN	6,8826E-01	-0,05
O08582	GTPB1	6,8871E-01	-0,28
Q6PD03	2A5A	6,8922E-01	0,00
Q9WUP7	UCHL5	6,8928E-01	-0,29
Q8C650	40422	6,9011E-01	-0,29
Q00623	APOA1	6,9019E-01	-0,23
Q9CZS1	AL1B1	6,9150E-01	-0,09
P14152	MDHC	6,9160E-01	-0,09
Q9DCT8	CRIP2	6,9179E-01	-0,29
P97807	FUMH	6,9206E-01	-0,24
Q64152	BTF3	6,9213E-01	0,08

O54962	BAF	6,9213E-01	0,08
Q61102	ABCB7	6,9215E-01	0,03
Q9D0W5	PPIL1	6,9221E-01	-0,29
Q9QXS6	DREB	6,9277E-01	-0,28
P26040	EZRI	6,9280E-01	-0,07
O09110	MP2K3	6,9321E-01	-0,29
Q06890	CLUS	6,9334E-01	-0,03
Q99MZ7	PECR	6,9361E-01	-0,29
P62900	RL31	6,9361E-01	-0,29
O70310	NMT1	6,9434E-01	0,01
Q8R001	MARE2	6,9439E-01	0,10
O88447	KLC1	6,9451E-01	-0,01
Q99L43	CDS2	6,9529E-01	0,03
Q6Y5D8	RHG10	6,9552E-01	-0,26
Q64430	ATP7A	6,9552E-01	-0,26
Q8BWQ6	VP35L	6,9554E-01	0,08
P61967	AP1S1	6,9568E-01	0,08
P62627	DLRB1	6,9568E-01	0,08
P34914	HYES	6,9605E-01	-0,10
Q3UDE2	TTL12	6,9617E-01	-0,25
P02463	CO4A1	6,9640E-01	0,03
Q9CQZ6	NDUB3	6,9651E-01	0,13
Q78HU3	MB12A	6,9651E-01	0,13
O70583	TRI18	6,9651E-01	0,13
Q8C7E7	STBD1	6,9651E-01	0,13
Q8CIN4	PAK2	6,9653E-01	-0,25
Q99LR1	ABD12	6,9688E-01	0,03
P35979	RL12	6,9698E-01	-0,05
P62071	RRAS2	6,9738E-01	0,02
Q08024	PEBB	6,9753E-01	0,03
P0DOV2	IFI4	6,9781E-01	-0,29
Q9WVA3	BUB3	6,9893E-01	-0,05
Q8R2R3	AAGAB	6,9908E-01	0,08
Q922H2	PDK3	6,9959E-01	0,08
Q80X85	RT07	6,9959E-01	0,08
Q8R238	SDSL	6,9959E-01	0,08
Q8K370	ACD10	7,0110E-01	-0,29
Q3TZZ7	ESYT2	7,0156E-01	-0,25
O70251	EF1B	7,0212E-01	-0,27
P30412	PPIC	7,0225E-01	0,01
Q60972	RBBP4	7,0286E-01	-0,05
O35955	PSB10	7,0299E-01	0,08
P56135	ATPK	7,0306E-01	0,03

P14869	RLA0	7,0310E-01	-0,09
Q60936	COQ8A	7,0349E-01	0,01
Q8C0D5	EFL1	7,0371E-01	-0,34
Q9WVP6	PAPOB	7,0417E-01	0,11
Q99LC2	CSTF1	7,0428E-01	0,00
Q9WVG6	CARM1	7,0432E-01	0,01
P49442	INPP	7,0489E-01	0,01
Q3UV17	K22O	7,0533E-01	-0,27
P61963	DCAF7	7,0554E-01	0,01
P62814	VATB2	7,0627E-01	-0,08
Q6PDM2	SRSF1	7,0640E-01	-0,23
P10107	ANXA1	7,0725E-01	-0,24
Q9ER41	TOR1B	7,0733E-01	0,08
Q9CRD2	EMC2	7,0763E-01	0,08
P28271	ACOC	7,0806E-01	-0,23
Q8K1R3	PNPT1	7,0844E-01	-0,04
O89017	LGMN	7,0860E-01	0,00
P28656	NP1L1	7,0907E-01	-0,25
P41216	ACSL1	7,0973E-01	-0,02
P70372	ELAV1	7,0978E-01	-0,06
Q9R0E2	PLOD1	7,0995E-01	-0,03
P46471	PRS7	7,1034E-01	-0,22
Q91VC3	IF4A3	7,1042E-01	-0,23
P08103	HCK	7,1056E-01	-0,27
Q3UVG3	F91A1	7,1104E-01	0,08
Q3UQ84	SYTM	7,1119E-01	0,10
Q8BGY7	F210A	7,1119E-01	0,10
P12265	BGLR	7,1121E-01	-0,01
O35639	ANXA3	7,1129E-01	-0,24
Q7TNV0	DEK	7,1138E-01	-0,02
Q8CBW3	ABI1	7,1204E-01	0,01
P47941	CRKL	7,1251E-01	0,00
P35700	PRDX1	7,1309E-01	-0,10
Q9QYF9	NDRG3	7,1342E-01	0,01
P23780	BGAL	7,1407E-01	-0,26
Q61107	GBP4	7,1413E-01	-0,28
Q64213	SF01	7,1443E-01	-0,02
Q8VC28	AK1CD	7,1471E-01	-0,25
P11031	TCP4	7,1578E-01	0,00
Q60854	SPB6	7,1647E-01	-0,11
Q9CQQ8	LSM7	7,1697E-01	-0,26
O35945	AL1A7	7,1757E-01	0,03
P05201	AATC	7,1768E-01	-0,10

Q5XJY5	COPD	7,1803E-01	-0,23
G5E829	AT2B1	7,1830E-01	-0,05
P56546	CTBP2	7,1886E-01	-0,27
Q9CPU4	MGST3	7,1910E-01	-0,26
P43025	TETN	7,1910E-01	-0,26
P56812	PDCD5	7,1910E-01	-0,26
O88907	PIAS1	7,1910E-01	-0,26
Q60809	CNOT7	7,1910E-01	-0,26
Q505D7	OPA3	7,1910E-01	-0,26
Q9WUR9	KAD4	7,1910E-01	-0,26
Q6P9Q4	FHOD1	7,1961E-01	-0,26
Q9Z0H4	CELF2	7,1967E-01	0,04
A2AX52	CO6A4	7,1968E-01	-0,13
P25799	NFKB1	7,1989E-01	0,04
P39429	TRAF2	7,2007E-01	-0,26
Q8K2H2	OTU6B	7,2007E-01	-0,26
Q9WVM3	APC7	7,2007E-01	-0,26
O70492	SNX3	7,2095E-01	0,04
P62141	PP1B	7,2138E-01	-0,07
Q9WVJ2	PSD13	7,2193E-01	-0,07
P62311	LSM3	7,2284E-01	0,04
P14094	AT1B1	7,2299E-01	-0,26
Q60872	IF1A	7,2327E-01	0,07
Q922J3	CLIP1	7,2335E-01	-0,26
Q91X51	GORS1	7,2357E-01	-0,26
Q9CRB6	TPPP3	7,2362E-01	0,04
Q61474	MSI1H	7,2362E-01	0,11
Q80X41	VRK1	7,2380E-01	-0,26
Q9CQV1	TIM16	7,2380E-01	-0,26
Q76MZ3	2AAA	7,2453E-01	-0,21
P97363	SPTC2	7,2498E-01	0,04
Q68FD5	CLH1	7,2536E-01	-0,20
Q9D7I5	LHPP	7,2549E-01	-0,33
Q8K1J6	TRNT1	7,2568E-01	-0,26
Q9CQ65	MTAP	7,2622E-01	-0,24
Q9JI48	PLAC8	7,2722E-01	-0,26
Q9CRC0	VKOR1	7,2722E-01	-0,26
Q99M28	RNPS1	7,2722E-01	-0,26
Q6DFX2	ANTR2	7,2722E-01	-0,26
O35658	C1QBP	7,2738E-01	-0,05
Q9CXY6	ILF2	7,2765E-01	-0,22
Q9QYI5	DNJB2	7,2777E-01	0,10
Q8R1G2	CMBL	7,2831E-01	-0,26

Q8R3E3	WIPI1	7,2831E-01	-0,26
Q8K4L3	SVIL	7,2831E-01	-0,26
P62835	RAP1A	7,2907E-01	0,00
O70378	EMC8	7,2990E-01	0,02
Q60749	KHDR1	7,2992E-01	0,00
Q9DBC3	CMTR1	7,3022E-01	0,02
Q8R3V5	SHLB2	7,3041E-01	-0,26
Q9Z2A5	ATE1	7,3041E-01	-0,26
Q9DBR3	ARMC8	7,3050E-01	0,04
Q9D287	SPF27	7,3135E-01	0,00
P53996	CNBP	7,3142E-01	-0,04
Q61581	IBP7	7,3209E-01	0,02
O08553	DPYL2	7,3214E-01	-0,11
O88448	KLC2	7,3298E-01	-0,24
Q99KJ8	DCTN2	7,3318E-01	-0,07
Q923G2	RPAB3	7,3326E-01	-0,26
P15327	PMGE	7,3363E-01	-0,26
P61021	RAB5B	7,3383E-01	-0,26
P67778	PHB	7,3387E-01	-0,09
Q9ERN0	SCAM2	7,3400E-01	0,02
Q64331	MYO6	7,3404E-01	-0,06
Q6PFR5	TRA2A	7,3455E-01	0,06
Q80U78	PUM1	7,3463E-01	-0,79
P35505	FAAA	7,3483E-01	0,04
Q9D0R8	LSM12	7,3665E-01	-0,26
P05063	ALDOC	7,3699E-01	0,10
Q91YP2	NEUL	7,3795E-01	-0,26
P61087	UBE2K	7,3799E-01	-0,26
Q9WUP4	PORED	7,3800E-01	0,10
Q9D1M7	FKB11	7,3800E-01	0,10
P11034	MCPT1	7,3800E-01	0,10
Q921N7	TMM70	7,3800E-01	0,10
Q99L20	GSTT3	7,3800E-01	0,10
Q9CXI0	COQ5	7,3800E-01	0,10
P63254	CRIP1	7,3800E-01	0,10
Q924T2	RT02	7,3800E-01	0,10
Q8R5A6	TB22A	7,3800E-01	0,10
O88522	NEMO	7,3800E-01	0,10
Q9CR29	CCD43	7,3800E-01	0,10
Q8BH60	GOPC	7,3800E-01	0,10
Q8R361	RFIP5	7,3800E-01	0,10
Q8VC30	TKFC	7,3800E-01	0,10
Q9WU00	NRF1	7,3800E-01	0,10

Q8VEA4	MIA40	7,3800E-01	0,10
Q6DID7	WLS	7,3800E-01	0,10
Q5SSM3	RHG44	7,3800E-01	0,10
P40630	TFAM	7,3800E-01	0,10
Q62036	CP131	7,3800E-01	0,10
Q91WR3	ASCC2	7,3800E-01	0,10
Q6GQS1	SCMC3	7,3800E-01	0,10
Q9Z2R6	U119A	7,3800E-01	0,10
Q9DCT5	SDF2	7,3800E-01	0,10
P62069	UBP46	7,3800E-01	0,10
P59016	VP33B	7,3800E-01	0,10
Q8BPB5	FBLN3	7,3820E-01	-0,25
Q9CZX8	RS19	7,3821E-01	-0,04
Q9CZP5	BCS1	7,3831E-01	0,06
Q6Q899	DDX58	7,3935E-01	-0,06
Q9CQY5	MAGT1	7,3946E-01	-0,26
Q3TIV5	ZC3HF	7,3956E-01	-0,26
P27612	PLAP	7,3978E-01	-0,23
Q922Y1	UBXN1	7,3985E-01	-0,03
Q8CFI0	NED4L	7,3999E-01	-0,27
Q9WUA2	SYFB	7,4031E-01	-0,25
P70349	HINT1	7,4055E-01	-0,26
Q64471	GSTT1	7,4128E-01	-0,26
Q8K010	OPLA	7,4200E-01	-0,24
Q8BMZ5	SEN34	7,4216E-01	-0,26
Q9QUR7	PIN1	7,4216E-01	-0,26
Q8C2Q3	RBM14	7,4234E-01	-0,08
P41105	RL28	7,4254E-01	0,06
Q78IK4	MIC27	7,4258E-01	-0,02
Q8CG50	RAB43	7,4260E-01	0,10
Q8BH58	TIPRL	7,4273E-01	-0,26
P35283	RAB12	7,4281E-01	0,10
Q9Z1P6	NDUA7	7,4325E-01	0,06
Q8VEH8	ERLEC	7,4340E-01	-0,26
Q6PAM1	TXLNA	7,4357E-01	-0,26
Q8K2A7	INT10	7,4387E-01	-0,26
Q9D1R9	RL34	7,4398E-01	0,06
O35381	AN32A	7,4529E-01	-0,07
P58022	LOXL2	7,4544E-01	-0,26
Q99LB7	SARDH	7,4569E-01	-0,26
Q6PDY2	AEDO	7,4571E-01	-0,26
P60824	CIRBP	7,4641E-01	-0,26
P24452	CAPG	7,4659E-01	-0,24

Q9ER72	SYCC	7,4663E-01	-0,03
Q9JL62	GLTP	7,4674E-01	-0,04
Q8R3I2	MBOA2	7,4697E-01	0,06
Q9CZE3	RAB32	7,4697E-01	0,06
Q80WR5	CA174	7,4697E-01	0,06
Q9WV98	TIM9	7,4697E-01	0,06
Q3TMH2	SCRN3	7,4697E-01	0,06
Q922B2	SYDC	7,4703E-01	-0,22
Q9CQW9	IFM3	7,4711E-01	0,00
Q93092	TALDO	7,4729E-01	-0,09
O35075	VP26C	7,4767E-01	-0,26
P51885	LUM	7,4799E-01	-0,24
P14106	C1QB	7,4847E-01	0,06
Q99P65	S29A3	7,4847E-01	0,06
Q60649	CLPB	7,4863E-01	-0,25
O55137	ACOT1	7,4867E-01	-0,25
Q6NZN0	RBM26	7,4926E-01	-0,26
O08529	CAN2	7,4940E-01	-0,10
Q6PGB8	SMCA1	7,4999E-01	-0,04
O35841	API5	7,5101E-01	-0,24
Q6P9Z1	SMRD3	7,5125E-01	0,08
P50543	S10AB	7,5236E-01	-0,22
P21107	TPM3	7,5369E-01	0,00
P14206	RSSA	7,5385E-01	-0,11
P17742	PPIA	7,5397E-01	-0,20
Q91WU5	AS3MT	7,5491E-01	-0,23
P63037	DNJA1	7,5553E-01	-0,07
O08915	AIP	7,5597E-01	-0,24
Q8VIJ6	SFPQ	7,5628E-01	-0,21
Q9EQK5	MVP	7,5655E-01	-0,22
Q80UY2	KCMF1	7,5716E-01	0,06
P63328	PP2BA	7,5850E-01	-0,04
Q8BGQ7	SYAC	7,5862E-01	-0,11
P51410	RL9	7,5936E-01	-0,07
P46061	RAGP1	7,5968E-01	-0,07
Q8CHH9	39692	7,5985E-01	-0,24
Q61390	TCPW	7,5987E-01	-0,25
Q9D0F3	LMAN1	7,6025E-01	-0,05
Q61466	SMRD1	7,6088E-01	-0,23
Q91WG2	RABE2	7,6099E-01	-0,19
Q8BT60	CPNE3	7,6101E-01	-0,23
Q7TT37	ELP1	7,6102E-01	-0,25
Q9D853	EFMT2	7,6144E-01	0,00

Q9Z0P5	TWF2	7,6146E-01	-0,25
Q9DCS9	NDUBA	7,6151E-01	-0,25
P62751	RL23A	7,6169E-01	-0,25
Q61239	FNTA	7,6282E-01	-0,05
Q99MR6	SRRT	7,6361E-01	-0,24
P60843	IF4A1	7,6397E-01	-0,12
Q9WTX6	CUL1	7,6406E-01	-0,24
P68040	RACK1	7,6406E-01	-0,11
P43135	COT2	7,6436E-01	-0,31
Q99M71	EPDR1	7,6439E-01	-0,07
P80318	TCPG	7,6513E-01	-0,12
Q9DBG3	AP2B1	7,6516E-01	-0,22
P80313	TCPH	7,6520E-01	-0,21
O09111	NDUBB	7,6527E-01	0,10
P07091	S10A4	7,6527E-01	0,10
Q80YV4	PANK4	7,6527E-01	0,10
Q8K0L9	ZBT20	7,6542E-01	-0,32
P04925	PRIO	7,6565E-01	0,10
Q80YD1	SUV3	7,6574E-01	0,00
Q8BU14	SEC62	7,6592E-01	0,02
Q8R1T1	CHMP7	7,6618E-01	-0,16
Q8VCG4	CO8G	7,6618E-01	-0,16
O55176	PJA1	7,6618E-01	-0,16
P97304	RPAC2	7,6618E-01	-0,16
Q9D771	PACC1	7,6618E-01	-0,16
Q91Z67	SRGP2	7,6618E-01	-0,16
Q5SYD0	MYO1D	7,6618E-01	-0,16
Q3ULA2	FBW1A	7,6618E-01	-0,16
Q99PP7	TRI33	7,6618E-01	-0,16
O54879	HMGB3	7,6618E-01	-0,16
O88653	LTOR3	7,6618E-01	-0,16
Q8BGT7	SPF30	7,6618E-01	-0,16
Q6P9R4	ARHGI	7,6618E-01	-0,16
Q3UMQ8	NAF1	7,6618E-01	-0,16
Q8K2C8	GPAT4	7,6618E-01	-0,16
P97820	M4K4	7,6618E-01	-0,16
Q91XE4	ACY3	7,6618E-01	-0,16
Q99J83	ATG5	7,6618E-01	-0,16
Q3UI43	BABA1	7,6618E-01	-0,16
P51912	AAAT	7,6618E-01	-0,16
Q99N96	RM01	7,6618E-01	-0,16
Q8K2D3	EDC3	7,6618E-01	-0,16
Q8CDJ8	STON1	7,6618E-01	-0,16

P61600	NAA20	7,6618E-01	-0,16
Q6P549	SHIP2	7,6618E-01	-0,16
Q9JJ59	ABCB9	7,6618E-01	-0,16
Q9QZZ4	MYO15	7,6618E-01	-0,16
O35892	SP100	7,6618E-01	-0,16
Q9JMG1	EDF1	7,6618E-01	-0,16
Q8CJ53	CIP4	7,6618E-01	-0,16
P53569	CEBPZ	7,6618E-01	-0,16
Q91YL3	UCKL1	7,6618E-01	-0,16
Q8VCI0	PLBL1	7,6618E-01	-0,16
Q91VJ5	PQBP1	7,6618E-01	-0,16
P55288	CAD11	7,6618E-01	-0,16
Q8BH86	GLUCM	7,6618E-01	-0,16
Q9JI90	RNF14	7,6618E-01	-0,16
O35683	NDUA1	7,6618E-01	-0,16
P61961	UFM1	7,6618E-01	-0,16
O88843	CRADD	7,6618E-01	-0,16
Q59J78	NDUF2	7,6618E-01	-0,16
Q9D7B7	GPX8	7,6618E-01	-0,16
Q9DB43	ZFPL1	7,6618E-01	-0,16
Q8VC57	KCTD5	7,6618E-01	-0,16
Q6KAR6	EXOC3	7,6618E-01	-0,16
P35456	UPAR	7,6618E-01	-0,16
Q8K245	UVRAG	7,6618E-01	-0,16
Q91XB7	YIF1A	7,6630E-01	0,10
Q3U2P1	SC24A	7,6643E-01	0,02
P19324	SERPH	7,6651E-01	-0,20
Q8BXZ1	TMX3	7,6668E-01	-0,04
Q64520	KGUA	7,6681E-01	-0,02
Q80WS3	FBLL1	7,6690E-01	0,07
P10404	ENV1	7,6693E-01	0,02
Q8BUK6	HOOK3	7,6722E-01	-0,24
Q9CQE1	NPS3B	7,6739E-01	0,00
Q8CJG1	AGO1	7,6743E-01	0,06
Q9R190	MTA2	7,6765E-01	-0,23
O08912	GALT1	7,6800E-01	0,06
Q5FWK3	RHG01	7,6818E-01	-0,23
Q8BYK6	YTHD3	7,6866E-01	-0,25
Q8BZQ7	ANC2	7,6867E-01	0,02
Q8VDP6	CDIPT	7,6884E-01	0,00
P84104	SRSF3	7,6921E-01	-0,24
Q60864	STIP1	7,6962E-01	-0,11
P59326	YTHD1	7,6999E-01	-0,18

Q9JIY5	HTRA2	7,7050E-01	-0,04
Q9R0B9	PLOD2	7,7057E-01	-0,08
Q9JK81	MYG1	7,7129E-01	-0,23
P54923	ADPRH	7,7174E-01	-0,23
P62855	RS26	7,7180E-01	-0,24
Q7TQH0	ATX2L	7,7207E-01	-0,03
Q9WUU7	CATZ	7,7256E-01	-0,23
Q62159	RHOC	7,7259E-01	0,03
P83940	ELOC	7,7268E-01	-0,03
Q9D0L7	ARM10	7,7275E-01	0,02
O55125	NIPS1	7,7304E-01	0,00
O35326	SRSF5	7,7322E-01	-0,22
P62717	RL18A	7,7334E-01	-0,03
Q9EPK6	SIL1	7,7371E-01	-0,02
Q6P9J9	ANO6	7,7377E-01	0,00
P24527	LKHA4	7,7382E-01	-0,12
Q8VCM8	NCLN	7,7429E-01	-0,07
O35317	PBX3	7,7432E-01	0,07
Q61545	EWS	7,7444E-01	-0,07
Q8CCK0	H2AW	7,7464E-01	-0,05
P15532	NDKA	7,7485E-01	-0,07
O35682	MYADM	7,7496E-01	-0,02
Q9WV85	NDK3	7,7504E-01	-0,03
Q9CQS8	SC61B	7,7535E-01	0,00
Q91WG4	ELP2	7,7553E-01	-0,02
P51125	ICAL	7,7561E-01	-0,24
Q8R2U0	SEH1	7,7576E-01	-0,02
Q9QYS9	QKI	7,7595E-01	-0,02
Q9JHW2	NIT2	7,7643E-01	-0,08
Q8BHZ0	FA49A	7,7652E-01	-0,24
Q60520	SIN3A	7,7773E-01	-0,42
Q3TDN2	FAF2	7,7904E-01	-0,23
P70362	UFD1	7,8045E-01	-0,02
Q69ZN7	MYOF	7,8065E-01	-0,15
P63001	RAC1	7,8121E-01	-0,24
Q9D4H1	EXOC2	7,8241E-01	0,00
P63168	DYL1	7,8247E-01	0,01
Q61543	GSLG1	7,8339E-01	-0,13
Q3TXS7	PSMD1	7,8417E-01	-0,12
Q64727	VINC	7,8424E-01	-0,19
Q9ES46	PARVB	7,8470E-01	0,02
Q80UU9	PGRC2	7,8492E-01	-0,24
P70452	STX4	7,8540E-01	0,03

P54116	STOM	7,8633E-01	-0,23
Q62093	SRSF2	7,8657E-01	-0,09
P40237	CD82	7,8664E-01	0,10
Q9QZQ8	H2AY	7,8678E-01	-0,11
Q9JI11	STK4	7,8681E-01	-0,23
Q3UKJ7	SMU1	7,8760E-01	-0,06
Q9CY58	PAIRB	7,8787E-01	-0,07
O35864	CSN5	7,8795E-01	-0,23
Q80SW1	SAHH2	7,8854E-01	-0,09
P23249	MOV10	7,8869E-01	-0,24
Q8K411	PREP	7,8902E-01	-0,21
Q9DCE5	PK1IP	7,8910E-01	0,00
Q8C854	MYEF2	7,8984E-01	-0,06
Q8CIE6	COPA	7,9067E-01	-0,24
Q9Z0M5	LICH	7,9169E-01	-0,42
P49817	CAV1	7,9203E-01	-0,08
O55142	RL35A	7,9222E-01	0,03
Q08481	PECA1	7,9222E-01	0,03
Q9QUR8	SEM7A	7,9222E-01	0,03
Q6DVA0	LEMD2	7,9222E-01	0,03
Q9CPS7	PNO1	7,9275E-01	0,10
O35130	NEP1	7,9288E-01	-0,26
P70271	PDLI4	7,9298E-01	-0,02
Q9Z0G0	GIPC1	7,9335E-01	0,06
P18242	CATD	7,9336E-01	-0,11
Q08943	SSRP1	7,9414E-01	-0,22
P52623	UCK1	7,9580E-01	-0,23
Q8VEB4	PAG15	7,9606E-01	-0,22
Q3UVK0	ERMP1	7,9631E-01	-0,06
Q91W34	RUS1	7,9658E-01	0,03
P08101	FCGR2	7,9658E-01	0,03
P15208	INSR	7,9658E-01	0,03
Q8C0Y0	PP4R4	7,9658E-01	0,03
Q61584	FXR1	7,9676E-01	-0,08
Q61103	REQU	7,9754E-01	-0,23
Q8VE80	THOC3	7,9768E-01	0,03
Q7TMY4	THOC7	7,9768E-01	0,03
Q8BGX0	TRI23	7,9768E-01	0,03
Q91YQ5	RPN1	7,9833E-01	-0,12
P18760	COF1	7,9860E-01	-0,12
P70297	STAM1	7,9899E-01	-0,23
Q9R059	FHL3	7,9967E-01	-0,23
P70670	NACAM	8,0029E-01	-0,09

Q9EP69	SAC1	8,0118E-01	-0,09
P27773	PDIA3	8,0123E-01	-0,13
Q9CYL5	GAPR1	8,0145E-01	-0,22
Q91VJ2	CAVN3	8,0212E-01	-0,23
Q91VZ6	SMAP1	8,0226E-01	0,03
O55128	SAP18	8,0226E-01	0,03
Q8BH57	WDR48	8,0226E-01	0,03
Q9DAA6	EXOS1	8,0226E-01	0,03
O54786	DFFA	8,0226E-01	0,03
P63005	LIS1	8,0241E-01	-0,21
Q3UCV8	OTUL	8,0314E-01	0,10
Q9CQR6	PPP6	8,0341E-01	-0,23
Q8BH43	WASF2	8,0350E-01	-0,23
Q3V009	TMED1	8,0350E-01	-0,23
O35405	PLD3	8,0350E-01	-0,23
Q9D1R2	KTI12	8,0354E-01	-0,19
C0HKG5	RNT2A	8,0354E-01	-0,19
Q9ER73	ELP4	8,0354E-01	-0,19
O55057	PDE6D	8,0354E-01	-0,19
A2BIM8	MUP18	8,0354E-01	-0,19
Q9CQS2	NOP10	8,0360E-01	-0,42
Q9CWS4	INT11	8,0367E-01	0,06
Q9WUM3	COR1B	8,0406E-01	-0,22
Q62148	AL1A2	8,0411E-01	-0,22
Q61249	IGBP1	8,0443E-01	-0,22
Q99LI8	HGS	8,0447E-01	-0,23
Q61554	FBN1	8,0555E-01	-0,08
Q8R086	SUOX	8,0594E-01	-0,23
P63085	MK01	8,0710E-01	-0,22
P67871	CSK2B	8,0717E-01	-0,23
Q6ZWQ7	SPCS3	8,0736E-01	-0,23
O55229	CHKB	8,0739E-01	-0,22
Q8JZV7	NAGA	8,0757E-01	-0,23
P51859	HDGF	8,0789E-01	-0,04
Q5XG69	F169A	8,0803E-01	0,00
O08585	CLCA	8,0850E-01	-0,04
Q8JZU2	TXTP	8,0853E-01	-0,23
Q9Z110	P5CS	8,0856E-01	-0,10
Q8BHN3	GANAB	8,0871E-01	-0,12
Q9Z108	STAU1	8,0884E-01	0,00
P39061	COIA1	8,0884E-01	0,00
Q9Z1K5	ARI1	8,0898E-01	-0,23
Q62417	SRBS1	8,0907E-01	-0,06

Q3TQB2	FXRD1	8,0911E-01	0,03
Q9JKW0	AR6P1	8,0911E-01	0,03
Q8K2T8	PAF1	8,0925E-01	-0,04
Q99JR8	SMRD2	8,0967E-01	-0,05
Q9CQC7	NDUB4	8,1000E-01	0,03
Q9D8N2	DEN10	8,1000E-01	0,03
C0HK79	ARXS1	8,1031E-01	-0,22
P0DOV1	IFI5B	8,1033E-01	-0,04
O08539	BIN1	8,1085E-01	-0,04
Q91Z53	GRHPR	8,1103E-01	-0,21
Q9ESB3	HRG	8,1110E-01	-0,04
Q3UYH7	ARBK2	8,1147E-01	-0,19
Q9DB27	MCTS1	8,1147E-01	-0,19
Q3UL36	ARGL1	8,1147E-01	-0,19
P12246	SAMP	8,1147E-01	-0,19
Q9CR67	TMM33	8,1147E-01	-0,19
Q78PG9	CCD25	8,1147E-01	-0,19
Q60605	MYL6	8,1150E-01	-0,10
Q9CQF4	MRES1	8,1152E-01	-0,21
O08795	GLU2B	8,1153E-01	-0,10
Q58A65	JIP4	8,1157E-01	0,00
P62858	RS28	8,1202E-01	-0,03
Q8VEK3	HNRPU	8,1239E-01	-0,20
Q9CYI4	LUC7L	8,1262E-01	-0,22
Q3UBX0	TM109	8,1270E-01	-0,22
Q9R008	KIME	8,1270E-01	0,00
Q80VQ1	LRRC1	8,1281E-01	-0,19
Q9D483	RPC3	8,1302E-01	0,06
Q91Z96	BMP2K	8,1302E-01	0,06
Q99N94	RM09	8,1302E-01	0,06
Q6ZWV7	RL35	8,1302E-01	0,06
P10810	CD14	8,1302E-01	0,06
O35144	TERF2	8,1302E-01	0,06
Q8C0E3	TRI47	8,1302E-01	0,06
Q9D2R6	COA3	8,1302E-01	0,06
Q60790	RASA3	8,1302E-01	0,06
Q8VDP2	CX056	8,1302E-01	0,06
Q6GQT6	SCAP	8,1302E-01	0,06
Q69ZF3	GBA2	8,1302E-01	0,06
Q99KK1	REEP3	8,1302E-01	0,06
Q80Y86	MK15	8,1302E-01	0,06
Q9DBF7	CWC25	8,1302E-01	0,06
P70451	FER	8,1302E-01	0,06

Q8C3F2	F120C	8,1302E-01	0,06
Q8WTY4	CPIN1	8,1302E-01	0,06
Q9JL15	LEG8	8,1302E-01	0,06
Q9D168	INT12	8,1302E-01	0,06
Q9QXD8	LIMD1	8,1302E-01	0,06
O35071	KIF1C	8,1302E-01	0,06
Q8BTT6	DIEXF	8,1302E-01	0,06
P28076	PSB9	8,1302E-01	0,06
Q3USZ8	DIK2A	8,1302E-01	0,06
Q8QZY6	TSN14	8,1302E-01	0,06
Q9CRA0	NAR4	8,1302E-01	0,06
Q00993	UFO	8,1302E-01	0,06
Q8R3I3	COG6	8,1302E-01	0,06
Q9Z280	PLD1	8,1302E-01	0,06
Q3UQN2	FCHO2	8,1302E-01	0,06
Q9D7P9	SPB12	8,1302E-01	0,06
P97503	NKX32	8,1302E-01	0,06
P60762	MO4L1	8,1302E-01	0,06
Q60772	CDN2C	8,1302E-01	0,06
Q91VR7	MLP3A	8,1302E-01	0,06
Q9CXL3	CG050	8,1302E-01	0,06
Q80YR4	ZN598	8,1302E-01	0,06
P62342	SELT	8,1302E-01	0,06
Q91YU8	SSF1	8,1302E-01	0,06
Q7TMS5	ABCG2	8,1302E-01	0,06
Q9JIK9	RT34	8,1302E-01	0,06
P98083	SHC1	8,1302E-01	0,06
Q9JI99	SGPP1	8,1302E-01	0,06
Q91YT8	CSCL1	8,1302E-01	0,06
Q99K43	PRC1	8,1302E-01	0,06
Q9D8H7	OMA1	8,1302E-01	0,06
O89106	FHIT	8,1302E-01	0,06
Q61165	SL9A1	8,1302E-01	0,06
Q8C5P7	TDRP	8,1302E-01	0,06
Q8BL86	MBLC2	8,1302E-01	0,06
Q6PCM2	INT6	8,1302E-01	0,06
Q9D2Y4	MLKL	8,1302E-01	0,06
Q4VAA2	CDV3	8,1302E-01	0,06
Q80YA7	DPP8	8,1302E-01	0,06
A2A690	TANC2	8,1302E-01	0,06
Q921L5	COG2	8,1302E-01	0,06
Q8K2Q9	SHOT1	8,1302E-01	0,06
Q91XV3	BASP1	8,1302E-01	0,06

Q08639	TFDP1	8,1302E-01	0,06
Q3U1D0	LINES	8,1302E-01	0,06
Q9QXN3	TRIP4	8,1302E-01	0,06
Q9Z1R3	APOM	8,1302E-01	0,06
P35288	RAB23	8,1302E-01	0,06
Q9D1G5	LRC57	8,1302E-01	0,06
P16054	KPCE	8,1302E-01	0,06
Q60876	4EBP1	8,1302E-01	0,06
P97367	MEIS2	8,1302E-01	0,06
P70274	SEPP1	8,1302E-01	0,06
Q8BWJ3	KPB2	8,1302E-01	0,06
P18826	KPB1	8,1302E-01	0,06
Q9CYA6	ZCHC8	8,1323E-01	-0,21
P52332	JAK1	8,1323E-01	-0,22
P62827	RAN	8,1354E-01	-0,09
Q9CQM5	TXD17	8,1373E-01	-0,03
Q6GV12	KDSR	8,1421E-01	0,00
Q91VW3	SH3L3	8,1450E-01	-0,03
P30285	CDK4	8,1488E-01	-0,21
O35900	LSM2	8,1522E-01	-0,02
Q8BXN9	TM87A	8,1534E-01	-0,42
Q9D8E6	RL4	8,1537E-01	-0,09
Q9ER00	STX12	8,1566E-01	-0,02
Q9DCJ5	NDUA8	8,1566E-01	-0,02
Q9CQC8	SPG21	8,1566E-01	-0,02
Q9DBC0	SELO	8,1636E-01	-0,23
Q8K386	RAB15	8,1654E-01	-0,03
Q8BHG9	CGBP1	8,1662E-01	-0,19
P52760	RIDA	8,1662E-01	-0,19
Q9DBS9	OSBL3	8,1662E-01	-0,19
O08600	NUCG	8,1662E-01	-0,19
Q8K3A9	MEPCE	8,1662E-01	-0,19
Q8K358	PIGU	8,1662E-01	-0,19
P36916	GNL1	8,1662E-01	-0,19
Q80U72	SCRIB	8,1668E-01	-0,19
Q8CEE7	RDH13	8,1700E-01	0,03
P61979	HNRPK	8,1702E-01	-0,19
P63325	RS10	8,1724E-01	-0,02
Q3UHX2	HAP28	8,1742E-01	-0,22
P60904	DNJC5	8,1742E-01	-0,22
Q8VE38	OXND1	8,1742E-01	0,32
Q9CQV4	RETR3	8,1773E-01	-0,02
O88738	BIRC6	8,1784E-01	-0,02

Q8C0E2	VP26B	8,1788E-01	-0,07
Q9WV70	NOC2L	8,1830E-01	0,00
Q9JII5	DAZP1	8,1889E-01	-0,07
Q61411	RASH	8,1891E-01	-0,03
Q9DBT5	AMPD2	8,1899E-01	-0,19
Q63932	MP2K2	8,1899E-01	-0,21
Q9CWG8	NDUF7	8,1927E-01	-0,21
Q9QYB1	CLIC4	8,2016E-01	-0,21
O54774	AP3D1	8,2071E-01	-0,07
P62075	TIM13	8,2072E-01	-0,23
P61971	NTF2	8,2115E-01	-0,22
P47963	RL13	8,2142E-01	-0,22
Q64008	RAB34	8,2159E-01	-0,21
Q9D0R2	SYTC	8,2169E-01	-0,11
Q9CSU0	RPR1B	8,2256E-01	-0,22
P84099	RL19	8,2336E-01	0,00
P27601	GNA13	8,2386E-01	-0,07
P29391	FRIL1	8,2424E-01	-0,21
P61982	1433G	8,2430E-01	-0,11
P47911	RL6	8,2438E-01	-0,26
Q9JHL1	NHRF2	8,2513E-01	-0,07
Q9JMH9	MY18A	8,2547E-01	-0,38
O54890	ITB3	8,2555E-01	-0,08
Q3TZX8	NOL9	8,2557E-01	-0,21
Q8R3F5	FABD	8,2576E-01	-0,22
Q6P8I4	PCNP	8,2576E-01	-0,22
P62889	RL30	8,2587E-01	-0,08
P84244	H33	8,2592E-01	-0,22
Q9QXE0	HACL1	8,2593E-01	-0,22
Q3UGC7	EI3JA	8,2616E-01	-0,07
P13020	GELS	8,2650E-01	-0,20
O88696	CLPP	8,2676E-01	-0,07
Q9JJY4	DDX20	8,2680E-01	-0,21
P62334	PRS10	8,2723E-01	-0,12
Q6URW6	MYH14	8,2746E-01	-0,01
Q99020	ROAA	8,2770E-01	-0,20
P57780	ACTN4	8,2832E-01	-0,14
Q91VN4	MIC25	8,2856E-01	0,00
Q60973	RBBP7	8,2869E-01	-0,09
Q9Z1T1	AP3B1	8,2968E-01	-0,08
Q8CI59	STEA3	8,2999E-01	-0,22
Q8VDD5	MYH9	8,3018E-01	-0,19
P10518	HEM2	8,3041E-01	-0,09

Q5U4D9	THOC6	8,3111E-01	-0,07
Q9Z172	SUMO3	8,3201E-01	-0,16
O88939	ZBT7A	8,3216E-01	-0,19
Q3TFD2	PCAT1	8,3216E-01	-0,19
Q8CGF7	TCRG1	8,3316E-01	-0,22
Q80VI1	TRI56	8,3346E-01	-0,21
Q61656	DDX5	8,3363E-01	-0,12
P03958	ADA	8,3374E-01	-0,21
Q791V5	MTCH2	8,3381E-01	-0,21
P22315	HEMH	8,3451E-01	-0,07
Q9EPJ9	ARFG1	8,3459E-01	-0,21
Q9CXF4	TBC15	8,3486E-01	-0,22
P06745	G6PI	8,3610E-01	-0,11
P57716	NICA	8,3682E-01	-0,21
Q8K4I3	ARHG6	8,3720E-01	-0,07
Q9CWK8	SNX2	8,3785E-01	-0,20
Q99JB2	STML2	8,3895E-01	-0,10
P35831	PTN12	8,3910E-01	-0,19
P27659	RL3	8,3929E-01	-0,12
Q9CX56	PSMD8	8,4032E-01	-0,21
P09528	FRIH	8,4040E-01	-0,21
Q8CFV9	RIFK	8,4042E-01	-0,19
Q9CRB2	NHP2	8,4046E-01	-0,21
Q8K0U4	HS12A	8,4078E-01	-0,20
P97384	ANX11	8,4194E-01	-0,11
Q8BH97	RCN3	8,4224E-01	-0,20
Q9DBS5	KLC4	8,4243E-01	-0,21
Q3TIX9	SNUT2	8,4268E-01	-0,06
Q9EPR4	S23A2	8,4285E-01	-0,09
Q99JI6	RAP1B	8,4292E-01	-0,09
P63323	RS12	8,4315E-01	-0,21
P97821	CATC	8,4384E-01	-0,21
Q63829	COMD3	8,4388E-01	-0,09
Q91VI7	RINI	8,4391E-01	-0,19
P35821	PTN1	8,4397E-01	-0,21
P46664	PURA2	8,4409E-01	-0,21
Q6P8X1	SNX6	8,4500E-01	-0,20
Q61941	NNTM	8,4598E-01	-0,12
Q62376	RU17	8,4654E-01	-0,21
Q9CYR0	SSBP	8,4669E-01	-0,21
P47809	MP2K4	8,4758E-01	-0,21
P60229	EIF3E	8,4774E-01	-0,12
Q9JKX6	NUDT5	8,4775E-01	-0,06

Q9D8W5	PSD12	8,4778E-01	-0,12
P61202	CSN2	8,4795E-01	-0,20
Q68FL4	SAHH3	8,4809E-01	-0,20
O88569	ROA2	8,4848E-01	-0,14
Q80TV8	CLAP1	8,4865E-01	0,02
P07214	SPRC	8,4885E-01	-0,07
P97461	RS5	8,5015E-01	-0,11
O08759	UBE3A	8,5076E-01	-0,20
Q8BVQ5	PPME1	8,5170E-01	-0,21
Q91VK1	BZW2	8,5303E-01	-0,20
P35980	RL18	8,5336E-01	-0,05
P62754	RS6	8,5353E-01	-0,09
Q9CPQ3	TOM22	8,5401E-01	-0,09
Q9CWZ3	RBM8A	8,5418E-01	-0,05
Q3TL44	NLRX1	8,5454E-01	0,00
Q9D7A8	ARMC1	8,5660E-01	-0,06
Q9CRA5	GOLP3	8,5666E-01	-0,20
Q8BH64	EHD2	8,5729E-01	-0,20
O54865	GCYB1	8,5750E-01	-0,08
Q91VH6	MEMO1	8,5781E-01	-0,20
Q3TWW8	SRSF6	8,5782E-01	-0,20
Q9D8X1	CUTC	8,5806E-01	-0,19
Q64378	FKBP5	8,5808E-01	-0,19
Q6ZPR5	NSMA3	8,5814E-01	-0,19
Q60668	HNRPD	8,5879E-01	-0,19
P63024	VAMP3	8,5887E-01	-0,20
Q9Z0H3	SNF5	8,5892E-01	-0,08
Q8R332	NUP58	8,5893E-01	-0,03
Q8BX10	PGAM5	8,5970E-01	-0,04
Q8CH72	TRI32	8,5970E-01	-0,04
Q8CGN5	PLIN1	8,5981E-01	-0,10
Q8R0H9	GGA1	8,5998E-01	0,00
P97372	PSME2	8,6075E-01	-0,09
Q64105	SPRE	8,6132E-01	-0,20
Q9CZY3	UB2V1	8,6134E-01	-0,20
Q8CG72	ARHL2	8,6185E-01	-0,20
Q8BYY4	TT39B	8,6196E-01	-0,03
P14069	S10A6	8,6196E-01	-0,03
Q8BTW3	EXOS6	8,6203E-01	-0,04
P52430	PON1	8,6258E-01	-0,04
A2ASQ1	AGRIN	8,6338E-01	-0,02
Q9QZE7	TSNAX	8,6344E-01	-0,08
Q5SSZ5	TENS3	8,6349E-01	-0,17

Q91VA6	PDIP2	8,6445E-01	-0,21
P48774	GSTM5	8,6471E-01	-0,10
Q8BY89	CTL2	8,6482E-01	-0,19
P0C0S6	H2AZ	8,6498E-01	-0,18
P16110	LEG3	8,6509E-01	-0,08
Q8R2Y8	PTH2	8,6530E-01	-0,08
P63260	ACTG	8,6637E-01	-0,18
P61028	RAB8B	8,6639E-01	-0,03
Q9R0Q7	TEBP	8,6648E-01	-0,20
P11438	LAMP1	8,6701E-01	-0,08
Q8BXQ2	PIGT	8,6733E-01	-0,04
Q6P1F6	2ABA	8,6735E-01	-0,10
Q08091	CNN1	8,6740E-01	-0,20
Q99JF5	MVD1	8,6746E-01	-0,03
P16254	SRP14	8,6754E-01	0,00
P0C0A3	CHMP6	8,6754E-01	0,00
Q9CQ88	TSN31	8,6754E-01	0,00
Q9QXA5	LSM4	8,6754E-01	0,00
Q9CQ40	RM49	8,6754E-01	0,00
P42208	37500	8,6766E-01	-0,12
Q9EQG9	CERT	8,6782E-01	-0,19
Q6NSR8	PEPL1	8,6798E-01	-0,20
Q80X73	PELO	8,6858E-01	-0,19
Q922P9	GLYR1	8,6879E-01	-0,10
Q8CH18	CCAR1	8,6887E-01	-0,20
P01887	B2MG	8,6931E-01	-0,19
Q9D824	FIP1	8,6931E-01	-0,19
Q61235	SNTB2	8,6940E-01	-0,20
Q566J8	COQ8B	8,6947E-01	0,00
Q9D1B9	RM28	8,6947E-01	0,00
A8C756	THADA	8,6947E-01	0,00
Q9D6U8	F162A	8,6947E-01	0,00
Q8R317	UBQL1	8,6990E-01	-0,20
Q9CX34	SGT1	8,7004E-01	-0,11
Q99LM2	CK5P3	8,7026E-01	-0,20
Q9Z1G3	VATC1	8,7081E-01	-0,19
Q61187	TS101	8,7081E-01	-0,19
Q9CX86	ROA0	8,7108E-01	-0,11
Q8R3Y8	I2BP1	8,7144E-01	0,00
Q9CQ54	NDUC2	8,7148E-01	-0,03
Q6DFW4	NOP58	8,7159E-01	-0,19
Q61686	CBX5	8,7204E-01	-0,08
Q03963	E2AK2	8,7217E-01	-0,19

Q9R0X4	ACOT9	8,7226E-01	-0,10
P62843	RS15	8,7254E-01	-0,03
Q3URE1	ACSF3	8,7254E-01	-0,03
Q9D1P4	CHRD1	8,7258E-01	-0,20
Q3TDQ1	STT3B	8,7271E-01	-0,08
Q9R0P4	SMAP	8,7318E-01	-0,03
Q9WV03	FA50A	8,7335E-01	-0,03
Q91WK0	LRRF2	8,7352E-01	-0,17
Q9QYH6	MAGD1	8,7408E-01	-0,17
Q9CQ60	6PGL	8,7424E-01	-0,19
Q80VP1	EPN1	8,7451E-01	-0,18
O08756	HCD2	8,7572E-01	-0,19
Q9CQC6	BZW1	8,7574E-01	-0,19
Q9Z1F9	SAE2	8,7647E-01	-0,12
Q921K8	TCAF2	8,7665E-01	-0,04
Q8R1V4	TMED4	8,7670E-01	-0,19
P62852	RS25	8,7681E-01	-0,08
Q91XU3	PI42C	8,7704E-01	0,00
Q6ZWN5	RS9	8,7707E-01	-0,19
Q80W03	TOX3	8,7735E-01	-0,04
Q3TLP5	ECHD2	8,7759E-01	-0,18
Q9DC28	KC1D	8,7785E-01	-0,14
Q6PGL7	WASC2	8,7802E-01	-0,04
Q61164	CTCF	8,7825E-01	-0,19
Q91YJ3	THYN1	8,7912E-01	0,00
Q8CDN6	TXNL1	8,7942E-01	-0,19
Q8BGE6	ATG4B	8,8049E-01	-0,19
Q8BHE8	MAIP1	8,8054E-01	-0,03
Q3TCJ1	ABRX2	8,8077E-01	-0,18
Q3UEB3	PUF60	8,8093E-01	-0,19
Q04750	TOP1	8,8120E-01	-0,19
Q64429	CP1B1	8,8123E-01	-0,19
P12804	FGL2	8,8186E-01	-0,09
O88342	WDR1	8,8200E-01	-0,13
Q91VM5	RMXL1	8,8220E-01	-0,12
Q91W89	MA2C1	8,8271E-01	-0,18
Q8BPB0	MOB1B	8,8289E-01	-0,18
Q8C0M9	ASGL1	8,8303E-01	-0,18
Q04207	TF65	8,8351E-01	-0,07
P97470	PP4C	8,8391E-01	-0,09
Q8BRF7	SCFD1	8,8492E-01	-0,11
Q8CHS8	VP37A	8,8496E-01	-0,18
P14231	AT1B2	8,8496E-01	-0,18

Q3UZ39	LRRF1	8,8543E-01	0,01
Q60766	IRGM1	8,8572E-01	-0,07
Q9ERI6	RDH14	8,8638E-01	-0,07
Q80UG5	40057	8,8672E-01	-0,12
Q9Z2D8	MBD3	8,8732E-01	-0,16
Q8C7K6	PCYXL	8,8733E-01	-0,16
P08207	S10AA	8,8758E-01	-0,16
Q6ZWM4	LSM8	8,8829E-01	-0,07
Q31125	S39A7	8,8906E-01	-0,18
Q9DBH5	LMAN2	8,8922E-01	-0,11
P62849	RS24	8,8977E-01	-0,09
O35685	NUDC	8,8987E-01	-0,11
Q9EQH2	ERAP1	8,9077E-01	-0,11
Q6PDQ2	CHD4	8,9079E-01	-0,23
O88485	DC1I1	8,9121E-01	-0,18
Q8VE47	UBA5	8,9145E-01	-0,09
P16460	ASSY	8,9149E-01	-0,07
Q8BH61	F13A	8,9192E-01	-0,07
Q8R121	ZPI	8,9230E-01	-0,14
Q921X9	PDIA5	8,9233E-01	-0,19
P20444	KPCA	8,9240E-01	-0,04
Q9D5V6	SYAP1	8,9307E-01	-0,10
Q9D0F9	PGM1	8,9342E-01	-0,19
Q6PA06	ATLA2	8,9359E-01	-0,19
P23591	FCL	8,9376E-01	-0,19
P47934	CACP	8,9377E-01	-0,16
P18528	HVM57	8,9386E-01	-0,07
P63158	HMGB1	8,9386E-01	-0,19
Q05793	PGBM	8,9472E-01	-0,22
P17156	HSP72	8,9481E-01	-0,09
Q8BPG6	SUMF2	8,9555E-01	-0,07
Q3TUH1	TAM41	8,9558E-01	-0,16
Q9EQQ9	OGA	8,9573E-01	-0,33
P70671	IRF3	8,9573E-01	-0,33
O35286	DHX15	8,9613E-01	-0,18
P97822	AN32E	8,9614E-01	-0,19
P62500	T22D1	8,9703E-01	-0,14
Q9D1L0	CHCH2	8,9780E-01	-0,19
P62878	RBX1	8,9796E-01	-0,09
Q6PGH1	BUD31	8,9810E-01	-0,21
P01630	KV2A6	8,9835E-01	-0,14
Q9JLV5	CUL3	8,9876E-01	-0,19
P46414	CDN1B	8,9878E-01	-0,19

Q91WM1	STRBP	8,9901E-01	-0,06
P52633	STAT6	8,9907E-01	-0,04
Q8K1E0	STX5	8,9953E-01	-0,07
Q99K51	PLST	8,9979E-01	-0,12
Q8BSY0	ASPH	9,0010E-01	-0,12
Q61160	FADD	9,0039E-01	-0,16
O35691	PININ	9,0039E-01	-0,16
Q6R891	NEB2	9,0049E-01	-0,07
Q8BL66	EEA1	9,0068E-01	-0,20
P26516	PSMD7	9,0162E-01	-0,12
Q9ES74	NEK7	9,0174E-01	-0,16
Q8BJY1	PSMD5	9,0180E-01	-0,13
O89051	ITM2B	9,0194E-01	-0,16
Q9JLT4	TRXR2	9,0194E-01	-0,18
P11499	HS90B	9,0200E-01	-0,17
Q8K023	AKC1H	9,0222E-01	-0,38
Q8QZY1	EIF3L	9,0249E-01	-0,13
P70333	HNRH2	9,0291E-01	-0,18
A2AGT5	CKAP5	9,0357E-01	-0,16
Q80VD1	FA98B	9,0417E-01	-0,12
Q9CQC9	SAR1B	9,0424E-01	-0,09
Q4PJX1	ODR4	9,0437E-01	-0,07
Q8VBV7	CSN8	9,0452E-01	-0,07
P28741	KIF3A	9,0468E-01	0,03
Q7TMB8	CYFP1	9,0629E-01	-0,17
Q64735	CR1L	9,0642E-01	-0,07
P43276	H15	9,0667E-01	-0,12
P09242	PPBT	9,0708E-01	-0,18
Q9WV91	FPRP	9,0710E-01	-0,14
Q8BV13	CSN7B	9,0710E-01	-0,14
Q91YT0	NDUV1	9,0803E-01	-0,12
Q61151	2A5E	9,0821E-01	-0,17
Q99J09	MEP50	9,0864E-01	-0,18
Q9CR00	PSMD9	9,0880E-01	-0,18
Q61081	CDC37	9,0906E-01	-0,18
Q03347	RUNX1	9,0942E-01	0,20
Q9JJ00	PLS1	9,0994E-01	-0,07
Q8BGB5	LIMD2	9,1000E-01	-0,07
Q9Z2D1	MTMR2	9,1020E-01	-0,14
P50295	ARY2	9,1020E-01	-0,14
P63330	PP2AA	9,1051E-01	-0,11
D3YXK2	SAFB1	9,1068E-01	-0,10
Q9ERB0	SNP29	9,1069E-01	-0,07

Q99KW3	TARA	9,1069E-01	-0,07
P63321	RALA	9,1144E-01	-0,18
P53986	MOT1	9,1203E-01	-0,07
Q8K1Z0	COQ9	9,1203E-01	-0,07
Q05BC3	EMAL1	9,1214E-01	-0,14
Q9CWU9	NUP37	9,1221E-01	-0,18
P84089	ERH	9,1267E-01	-0,11
Q9QZQ1	AFAD	9,1277E-01	-0,18
O70274	TP4A2	9,1278E-01	-0,07
Q9WTK3	GPAA1	9,1321E-01	-0,09
Q6P6L0	FIL1L	9,1321E-01	-0,09
Q9CPQ1	COX6C	9,1341E-01	-0,07
Q8BK63	KC1A	9,1396E-01	-0,18
Q8BZF8	PGM5	9,1414E-01	-0,12
P50247	SAHH	9,1461E-01	-0,17
Q9JHS4	CLPX	9,1475E-01	-0,11
Q501J6	DDX17	9,1506E-01	-0,18
Q91Z83	MYH7	9,1530E-01	-0,14
Q9CXJ4	ABCB8	9,1534E-01	-0,09
Q8R146	APEH	9,1576E-01	-0,13
Q925I1	ATAD3	9,1577E-01	-0,12
P50580	PA2G4	9,1578E-01	-0,14
Q61398	PCOC1	9,1581E-01	-0,16
P10639	THIO	9,1594E-01	-0,09
Q9D404	OXSM	9,1621E-01	-0,07
O55028	BCKD	9,1628E-01	-0,14
Q99K41	EMIL1	9,1628E-01	-0,13
P23506	PIMT	9,1665E-01	-0,17
Q8N9S3	AHSA2	9,1739E-01	-0,06
Q99KK2	NEUA	9,1765E-01	-0,09
Q8BTV2	CPSF7	9,1809E-01	-0,09
A2APV2	FMNL2	9,1824E-01	-0,04
Q9D020	5NT3A	9,1830E-01	-0,07
Q9CR61	NDUB7	9,1840E-01	-0,07
Q8CBE3	WDR37	9,1841E-01	-0,06
P49586	PCY1A	9,1864E-01	-0,13
Q8BFY9	TNPO1	9,1870E-01	-0,13
Q3UPF5	ZCCHV	9,1886E-01	-0,09
O88545	CSN6	9,1894E-01	-0,18
Q80TH2	ERBIN	9,1908E-01	-0,14
Q9CQ22	LTOR1	9,1925E-01	-0,14
P62322	LSM5	9,1925E-01	-0,14
Q8K0V4	CNOT3	9,1925E-01	-0,14

Q9CQ75	NDUA2	9,1925E-01	-0,14
O88630	GOSR1	9,1925E-01	-0,14
Q922E6	FAKD2	9,1957E-01	-0,16
O88502	PDE8A	9,1957E-01	-0,16
Q8BP67	RL24	9,1967E-01	-0,18
Q9QXS1	PLEC	9,1968E-01	-0,20
O70493	SNX12	9,2034E-01	-0,07
Q91WC0	SETD3	9,2049E-01	-0,17
P62743	AP2S1	9,2062E-01	0,00
P31750	AKT1	9,2143E-01	-0,18
O35344	IMA4	9,2153E-01	-0,18
Q4LDD4	ARAP1	9,2194E-01	-0,15
Q9Z2L7	CRLF3	9,2207E-01	-0,14
O54950	AAKG1	9,2244E-01	-0,09
Q62419	SH3G1	9,2291E-01	-0,17
P63073	IF4E	9,2293E-01	-0,11
O35345	IMA7	9,2315E-01	-0,17
O08715	AKAP1	9,2377E-01	-0,16
Q9ES56	TPPC4	9,2377E-01	-0,16
P62196	PRS8	9,2401E-01	-0,18
Q9QYC0	ADDA	9,2412E-01	-0,18
P05977	MYL1	9,2457E-01	-0,16
Q9JJZ4	UB2J1	9,2463E-01	-0,06
Q78XF5	OSTC	9,2479E-01	-0,06
Q9D6T0	NOSIP	9,2479E-01	-0,06
Q61739	ITA6	9,2485E-01	-0,17
Q60865	CAPR1	9,2490E-01	-0,18
Q8R1F1	NIBA2	9,2549E-01	-0,09
O70209	PDLI3	9,2623E-01	-0,09
Q8BZW8	NHLC2	9,2674E-01	-0,16
Q5F2E7	NUFP2	9,2732E-01	-0,06
O88543	CSN3	9,2746E-01	-0,13
Q7TMK9	HNRPQ	9,2784E-01	-0,18
P25444	RS2	9,2785E-01	-0,13
Q8K3C3	LZIC	9,2828E-01	-0,14
Q9JLB0	MPP6	9,2833E-01	-0,09
Q62086	PON2	9,2855E-01	-0,06
Q9CPW4	ARPC5	9,2855E-01	-0,06
Q9R020	ZRAB2	9,2855E-01	-0,06
Q60931	VDAC3	9,2911E-01	-0,18
O88532	ZFR	9,2911E-01	-0,18
Q8VDM4	PSMD2	9,2994E-01	-0,17
P28474	ADHX	9,3002E-01	-0,13

P59325	IF5	9,3014E-01	-0,17
Q9CQJ8	NDUB9	9,3060E-01	-0,28
P31938	MP2K1	9,3070E-01	-0,16
Q8R570	SNP47	9,3074E-01	-0,17
P70404	IDHG1	9,3077E-01	-0,17
Q6NZF1	ZC11A	9,3112E-01	-0,06
P02468	LAMC1	9,3113E-01	-0,13
P17427	AP2A2	9,3126E-01	-0,13
Q9DBR1	XRN2	9,3143E-01	-0,17
O88441	MTX2	9,3157E-01	-0,17
O54931	AKAP2	9,3296E-01	-0,13
Q9CXI5	MANF	9,3308E-01	-0,17
Q8R1Q8	DC1L1	9,3335E-01	-0,17
Q8CGC6	RBM28	9,3356E-01	-0,06
Q9CWM4	PFD1	9,3356E-01	-0,06
P0C7N9	PSMG4	9,3377E-01	-0,06
Q60967	PAPS1	9,3406E-01	-0,10
P61290	PSME3	9,3412E-01	-0,17
Q9Z2X1	HNRPF	9,3417E-01	-0,17
Q8C1B7	40787	9,3435E-01	-0,14
Q8C7X2	EMC1	9,3481E-01	-0,12
Q6NZB0	DNJC8	9,3506E-01	-0,06
P62960	YBOX1	9,3514E-01	-0,17
P84228	H32	9,3514E-01	-0,12
Q07797	LG3BP	9,3627E-01	-0,12
P06151	LDHA	9,3687E-01	-0,15
P85094	ISC2A	9,3751E-01	-0,16
Q9Z1S8	GAB2	9,3754E-01	-0,15
P68037	UB2L3	9,3768E-01	-0,26
Q9CQF9	PCYOX	9,3802E-01	-0,14
Q9CQT2	RBM7	9,3858E-01	-0,09
Q9CWR0	ARHGP	9,3858E-01	-0,09
P21845	TRYB2	9,3858E-01	-0,09
Q6P8M1	TATD1	9,3858E-01	-0,09
Q9D8S9	BOLA1	9,3858E-01	-0,09
Q9R207	NBN	9,3858E-01	-0,09
Q8BGQ1	SPE39	9,3858E-01	-0,09
O70293	GRK6	9,3858E-01	-0,09
Q9JHE7	TSSC4	9,3858E-01	-0,09
P28063	PSB8	9,3868E-01	-0,11
Q91WK5	GCSH	9,3882E-01	-0,06
Q9ET30	TM9S3	9,3894E-01	-0,16
Q61171	PRDX2	9,3914E-01	-0,14

Q8BVA5	LDAH	9,3958E-01	-0,04
O89090	SP1	9,3958E-01	-0,04
Q61647	HYAS1	9,3958E-01	-0,04
Q08775	RUNX2	9,3958E-01	-0,04
Q99J27	ACATN	9,3958E-01	-0,04
P70665	SIAE	9,3958E-01	-0,04
Q8QZV7	INT13	9,3958E-01	-0,04
Q99KG5	LSR	9,3958E-01	-0,04
P17897	LYZ1	9,3958E-01	-0,04
P47758	SRPRB	9,3958E-01	-0,11
Q6DIC0	SMCA2	9,3987E-01	-0,16
P97370	AT1B3	9,4039E-01	-0,16
Q9D8T2	GSDMD	9,4043E-01	-0,14
P43275	H11	9,4054E-01	-0,16
Q8BTS4	NUP54	9,4067E-01	-0,09
Q61830	MRC1	9,4068E-01	-0,16
Q80W00	PP1RA	9,4068E-01	-0,16
Q05186	RCN1	9,4120E-01	-0,13
O35382	EXOC4	9,4135E-01	-0,16
Q9ERE7	MESD	9,4178E-01	-0,11
Q61990	PCBP2	9,4186E-01	-0,12
Q62095	DDX3Y	9,4364E-01	-0,12
Q8BTX9	HSDL1	9,4387E-01	-0,15
Q9DCM0	ETHE1	9,4387E-01	-0,15
A2A8U2	TM201	9,4433E-01	-0,26
P40124	CAP1	9,4517E-01	-0,17
O35350	CAN1	9,4558E-01	-0,17
Q9D172	GAL3A	9,4569E-01	-0,16
Q9WTL7	LYPA2	9,4594E-01	-0,15
Q8CCJ3	UFL1	9,4595E-01	-0,15
Q9CY66	GAR1	9,4660E-01	-0,15
P09671	SODM	9,4671E-01	-0,12
P62874	GBB1	9,4693E-01	-0,17
P18654	KS6A3	9,4839E-01	-0,17
P55002	MFAP2	9,4850E-01	-0,15
Q8BJU0	SGTA	9,4858E-01	-0,12
Q91VD9	NDUS1	9,4861E-01	-0,17
Q9EQU5	SET	9,4880E-01	-0,13
O70423	AOC3	9,4885E-01	-0,14
Q91XD6	VPS36	9,4901E-01	-0,09
P62317	SMD2	9,4925E-01	-0,13
Q61292	LAMB2	9,4992E-01	-0,25
Q9D898	ARP5L	9,5036E-01	-0,15

Q8K0Z7	TACO1	9,5036E-01	-0,04
P15655	FGF2	9,5036E-01	-0,04
Q8VE18	SMG8	9,5036E-01	-0,04
Q64516	GLPK	9,5036E-01	-0,04
Q8K2L8	TPC12	9,5036E-01	-0,04
Q921Z5	TFIP8	9,5036E-01	-0,04
Q9QZ85	IIGP1	9,5072E-01	-0,68
O35609	SCAM3	9,5072E-01	-0,15
Q8JZR0	ACSL5	9,5075E-01	-0,09
P63087	PP1G	9,5117E-01	-0,16
O88322	NID2	9,5133E-01	-0,16
B7ZMP1	XPP3	9,5141E-01	-0,15
Q64337	SQSTM	9,5155E-01	-0,16
P62996	TRA2B	9,5189E-01	-0,13
Q99KE1	MAOM	9,5198E-01	-0,17
P59279	RAB2B	9,5236E-01	-0,12
P58871	TB182	9,5277E-01	-0,07
Q61233	PLSL	9,5346E-01	-0,15
O35737	HNRH1	9,5398E-01	-0,15
Q9Z277	BAZ1B	9,5401E-01	-0,44
Q9WTZ1	RBX2	9,5402E-01	-0,04
Q8C6E0	CFA36	9,5402E-01	-0,04
P97364	SPS2	9,5402E-01	-0,04
Q9R1Z8	VINEX	9,5412E-01	-0,09
Q8BFQ8	GALD1	9,5423E-01	-0,15
Q9CQD1	RAB5A	9,5425E-01	-0,13
Q8K157	GALM	9,5428E-01	-0,13
Q80XI4	PI42B	9,5434E-01	-0,15
Q8BK72	RT27	9,5436E-01	-0,11
P61358	RL27	9,5437E-01	-0,11
Q9D7X8	GGCT	9,5463E-01	-0,15
P48758	CBR1	9,5506E-01	-0,17
Q9ERS2	NDUAD	9,5520E-01	-0,11
Q6PB66	LPPRC	9,5534E-01	-0,15
Q8CI04	COG3	9,5544E-01	-0,09
Q8VC42	RMC1	9,5544E-01	-0,09
Q9D8X2	CC124	9,5544E-01	-0,09
Q9CPW7	ZMAT2	9,5544E-01	-0,09
Q9JLJ5	ELOV1	9,5544E-01	-0,09
Q8BG94	COMD7	9,5544E-01	-0,09
Q99KU0	VMP1	9,5544E-01	-0,09
Q5SF07	IF2B2	9,5544E-01	-0,09
Q61009	SCRB1	9,5550E-01	-0,16

P28658	ATX10	9,5552E-01	-0,14
Q9CQM9	GLRX3	9,5630E-01	-0,13
Q9JLQ0	CD2AP	9,5654E-01	-0,15
Q99KG3	RBM10	9,5660E-01	-0,09
Q0VBL3	RBM15	9,5664E-01	-0,14
Q3UNZ8	QORL2	9,5670E-01	-0,12
Q61768	KINH	9,5671E-01	-0,17
Q61510	TRI25	9,5673E-01	-0,13
Q63961	EGLN	9,5704E-01	-0,09
Q8K3X4	I2BPL	9,5711E-01	0,01
O55029	COPB2	9,5734E-01	-0,17
Q61464	ZN638	9,5738E-01	-0,09
P99026	PSB4	9,5783E-01	-0,14
Q8VDJ3	VIGLN	9,5861E-01	-0,17
P84096	RHOG	9,5889E-01	-0,11
Q9CQ19	MYL9	9,5913E-01	-0,14
Q9WTU6	MK09	9,5932E-01	-0,11
P53026	RL10A	9,5934E-01	-0,15
P83882	RL36A	9,5935E-01	-0,13
Q8BHS6	ARMX3	9,5977E-01	-0,15
Q64437	ADH7	9,6004E-01	-0,26
P54728	RD23B	9,6051E-01	-0,16
Q9QZ88	VPS29	9,6060E-01	-0,12
Q9QZM0	UBQL2	9,6086E-01	-0,13
Q9D1E6	TBCB	9,6175E-01	-0,15
Q921E2	RAB31	9,6183E-01	-0,11
Q61207	SAP	9,6213E-01	-0,16
Q9Z0S1	BPNT1	9,6321E-01	-0,16
P62746	RHOB	9,6341E-01	-0,03
Q3TB82	PKHF1	9,6345E-01	-0,13
Q9D7J9	ECHD3	9,6360E-01	-0,13
O88384	VTI1B	9,6365E-01	-0,09
Q8R1A4	DOCK7	9,6365E-01	-0,09
O08746	MATN2	9,6369E-01	-0,36
Q07113	MPRI	9,6369E-01	-0,36
Q8K2C7	OS9	9,6369E-01	-0,36
Q9ESW4	AGK	9,6424E-01	-0,12
P32261	ANT3	9,6459E-01	-0,17
Q9CQ80	VPS25	9,6480E-01	-0,04
Q9D1J1	NECP2	9,6480E-01	-0,04
Q8K2K6	AGFG1	9,6486E-01	-0,15
Q8VE62	PAIP1	9,6489E-01	-0,15
Q91ZN5	S35B2	9,6510E-01	-0,09

O70152	DPM1	9,6578E-01	-0,15
P28653	PGS1	9,6584E-01	-0,17
Q8R0F8	FAHD1	9,6606E-01	-0,09
Q921J2	RHEB	9,6622E-01	-0,13
Q3TPE9	ANKY2	9,6695E-01	-0,09
Q8VDQ8	SIR2	9,6739E-01	-0,13
Q80YR5	SAFB2	9,6753E-01	-0,10
Q60676	PPP5	9,6766E-01	-0,13
Q8R3B1	PLCD1	9,6873E-01	-0,16
Q9JMG7	HDGR3	9,6899E-01	-0,13
Q9D711	PIR	9,6899E-01	-0,13
Q9D8V0	HM13	9,6905E-01	-0,16
Q64010	CRK	9,6921E-01	-0,14
Q8BH69	SPS1	9,6936E-01	-0,16
Q91V41	RAB14	9,6937E-01	-0,16
Q922Q1	MARC2	9,6941E-01	-0,14
Q7TT50	MRCKB	9,6960E-01	-0,21
Q9CYG7	TOM34	9,7007E-01	-0,11
P68373	TBA1C	9,7018E-01	-0,17
P46638	RB11B	9,7213E-01	-0,16
Q8BYI6	PCAT2	9,7216E-01	0,00
Q9Z0V7	TI17B	9,7252E-01	-0,14
Q8K221	ARFP2	9,7307E-01	-0,09
Q9QZE5	COPG1	9,7340E-01	-0,15
Q7TPR4	ACTN1	9,7393E-01	-0,16
O35963	RB33B	9,7394E-01	-0,11
O88958	GNPI1	9,7408E-01	-0,13
Q8BP56	PGGHG	9,7408E-01	-0,09
A6H584	CO6A5	9,7421E-01	0,26
P57776	EF1D	9,7429E-01	-0,16
Q80Y14	GLRX5	9,7464E-01	-0,11
P61924	COPZ1	9,7490E-01	-0,16
Q9D0I9	SYRC	9,7558E-01	-0,15
Q99KR3	LACB2	9,7581E-01	-0,13
Q9WVR4	FXR2	9,7599E-01	-0,15
Q99KC8	VMA5A	9,7637E-01	-0,15
Q9D967	MGDP1	9,7757E-01	-0,14
Q8VEH3	ARL8A	9,7818E-01	-0,14
Q9CZV8	FXL20	9,7841E-01	-0,53
Q91W90	TXND5	9,7900E-01	-0,16
P47968	RPIA	9,7997E-01	-0,11
Q9R1P1	PSB3	9,8009E-01	-0,14
Q8BGD9	IF4B	9,8032E-01	-0,15

P12815	PDCD6	9,8045E-01	-0,14
Q9CPR4	RL17	9,8049E-01	-0,14
Q9CYA0	CREL2	9,8053E-01	-0,14
Q8VCF0	MAVS	9,8054E-01	-0,14
P41778	PBX1	9,8058E-01	-0,12
Q8BQ47	CNPY4	9,8067E-01	-0,14
P83917	CBX1	9,8073E-01	-0,12
Q61595	KTN1	9,8074E-01	-0,16
P70677	CASP3	9,8076E-01	-0,11
Q62167	DDX3X	9,8086E-01	-0,16
P43406	ITAV	9,8091E-01	-0,16
Q9QZ23	NFU1	9,8118E-01	-0,09
Q9WUD1	CHIP	9,8132E-01	-0,13
Q9CZ04	CSN7A	9,8166E-01	-0,14
Q9QY30	ABCBB	9,8196E-01	-0,10
Q61074	PPM1G	9,8226E-01	-0,13
Q03173	ENAH	9,8327E-01	-0,14
Q9CY27	TECR	9,8327E-01	-0,14
Q91X96	MSS4	9,8331E-01	-0,16
Q8C3X2	CC90B	9,8331E-01	-0,16
Q9Z1Z2	STRAP	9,8381E-01	-0,16
Q8C3X8	LMF2	9,8440E-01	-0,16
Q8C1A5	THOP1	9,8487E-01	-0,16
P39054	DYN2	9,8535E-01	-0,16
Q9WTM5	RUVB2	9,8598E-01	-0,16
Q8BZR9	NCBP3	9,8600E-01	-0,09
P50096	IMDH1	9,8607E-01	-0,10
P28654	PGS2	9,8619E-01	-0,16
Q9CQS5	RIOK2	9,8647E-01	-0,53
Q3UJP5	CH037	9,8668E-01	-0,13
P61965	WDR5	9,8698E-01	-0,15
Q9R111	GUAD	9,8790E-01	-0,12
P52795	EFNB1	9,8806E-01	-0,09
Q99NH0	ANR17	9,8809E-01	-0,64
Q8CCF0	PRP31	9,8854E-01	-0,15
P61089	UBE2N	9,8914E-01	-0,15
Q9CPU0	LGUL	9,8916E-01	-0,15
Q9D1C8	VPS28	9,8928E-01	-0,09
P26450	P85A	9,8928E-01	-0,09
P14148	RL7	9,8962E-01	-0,14
Q9Z2I0	LETM1	9,8969E-01	-0,15
P62715	PP2AB	9,8992E-01	-0,15
Q00519	XDH	9,9015E-01	-0,26

Q8VDC0	SYLM	9,9079E-01	-0,16
P32921	SYWC	9,9099E-01	-0,16
Q00422	GABPA	9,9222E-01	-0,13
Q8BFZ3	ACTBL	9,9260E-01	-0,15
Q9QUM9	PSA6	9,9353E-01	-0,16
P70279	SURF6	9,9364E-01	-0,04
P46662	MERL	9,9391E-01	-0,09
O08664	BCL7C	9,9391E-01	-0,09
Q9DCR2	AP3S1	9,9414E-01	-0,09
Q9JJI8	RL38	9,9414E-01	-0,09
P99028	QCR6	9,9414E-01	-0,09
Q9Z1D1	EIF3G	9,9426E-01	-0,15
Q99KX1	MLF2	9,9450E-01	-0,16
Q8BGK6	YLAT2	9,9452E-01	-0,09
E9Q6P5	TTC7B	9,9481E-01	-0,12
P54775	PRS6B	9,9514E-01	-0,16
Q8BFV2	PCID2	9,9587E-01	-0,12
Q9CY50	SSRA	9,9590E-01	-0,12
Q8BYL4	SYYM	9,9602E-01	-0,09
Q9JK42	PDK2	9,9602E-01	-0,09
P42225	STAT1	9,9639E-01	-0,17
P21278	GNA11	9,9683E-01	-0,14
Q9D8Z1	ASCC1	9,9723E-01	-0,16
Q6P5G6	UBXN7	9,9727E-01	-0,12
Q7TNC4	LC7L2	9,9733E-01	-0,15
Q9QUH0	GLRX1	9,9749E-01	-0,09
Q8VEH6	CBWD1	9,9749E-01	-0,09
Q99JX4	EIF3M	9,9771E-01	-0,15
Q6P5H6	FRMD5	9,9773E-01	-0,14
P50518	VATE1	9,9773E-01	-0,14
Q62446	FKBP3	9,9818E-01	-0,12
P56399	UBP5	9,9835E-01	-0,16
Q8VDQ1	PTGR2	9,9839E-01	-0,13
P99024	TBB5	9,9852E-01	-0,16
Q8R2U6	NUDT4	9,9874E-01	-0,09
Q8K2F8	LS14A	9,9891E-01	-0,13
Q9ERR7	42248	9,9891E-01	-0,13
Q9D7G0	PRPS1	9,9924E-01	-0,14
P53612	PGTB2	9,9934E-01	-0,09
P68033	ACTC	1,0000E+00	0,00
P05213	TBA1B	1,0000E+00	0,00
Q9CWF2	TBB2B	1,0000E+00	0,00
P16858	G3P	1,0000E+00	0,00

Q6PIE5	AT1A2	1,0000E+00	0,00
Q61781	K1C14	1,0000E+00	0,00
Q9JI91	ACTN2	1,0000E+00	0,00
P21440	MDR3	1,0000E+00	0,00
P19639	GSTM3	1,0000E+00	0,00
Q61036	PAK3	1,0000E+00	0,00
O88643	PAK1	1,0000E+00	0,00
Q9QXY6	EHD3	1,0000E+00	0,00
P97427	DPYL1	1,0000E+00	0,00
P84075	HPCA	1,0000E+00	0,00
P70694	DHB5	1,0000E+00	0,00
Q60996	2A5G	1,0000E+00	0,00
P14430	HA18	1,0000E+00	0,00
Q9JHW9	AL1A3	1,0000E+00	0,00
P06330	HVM51	1,0000E+00	0,00
P19246	NFH	1,0000E+00	0,00
P03995	GFAP	1,0000E+00	0,00

Table S5. Reproduction sub-network

UniprotKB Accession Number	Entry Name	Beta-binomial test	Log2 ratio (M vs C)
Q6B966	NLRP14	8,2531E-05	-3,63
Q9R1M5	NLRP5	1,4667E-05	-3,56
Q8K3V4	PADI6	1,3046E-05	-3,39
Q9WVB3	TLE6	1,1966E-05	-3,17
Q9CWU5	KHDC3	9,5966E-06	-2,93
Q9QXC1	FETUB	2,3427E-02	-1,44
Q9Z2C8	YBX2	2,0973E-02	-1,24
Q62005	ZP1	4,7864E-02	-1,12

Table S6. Exosome sub-network

EXOSC# are noted as EXOS# and EXOSC10 is noted EXOSX

UniprotKB Accession Number	Entry Name	Beta-binomial test	Log2 ratio (M vs C)
Q9JHI7	EHMT1	1,6493E-03	-2,63
Q9JJA4	EXOS9	1,1440E-03	-1,82
Q9D753	EXOS8	1,5063E-03	-1,75
O35490	EXOS7	4,0330E-03	-1,74
Q9D0M0	GRWD1	1,1860E-02	-1,68
Q8BGS0	WDR12	1,2482E-03	-1,53
P56960	UTP15	2,0386E-02	-1,44
Q810D6	EXOS3	1,4346E-02	-1,38
Q6A068	EXOSX	1,1518E-02	-1,32
Q7TQK4	MAK16	1,1382E-02	-1,26
Q8R3N1	CDC5L	1,3684E-02	-1,20
Q8C7V3	USP10	2,4809E-02	-1,19
P52479	CSTF3	3,1518E-02	-1,19
Q99LI7	NOP14	1,8999E-02	-1,14
Q921I9	EXOS4	3,4105E-02	-1,09
O55201	SUPT5	3,7863E-02	-1,04

Table S7

ID	Biological Process (EGO)	geneID	pvalue
GO:0006260	DNA replication	Pold2/Rrm1/Rrm2/Cdk1/Mcm3/Mcm4/Msh6/Mcm2/Mcm6/Egfr/Ctc1/Mcm7/Nup98/Grwd1/Ilkap/Wrnip1/Rfc4/Atg7/Rfc2	4.41e-13
GO:0071027	nuclear RNA, mRNA surveillance	Exosc10/Exosc3/Exosc4/Exosc7/Exosc8/Exosc9	6.45e-10
GO:0000460	maturation of 5,8 rRNA	Exosc10/Exosc3/Exosc4/Exosc7/Exosc8/Exosc9/Wdr12	1.90e-09
GO:0016075	rRNA catabolic and metabolic process	Exosc10/Spin1/Exosc3/Utp15/Nop14/Rrp1b/Exosc4/Exosc7/Exosc8/Nvl/Exosc9/Wdr12	9.76e-09
GO:0006281	DNA repair	Pold2/Mcm3/Msh2/Mcm4/Usp10/Msh6/Trp53bp1/Mcm2/Mcm6/Egfr/Mcm7/Cdc5l/Usp7/Ints3/Usp47/Smc2/Uhrf1/Wrnip1	1.36e-07
GO:0006913	nucleocytoplasmic transport	Thoc2/Nup153/Prkcd/Nup98/Txnip/Heatr3/Ipo11/Nup133/Camk1/Nup155/Gsk3b/Nup160/Sfn/Htatip2	2.33e-07
GO:0034470	ncRNA processing, metabolic and catabolic process	Rpp30/Exosc10/Thumpd3/Spin1/Ints3/Exosc3/Utp15/Nop14/Rrp1b/Exosc4/Exosc7/Exosc8/Nvl/Wdr4/Exosc9/Wdr12	2.78e-07
GO:0031123	RNA 3'-end processing	Supt5/Exosc10/Exosc3/Exosc4/Cstf3/Exosc7/Exosc8/Exosc9	2.62e-06
GO:0042254	ribosome biogenesis	Exosc10/Exosc3/Heatr3/Utp15/Nop14/Rrp1b/Exosc4/Exosc7/Exosc8/Nvl/Exosc9/Wdr12	7.31e-06
GO:0006399	tRNA metabolic process	Rpp30/Exosc10/Thumpd3/Exosc3/Exosc7/Exosc8/Wdr4/Exosc9	1.04e-04
GO:0031126	snoRNA 3'-end processing	Exosc10/Exosc3/Exosc4	1.33e-04
GO:0006337	nucleosome disassembly	Arid1a/Smarcc1/Grwd1	2.71e-04

Table S7. Classification and enrichment analysis by cluster profiling. Enrich Gene Ontology (EGO) analysis. Significantly enriched ($P<0.01$) biological process terms are listed.
